# Drought stress modulates cuticular wax composition of the grape berry

**DOI:** 10.1093/jxb/eraa046

**Published:** 2020-01-27

**Authors:** Nicolas Dimopoulos, Ricco Tindjau, Darren C J Wong, Till Matzat, Tegan Haslam, Changzheng Song, Gregory A Gambetta, Ljerka Kunst, Simone D Castellarin

**Affiliations:** 1 Wine Research Centre, Faculty of Land and Food Systems, The University of British Columbia, Vancouver, BC, Canada; 2 Department of Botany, The University of British Columbia, Vancouver, BC, Canada; 3 EGFV, Bordeaux-Sciences Agro, INRA, Univ. Bordeaux, ISVV, Villenave d’Ornon, France; 4 Fondazione Edmund Mach, Italy

**Keywords:** Cuticle, fruit, transpiration, triterpenoids, *Vitis vinifera* (grapevine), water deficit, wax esters

## Abstract

Drought events are a major challenge for many horticultural crops, including grapes, which are often cultivated in dry and warm climates. It is not understood how the cuticle contributes to the grape berry response to water deficit (WD); furthermore, the cuticular waxes and the related biosynthetic pathways are poorly characterized in this fruit. In this study, we identified candidate wax-related genes from the grapevine genome by phylogenetic and transcriptomic analyses. Developmental and stress response expression patterns of these candidates were characterized across pre-existing RNA sequencing data sets and confirmed a high responsiveness of the pathway to environmental stresses. We then characterized the developmental and WD-induced changes in berry cuticular wax composition, and quantified differences in berry transpiration. Cuticular aliphatic wax content was modulated during development and an increase was observed under WD, with wax esters being strongly up-regulated. These compositional changes were related to up-regulated candidate genes of the aliphatic wax biosynthetic pathway, including *CER10*, *CER2*, *CER3*, *CER1*, *CER4*, and *WSD1*. The effect of WD on berry transpiration was not significant. This study indicates that changes in cuticular wax amount and composition are part of the metabolic response of the grape berry to WD, but these changes do not reduce berry transpiration.

## Teaser

Water deficit caused an increase in grape berry aliphatic cuticular wax load and changes in wax composition, yet these modifications did not reduce the berry cuticle transpiration rate.

## Introduction

The plant cuticle covers all primary aerial organs forming the outermost layer of a plant’s ‘skin’, and is the interface between the plant and environment, protecting it from biotic and abiotic stresses (reviewed in Yeats and Rose, 2013). The cuticle is a specialized lipidic modification of plant cell walls, which is largely composed of a cutin polymer that acts as a macromolecular scaffold for cuticular waxes. These waxes are intercalated within the cutin polymer and deposited on the cuticle surface as epicuticular wax crystals or as a film (Yeats and Rose, 2013).

Cuticular waxes are primarily composed of very long chain (VLC) aliphatic compounds, and can also contain triterpenoids and other metabolites such as sterols and flavonoids (Bernard and Joubès, 2013). The cuticular aliphatic wax biosynthetic pathway synthesizes a range of VLC compounds, including fatty acids, primary alcohols, acyl esters (wax esters), alkanes, aldehydes, secondary alcohols, and ketones.

The first stage of cuticular aliphatic wax biosynthesis (Supplementary Fig. S1 at *JXB* online) involves the elongation of the fatty acyl-CoA-thioesters by the multienzyme fatty acid elongase (FAE) complex producing VLC fatty acids (VLCFAs) ranging from C_18_ to C_34_ in length. The FAE complex is composed of multiple enzymes which include a ketoacyl-CoA synthase (KCS), a β-ketoacyl-CoA reductase (KCR), a β-hydroxyacyl-CoA dehydratase, and an enoyl-CoA reductase (Haslam and Kunst, 2013*a*). In Arabidopsis, these four components of the FAE are encoded by the genes *CER6*, *KCR1*, *PAS2*, and *CER10*, respectively (Fiebig *et al.*, 2000; Zheng *et al.*, 2005; Bach *et al.*, 2008; Beaudoin *et al.*, 2009). Extension of VLCFAs past C_28_ in length requires the involvement of a subfamily of BAHD named CER2-LIKEs that function together with CER6 to produce longer acyl-CoA thioesters (Haslam *et al.*, 2012, 2015; Haslam and Kunst, 2013*a*).

VLCFA-CoAs are then modified to different wax components via two parallel metabolic pathways, which form primary alcohols and alkanes as their major products. In the alcohol-forming pathway, VLCFA-CoAs are reduced to alcohols by a fatty acyl-CoA reductase (FAR), CER4 (Rowland *et al.*, 2006). The alcohol products can then be esterified to fatty acids to generate wax esters (Li *et al.*, 2008). In the alkane-forming pathway, two related proteins, CER1 and CER3, function together to reduce VLCFA-CoAs to aldehydes, and then convert the aldehyde intermediates to alkanes by a catalytic process that remains very poorly understood (Bourdenx *et al.*, 2011; Bernard *et al.*, 2012).

The cuticle protects the plant from biotic and abiotic stresses, and water deficit (WD) has been of particular interest since the cuticle restricts water loss from plant surfaces. Seo *et al.* (2011) showed that the up-regulation of cuticular wax biosynthetic genes (including *KCS1*, *KCS2*, *KCS6*, *CER1*, and *WSD1*) accompanies a major increase in wax content in Arabidopsis in response to WD. Increases in wax content in response to WD have been observed in tobacco (Cameron *et al.*, 2006), Arabidopsis (Kosma *et al.*, 2009), sesame (Kim *et al.*, 2007), and poplar (Xu *et al.*, 2016). In all four cases, alkanes were the dominant aliphatic wax in the cuticle and showed the greatest accumulation upon WD.

There are substantial differences between the cuticles of vegetative tissues and those of fleshy fruits. Fruit cuticles are usually astomatous and considerably thicker than leaf cuticles (reviewed in Martin and Rose, 2014) and affect the post-harvest quality of fruits through their role as a barrier to dehydration and pathogens (reviewed in Petit *et al.*, 2017; Lara *et al.*, 2019). Large compositional diversity is found between cuticles of different fruit species (reviewed in Lara *et al.*, 2015) where compounds such as triterpenoids and flavonoids are also present, and the proportion of aliphatic waxes in the total cuticle can vary greatly, ranging from 5% in tomatoes (Domínguez *et al.*, 2009) to 30–50% in olives (Huang *et al.*, 2017).

The grape berry cuticle is rich in both aliphatic waxes and triterpenoids, like most fleshy fruits (Lara *et al.*, 2015). Oleanolic acid (OA) and its precursors (erythrodiol, β-amyrin) are the major triterpenoids on grapes (Radler, 1965). The cuticular triterpenoid and aliphatic wax content varies greatly between grapevine varieties; for example, OA was 42% of the total wax content in the Muscat d’Alsace berries and 80% in Sylvaner berries (Pensec *et al.*, 2014). Changes in cuticular waxes are possibly among the first signs of ripening in green berries as reported in a viticultural book of the 19th century (Lacoste, 1865). Interestingly, the total amount of chloroform-extracted cuticular waxes increases during early berry development and peaks before or at veraison (onset of ripening) (Rogiers *et al.*, 2004; Pensec *et al.*, 2014). The grape berry cuticle becomes thinner during ripening as the berry expands (Rogiers *et al.*, 2004), resulting in a decrease in wax content per unit area (Pensec *et al.*, 2014). The composition of berry cuticular waxes also changes with development. The relative OA content decreases with ripening as OA biosynthesis is outpaced by aliphatic wax synthesis (Pensec *et al.*, 2014).

Despite a negative relationship between cuticle thickness and post-harvest water loss, as was found in tomato mutants (Girard *et al.*, 2012), cuticle permeability also depends on its composition and its arrangements in various cuticle layers (Vogg *et al.*, 2004; Leide *et al.*, 2007; Jetter and Riederer, 2016). In the case of grape berries, both cuticular wax thickness and the water transpiration rate through the cuticle decrease over the course of berry development (Scharwies and Tyerman, 2017), which is not congruent with a simple correspondence between decreased cuticular wax thickness and increased transpiration (Rogiers *et al.*, 2004).

Grapes are often cultivated in dry and warm Mediterranean climates and subjected to prolonged droughts that limit berry growth (reviewed in Lovisolo *et al.*, 2016). Interestingly, genes annotated as wax ester synthases have been found to be up-regulated under WD in the grape berry (Savoi *et al.*, 2017). While the general pattern of development of the grape berry cuticle is known, there are unanswered questions. Does the aliphatic wax composition change over development, and, if so, how? How does the cuticular wax of the grape berry change and affect water loss from the berry in response to WD stress?

In the current study we hypothesized that WD would increase the biosynthesis of cuticular aliphatic wax in the grape berry cuticle and potentially decrease berry transpiration. To determine if this is indeed the case, we first conducted *in silico* analyses to identify candidate grapevine cuticular wax-related genes. Then we characterized the expression profiles of cuticular wax-related genes and assessed changes in cuticular wax content during berry development and under prolonged WD. Finally, these changes were related to rates of transpiration in control and WD berries.

## Materials and methods

### Identification of candidate cuticular wax-related genes in grapevine and *in silico* RNA sequencing meta-analysis

BLASTp searches were performed using BLAST+v2.3 (Camacho *et al.*, 2009). We used genes described in the published literature as query sequences (Supplementary Table S1) to search for (e-value cut-off: 1e^−10^) and identify all potential biosynthetic and transcription factor (TF) gene family members in the organism (*Arabidopsis thaliana*, *Medicago truncatula*, or *Vitis vinifera*) from which the query sequence came. Next, the potential family members were used as a query sequences in a second round of BLASTp searches to identify (e-value cut-off 1e^−10^) all potentially related homologs in grapevine and Arabidopsis. Protein sequences for Arabidopsis were retrieved from TAIR (www.arabidopsis.org) and those from grapevine were retrieved from the 12X V1 version of the genome (Jaillon *et al.*, 2007).

**Fig. 1.**
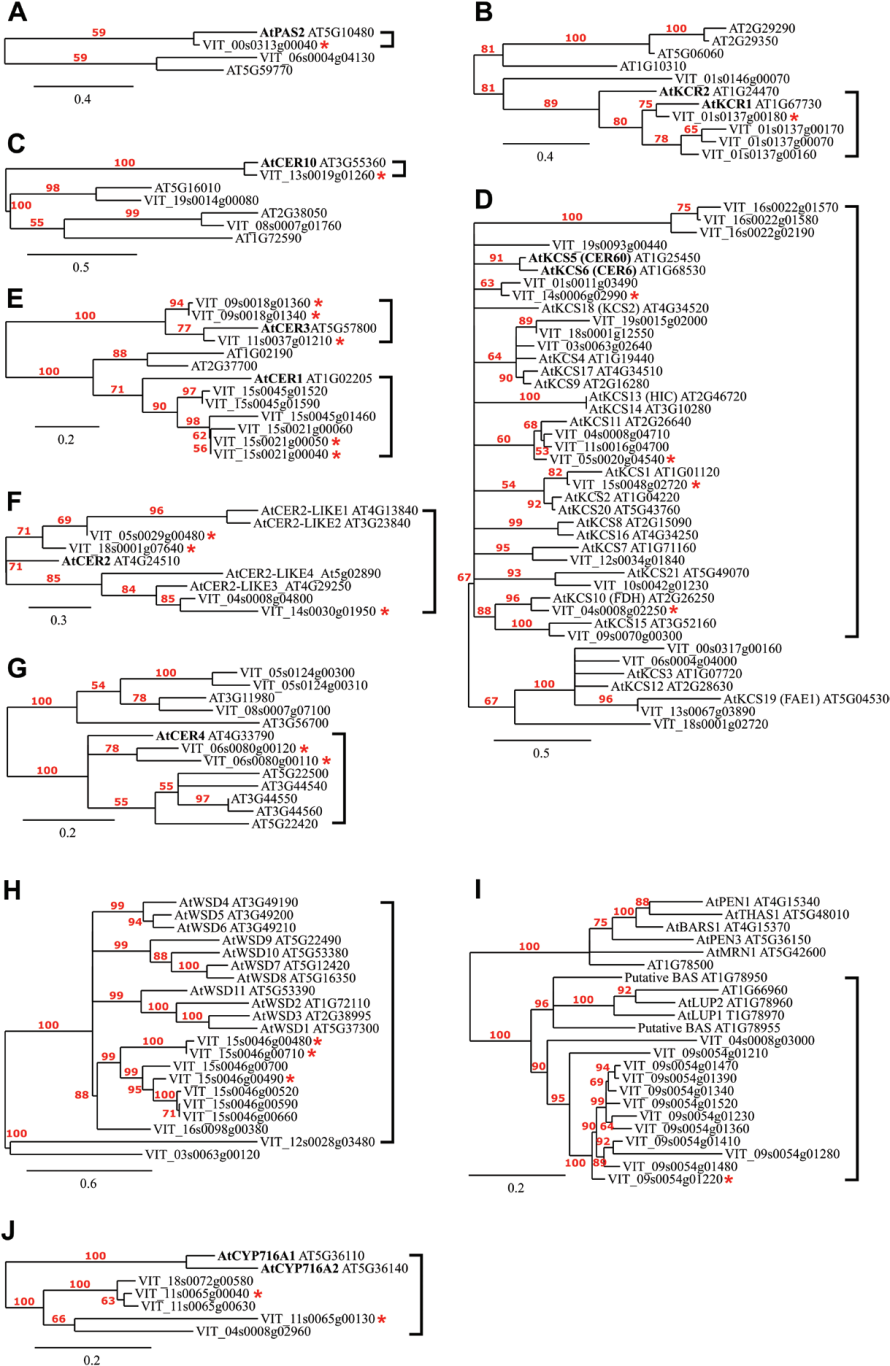
Protein sequence phylogenetic relationships of putative grapevine (*Vitis vinifera* L.) homologs of Arabidopsis biosynthetic genes involved in cuticular aliphatic wax biosynthesis. PAS2 (A), KCR1 (B), CER10 (C), KCS6 (D), and CER2 (F) are involved in fatty acid elongation. CER3 and CER1 (E) are part of the alkane-forming branch, and CER4 (G) and WSD1 (H) are part of the alcohol-forming branch. BAS (I) and CYP716A (J) are part of the oleanolic acid biosynthetic pathway. Numbers represent bootstrap values (100 bootstrap replicates), with branches of values <50 being collapsed together. Bold brackets denote clades that contain the characterized Arabidopsis sequences (in bold) involved in cuticular wax synthesis, and genes that are most closely related and are the likeliest functionally related homologs. Asterisks denote genes selected for study in the water deficit experiment. Scale bars are shown in each panel.

For further selection of biosynthetic gene candidates, multiple sequence alignment and dendrogram construction (Fig. 1) were carried out with Phylogeny.fr (Dereeper *et al.*, 2008). Sequences were aligned with MUSCLE (v3.8.31) using default settings. After alignment, gaps and/or poorly aligned regions were removed employing Gblocks (v0.91b) (Talavera and Castresana, 2007) using default settings, except for a minimum block length after gap cleaning of 5, and minimum number of sequences for a flank position=55%. Dendrograms were reconstructed using the maximum likelihood method implemented in the PhyML program (v3.1/3.0 aLRT) (Guindon *et al.*, 2010) with default settings. Reliability for the internal branch was assessed using 100 bootstrap replicates. Dendrograms were drawn with TreeDyn (v198.3) (Chevenet *et al.*, 2006).

Transcriptomic meta-analyses were conducted using publicly available data sets that examined different grapevine tissues, developmental stages, and genotypes, under several biotic and abiotic environmental stresses (Supplementary Table S2). Briefly, single-end and paired-end Illumina data sets were first processed by trimming for read quality and removal of adaptor sequences using Trimmomaticv0.36 (Bolger *et al.*, 2014) with the following settings: LEADING, 3; TRAILING, 3; SLIDINGWINDOW, 4:15; MINLEN, 40; AVGQUAL, 20. Trimmed reads were then aligned to the PN40024 12X grapevine genome (Jaillon *et al.*, 2007) using HISAT2v2.04 (Kim *et al.*, 2015) with default settings. For ABI SOLiD data sets, PASSv2.30 aligner (Campagna *et al.*, 2009) was used for quality trimming and alignment to the PN40024 12X grapevine genome with the following settings: -p 1111111001111111, -check_block 5000, -csfastq, -flc 1, -seeds_steps 3, -fid 90, -b, -l, -fle 40. Read count summarization was then performed on all the aligned reads with the grapevine V1 annotation (Jaillon *et al.*, 2007) using featureCounts (Liao *et al.*, 2014) on default settings.

EdgeR (Lun *et al.*, 2016) was used to calculate fragments/reads per kilobase of transcript per million mapped reads (FPKM/RPKM) transcript abundance (for differential expression (DE) analysis comparing the developmental stages or treatments (e.g. biotic and abiotic stresses) and controls. Significant DE was determined if the false discovery rate (FDR) was <0.05.

### Plant material and water deficit experiment

To assess the expression of candidate genes across grapevine tissues, we generated cDNA libraries from roots, stems, buds, apexes, leaves, tendrils, flowers, berry skins, berry flesh, and berry seeds of 2-year old Gewürztraminer (*V. vinifera* L.) grapevines at various developmental stages (Supplementary Table S3).

In order to investigate the impact of water deficit on cuticular waxes, a greenhouse experiment was carried out from 10 May to 5 August 2016 using 2-year-old own-rooted Merlot grapevines grown in 7.5 liter pots. Environmental light was supplemented by Phillips Green Power LED top lighting (spectra: deep red, medium blue, white lights) during the early phases of the experiment until 2 June 2016 in order to guarantee a minimum of 140 µmol m^−2^ s^−1^ of photosynthetically active radiation (PAR) at the table surface for 16 h per day. Two parallel irrigation treatments were applied, starting on 13 May at 30 days after anthesis (DAA) until the last sampling on 5 August at 113 DAA. Treatments consisted of control (CT) plants that were watered (nutrient-free water with dripper irrigation system) on a daily basis to maintain a leaf water potential above –0.8 MPa (Castellarin *et al.*, 2007*a*), and WD plants that were watered as needed to maintain an average leaf water potential between –1.6 MPa and –1.8 MPa that relates to severe water deficit for grapevines (Castellarin *et al.*, 2007*a*; Charrier *et al.*, 2018). Leaf water potential was measured at 14.00 h with a Scholander pressure chamber (PMS Instrument Company) according to Castellarin *et al.* (2007*b*). A range of 2–6 fully expanded leaves per treatment were measured weekly.

Five biological replicates were considered for each irrigation treatment, and each biological replicate consisted of a group of three vines, each with 1–3 developing clusters, for a total of 30 vines. In the greenhouse, the vines were spaced ~50 cm apart, and treatments and biological replicates were spatially arranged in a randomized manner. Vines were trimmed at the 18–20th node and secondary shoots were also removed from the plants throughout the experiment to maintain constant total leaf areas. Lastly, all other growing conditions were kept consistent regardless of the treatment and were based on the standard growing practices at the UBC Horticulture Greenhouse.

Six berries were collected for cuticular wax analysis from each biological replicate at 27 DAA (pre-treatment), 41 DAA (green berries), 68 DAA (mid-veraison), 82 and 96 DAA (ripening), and 111 DAA (late ripening). Another six berries were collected from each biological replicate at 10.30 h at the same developmental stages for performing gene expression analysis. An additional two berries were sampled from each biological replicate for SEM analyses at 28 DAA (pre-treatment), 42 DAA (green berries), 72 DAA (late veraison), 83 and 96 DAA (ripening), and 113 DAA (late ripening). Finally, 20 randomly selected berries from each treatment were sampled for measuring the rate of water transpiration at 28 DAA (pre-treatment), 48 DAA (green berries), 75 DAA (late veraison), 97 DAA (ripening), and 111 DAA (late ripening). At pre-treatment (27 and 28 DAA), berry samples were collected only from CT vines. During veraison (68, 72, and 75 DAA), one set of green berries (representing berries that have not started the ripening process) and another of red berries (representing berries that have started the ripening process) were collected for each of the analyses reported above.

In order to create little to no disturbance of the cuticular wax layer and avoid wiping of waxes from the berry surface during the sampling, berries were held with tweezers through the pedicel and carefully trimmed off the cluster with a pair of scissors. The berries for wax extraction and transpiration rates were then placed in 40 ml wide mouth glass test tubes, while those for gene expression analysis were placed in zip-lock bags. Berry development was tracked by measuring berry weight of the collected samples. Berry total soluble solids (TSS) were measured from the juice of berries collected for wax and RNA analyses with a digital refractometer (Sper Scientific).

### Cuticular wax extraction and quantification

Berry samples collected from the WD greenhouse experiment were brought to the laboratory just after sampling for wax extraction, which used an adapted version of the protocol described in Haslam *et al.* (2012) and in Haslam and Kunst (2013*b*). Pictures of the grape berries were taken before extraction, and their dimensions were measured using the GIMPv2.8.4 graphics editor. The two conjugate diameters of the berry were measured, and an average radius was calculated for each berry, from which the surface area was calculated with *A*=*4*π*r*^2^. The total surface area of a replicate was equal to the added surface areas of all the six berries in the replicate.

Grape berries were each submerged for 30 s with gentle swirling in 10 ml of chloroform with 10 µg of tetracosane as an internal standard. One chloroform bath for each replicate was performed using 40 ml wide mouth glass test tubes. Samples were then transferred to 11 ml glass screw-cap tubes, dried using an N_2_ gas stream and heat block (45 °C), and finally stored at –30 °C until ready for further processing.

Dried wax samples were resuspended in 930 µl of chloroform, after which 100 µl of the sample was aliquoted into an Agilent 250 µl vial insert and placed into an Agilent 2 ml wide opening screw-top vial. Samples where then dried under an N_2_ gas stream and heat block. Afterwards, 10 µl of pyridine and 10 µl of BSTFA+TMCS (99:1) were added to silylate hydroxyl and carboxylic acid groups, at 80 °C for 1 h. Samples were finally ready for GC-MS analysis after drying under an N_2_ gas stream and heat block, and then resuspended in 50 µl of chloroform.

For GC-MS analysis, a sample volume of 1 µl was injected into an Agilent Technologies 6890N (G1530N) GC using an Agilent Technologies J&W DB-1ms column (122-0132) with 30 m length, 250 µm diameter, and 0.25 µm film thickness. The GC used a pulsed splitless mode with a constant gas flow of helium and the following program: 45 °C for 2 min; ramp 45 °C min^–1^ to 210 °C; hold 1 min; ramp 5 °C min^–1^ to 340 °C; hold 24 min. The separated peaks were detected using an Agilent Technologies 5975 inert XL Mass Selective detector.

The GC-MS data were analyzed with Agilent Technologies MSD ChemStation E.01.01.335. Separated peaks were identified by comparing their fragmentation patterns with the Wiley Chemical Compound Library W9N08.L. Quantification was obtained by the construction of calibration curves of one representative standard for each class of compounds and using the internal standard (tetracosane) method. Hexacosane, hexacosanoic acid, hexacosanol, hexacosanal, behenyl palmitate, erythrodiol, and OA were used as representative standards for alkanes, fatty acids, alcohols, aldehydes, esters, and triterpenoids, respectively. The cuticular wax composition of each sample was then expressed in terms of wax (µg) per tissue surface area (cm^2^) extracted.

### RNA extraction and reverse tanscription–quantitative PCR (RT–qPCR) gene expression analysis

All tissues collected for the RNA extractions were snap-frozen and stored at –80 °C. Berry skins for the WD greenhouse experiment were peeled off after snap-freezing and dropped again in liquid nitrogen. Tissues were then ground into a fine powder in liquid nitrogen using a mortar and pestle and used for the RNA extraction (Wong *et al.*, 2016).

Total RNA was extracted from ~50 mg of powdered tissue using a Sigma Aldrich Spectrum™ Plant Total RNA Kit with the standard extraction protocol. RNA was then quantified using a Nanodrop (Thermo Scientific™ ND-1000 Spectrophotometer). Aliquots of 1 μg of RNA were digested with Thermo Scientific™ DNase I to remove genomic DNA, and cDNA was synthesized with a Thermo Scientific™ RevertAid First Strand cDNA Synthesis Kit.

Primers (Supplementary Table S4) for candidate genes were designed using the IDT PrimerQuest Tool (www.idtdna.com/primerquest) and NCBI Primer-BLAST (http://www.ncbi.nlm.nih.gov/tools/primer-blast). Expression of candidate genes relative to that of *VviUbiquitin* and *VviAP47*, for the cDNA tissue library and WD experiment, respectively, was measured by qPCR (Applied Biosystems 7500 Real Time PCR System; Life Technologies 7500 Software v2.0.6) using the 2^−ΔCT^ method (Savoi *et al.*, 2017).

### SEM

Berry sections were mounted onto SEM stubs, dried, and sputter coated using a Cressington 208HR High Resolution Sputter Coater with 12 nm Au/Pd. The images were taken using a Hitachi S-4700 Field Emission scanning electron microscope with 5 kV acceleration voltage at 10 µA and a working distance of 15 mm at a tilt of 34°. For the developmental time course, only CT berries (one berry per time point) were analyzed. For the comparison between CT and WD berries, only samples collected at 113 DAA (late ripening) were analyzed. Three biological replicates (one berry per biological replicate) per treatment were imaged. For each sample analyzed, images were taken from at least three different areas of the berry surface.

### Quantification of berry transpiration

The surface area dimensions and weight of the grapes (20 berries per treatment) were measured as described above. Berries were handled with care in order to minimally disturb the cuticular wax surface, and were placed in a sealed desiccation chamber with their cut pedicels sealed with Vaseline. Sealing with Vaseline was compared with paraffin wax—previously used for similar purposes by Rogiers *et al.* (2004)—and was determined to be equally effective (data not shown). In the desiccation chamber, the berries were left to dehydrate in the dark at constant temperature (23 °C) and relative humidity (32%) over the course of 7–9 d, with their weights measured daily (Rogiers *et al.*, 2004; Zhang and Keller, 2015). Relative humidity within the chamber was maintained constant with a saturated solution of MgCl_2_. The rate of water loss was then expressed in terms of water weight (g) per berry skin surface area (cm^2^) per hour as in Rogiers *et al.* (2004).

### Statistical analysis

Two-sample Student’s *t*-test in Microsoft Excel v15.40 was used to determine statistical significance (*P-value <0.05) between treatments (CT versus WD) at each time point for cuticular wax concentration, gene expression values, and transpiration rates of individual berries. A univariate repeated measures ANOVA test was performed with JMP v9 (© SAS Institute Inc.) to analyze the effect of the irrigation treatments (CT and WD) on water transpiration across the cuticle.

A Pearson correlation analysis and subsequent hierarchical clustering was performed using R (R Core Team, 2019) on gene expression and cuticular wax values from the water deficit experiment. All values were log(*x*+1) transformed for analysis.

## Results

### Identification and expression profiling of grapevine cuticular wax-related genes

Forty-six putative homologs in families related to cuticular aliphatic wax biosynthesis, and 17 involved in OA biosynthesis, were chosen based on their homology to previously characterized Arabidopsis genes and used for expression analyses (Fig. 1).

We examined the expression levels and DE patterns of the putative grapevine homologs in order to narrow down the list of candidates to the likeliest functionally relevant homologs. *In silico* analyses of the expression patterns of the identified candidate genes were performed examining their expression across different grapevine tissues: root, leaf, inflorescence, flower, berry (whole), and berry skin (Supplementary Fig. S2). We then validated the *in silico* expression results of the candidate genes we selected for study by *in vivo* RT–qPCR analysis in different grapevine tissues, which included root, stem, bud, shoot, leaf, tendril, berry skin and flesh, and seeds (Supplementary Fig. S3). The results confirmed the *in silico* analysis, showing that the candidate genes were mostly expressed in berry skin and leaves, and that seeds also strongly expressed aliphatic wax-related genes.

Additional *in silico* analyses examined the candidate response to abiotic and biotic stresses (Supplemenrtary Fig. S4). The effects of WD on the whole-berry transcriptome was investigated for Tocai Friulano and Merlot. The general trend of WD stress was an up-regulation of genes predicted to function in cuticular wax biosynthesis, and down-regulation of those predicted to be involved in OA biosynthesis. The two cultivars responded differently to WD stress; Tocai Friulano berries showed little change in expression, while Merlot berries experienced a much stronger response. In contrast to WD stress, both high temperature stress and *Botrytis cinerea* infection caused down-regulation of the majority of differentially expressed homologs that we associated with cuticular wax biosynthesis (Supplementary Fig. S4).

We picked (Supplementary Fig. S5) the likeliest fruit-specific functional homologs by selecting candidate genes that were the most closely related to the characterized biosynthetic genes (Fig. 1), and showed expression in grape tissue (Supplementary Fig. S2) and/or DE response to WD in Merlot grapes (Supplementary Fig. S4). This left 20 homologs (Supplementary Table S4) involved in cuticular wax synthesis and three homologs involved in OA synthesis for study in grape berry skin under WD stress. Additionally, the top BLASTp homologs for five transcription factors and an E3 ubiquitin ligase that regulate cuticular wax synthesis were also selected for study (Supplementary Table S4).

### Changes in berry cuticular wax composition during development and under water deficit

The grapevines exposed to WD consistently experienced lower leaf water potential, and WD berries were lower in weight and higher in concentration of soluble solids (Supplementary Fig. S6).

**Fig. 2.**
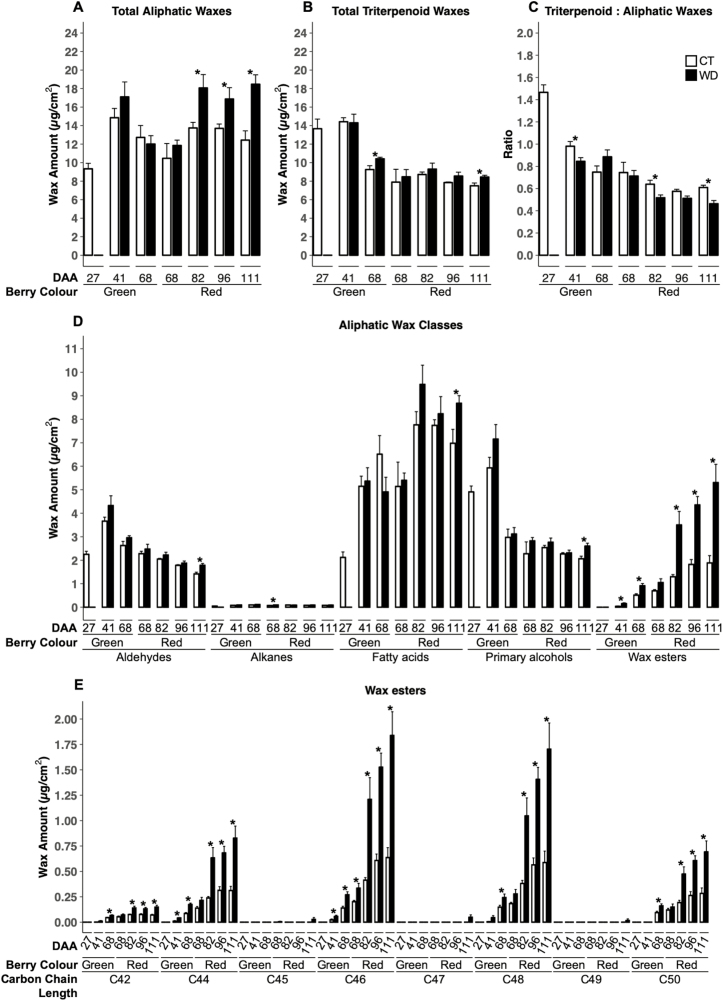
Cuticular wax composition in berries at 27, 41, 68, 82, 96, and 111 days after anthesis (DAA) of grapevines (*Vitis vinifera* L.) exposed to two irrigation treatments: well-irrigated (control, CT) and deficit irrigated (water deficit, WD). Total aliphatic waxes (A), total triterpenoid waxes (B), triterpenoids:aliphatic waxes ratio (C), individual functional group classes of aliphatic waxes (D), and wax ester composition (E) are reported. Please refer to Supplementary Figs S7–S11 for detailed wax composition of other functional group classes. At 68 DAA, green and red berries were analyzed independently. Error bars represent ±SE, and significant differences between treatments were determined by two-sample *t*-test (*P<0.05).

In CT berries, the aliphatic wax load per unit area (μg cm^–2^) (Fig. 2A) was highest at 41 DAA and then slightly decreased by the end of development (111 DAA) as the berry expanded. WD induced an increase of total aliphatic waxes at 82, 96, and 111 DAA. The triterpenoid load (Fig. 2B) followed a similar trend to that of aliphatic waxes. When the ratio of total triterpenoids to total aliphatic waxes was calculated (Fig. 2C), a clear trend was observed where the ratio was highest at 27 DAA and steadily decreased with berry development to stabilize during ripening. The ratio was lower in WD in both green and red berries.

When aliphatic waxes are separated into their functional classes (Fig. 2D), VLC primary alcohols and aldehydes were high during early berry development and decreased as berries ripened. In contrast, VLCFAs and wax esters increased as berries developed. Alkanes were the lowest of the aliphatic waxes and remained relatively stable throughout berry development and ripening.

Under WD treatment, alkanes were higher than in CT in red veraison berries (68 DAA), while VLCFAs, primary alcohols, and aldehydes were higher in late-ripening (111 DAA) berries. The amount of total wax esters was increased in response to WD at the majority of stages (41, 68 green, 82, 96, and 111 DAA) and involved mostly C_42_, C_44_, C_46_, C_48_, and C_50_ wax esters (Fig. 2E). The most abundant chain lengths of VLCFAs, aldehydes, and primary alcohols were greater in WD berries at late ripening (111 DAA) (Supplementary Figs S7–9), whereas C_21,_ C_23_, and C_25_ alkanes were found in greater amounts only in WD berries during veraison (68 DAA) (Supplementary Fig. S10). Higher OA content was also found in WD berries during ripening (68 green, 96, and 111 DAA) (Supplementary Fig. S11).

**Fig. 3.**
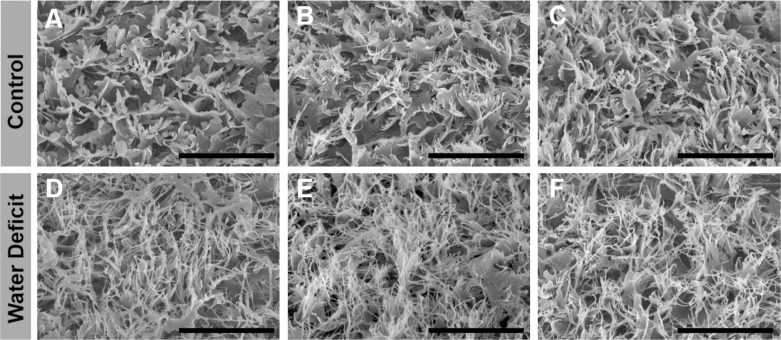
Ultrastructural morphology of cuticular waxes on grape berries at 113 days after anthesis (DAA). SEM images of the cuticular wax ultrastructure on grape berries of grapevines (*Vitis vinifera* L.) exposed to two irrigation treatments: (A–C) well-irrigated (control, CT); (D and E) deficit irrigated (water deficit, WD). Each image represents one biological replicate. The scale bar represents 5 µm in all images.

Reflecting these compositional changes, SEM images of epicuticular wax crystals showed that their structures changed with berry development (Supplementary Fig. S12). Crystal structures were also qualitatively different between treatments in late-ripening (113 DAA) berries, with CT crystals having a more ‘broad-leaf’-like shape and WD crystals having a more ‘spindly’-like shape (Fig. 3).

### Expression of candidate genes during development and under water deficit

**Fig. 4.**
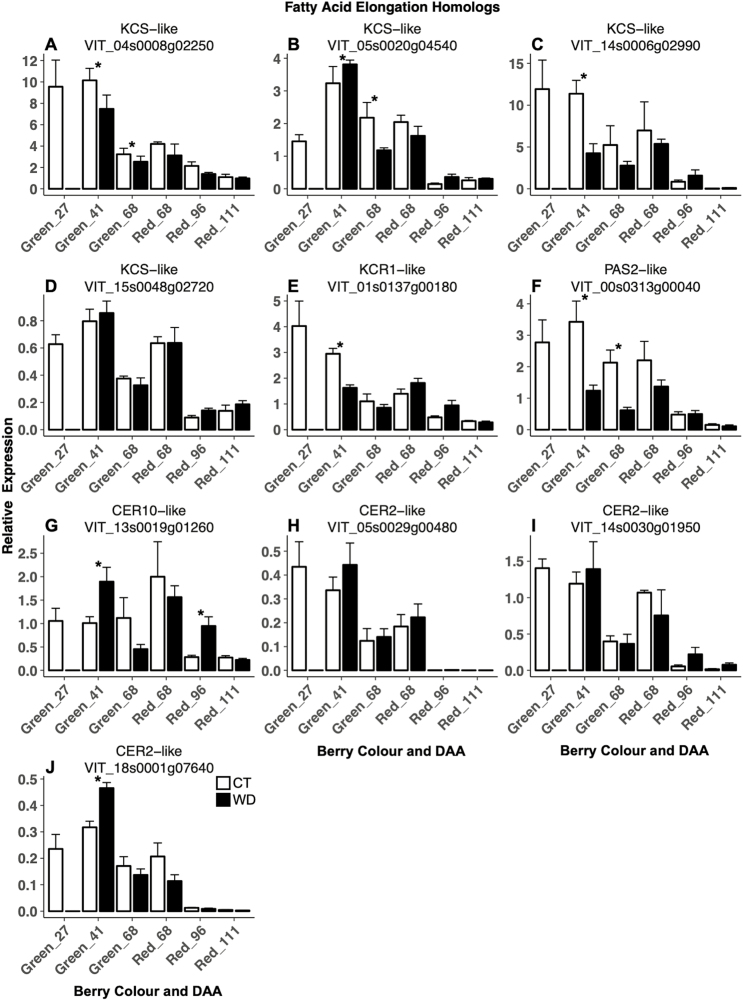
Expression of candidate genes involved in the fatty acid elongation of the biosynthetic cuticular wax pathway in berry skins of grapevines (*Vitis vinifera* L.) exposed to two irrigation treatments: well-irrigated (control, CT) and deficit irrigated (water deficit, WD). Error bars represent ±SE, and significant differences between treatments were determined by two-sample *t*-test (*P<0.05).

**Fig. 5.**
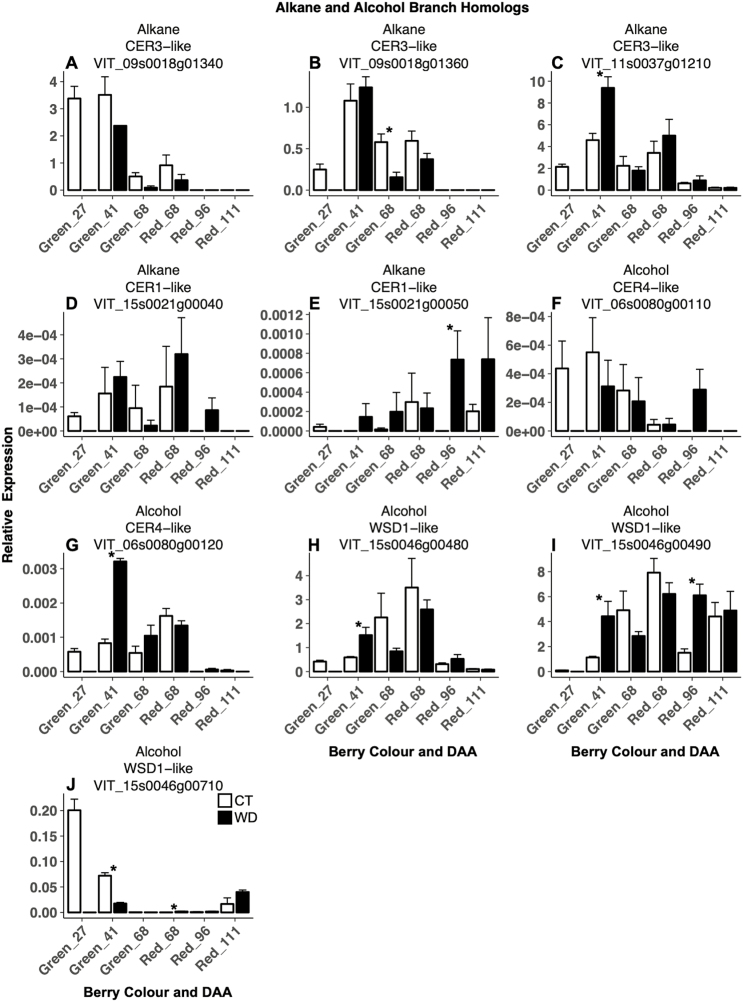
Expression of candidate genes involved in the alcohol- and alkane-forming branches of the biosynthetic cuticular wax pathway in berry skins of grapevines (*Vitis vinifera* L.) exposed to two irrigation treatments: well-irrigated (control, CT) and deficit irrigated (water deficit, WD). Error bars represent ±SE, and significant differences between treatments were determined by two-sample *t*-test (*P<0.05).

**Fig. 6.**
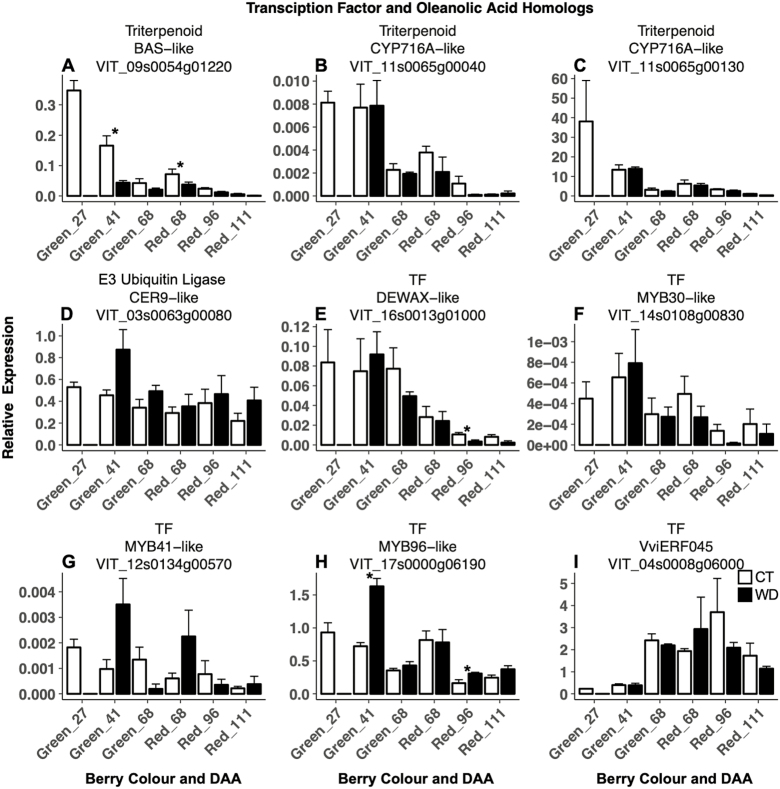
Expression of candidate genes involved in the oleanolic acid biosynthesis, and transcription factors (TFs) and an E3 ubiquitin ligase involved in regulating cuticular wax development of berry skins of grapevines (*Vitis vinifera* L.) exposed to two irrigation treatments: well-irrigated (control, CT) and deficit irrigated (water deficit, WD). Error bars represent ±SE, and significant differences between treatments were determined by two-sample *t*-test (*P<0.05).

Almost all of the selected genes putatively involved in aliphatic wax and OA biosynthesis and regulation exhibited the same expression pattern over the course of development in CT conditions (Figs 4–6) and had high correlation [Pearson correlation coefficent (PCC) ≥0.5] to each other along with aldehyde, primary alcohol, alkane, and triterpenoid levels (Supplementary Fig. S13). The homologs experienced the highest expression levels early in berry development (27 and 41 DAA), intermediate levels at veraison (green and red berries at 68 DAA), and lowest expression levels in later ripening stages (96 and 111 DAA). The exceptions to this expression pattern included a *CER1-*like (*VIT_*15s0021g00050), a *WSD1-like* (*VIT_15s0046g00490*), and *VviERF045* (*VIT_04s0008g06000*) that correlated into a separate group with VLCFAs and wax esters. These genes showed lower expression in early development and higher expression during ripening.

WD had an effect on the expression of many of the candidate genes tested. Among those involved in fatty acid elongation (Fig. 4), WD decreased the expression of three of the four *KCS-like* (*VIT_05s0020g04540* was both down-regulated and up-regulated at different times, and *VIT_15s0048g02720* was unaffected by WD), and of *KCR1-like* (*VIT_01s0137g00180*) and *PAS2-like* (*VIT_00s0313g00040*) homologs, but only in green berries. In contrast, WD increased the expression of the *CER10-like* (*VIT_13s0019g01260*) homolog and one *CER2-like* (*VIT_18s0001g07640*) homolog in green and red, and green berries, respectively.

Candidate genes associated with the alkane- and alcohol-forming pathways were affected by WD stress (Fig. 5). In green berries, the lowest (*VIT_09s0018g01360*) and highest (*VIT_11s0037g01210*) expressed *CER3-like* homologs were down-regulated and up-regulated by WD, respectively. *CER1-like* homologs experienced very low expression levels, though *VIT_15s0021g00050* was up-regulated by WD in red berries. Similarly, both *CER4-like* homologs had very low expression, with *VIT_06s0080g00120* being up-regulated by WD in green berries. All three *WSD1-like* homologs were affected by WD, *VIT_15s0046g00480* was up-regulated in green berries, *VIT_15s0046g00490* was up-regulated in both green and red berries, while *VIT_15s0046g00710* was down-regulated in green berries and then up-regulated in red berries at 68 DAA.

Of the five TFs we tested that are predicted to regulate aliphatic wax biosynthesis, WD affected the expression of two (Fig. 6). *DEWAX-like* (*VIT_16s0013g01000*) was down-regulated at 96 DAA and *MYB96-like* (*VIT_17s0000g06190*) was up-regulated at both 41 and 96 DAA.

One OA biosynthesis homolog (Fig. 6), *BAS-like* (*VIT_09s0054g01220*), was down-regulated by WD at 41 DAA and in red berries at 68 DAA.

### Transpiration rate through the berry cuticle

**Fig. 7.**
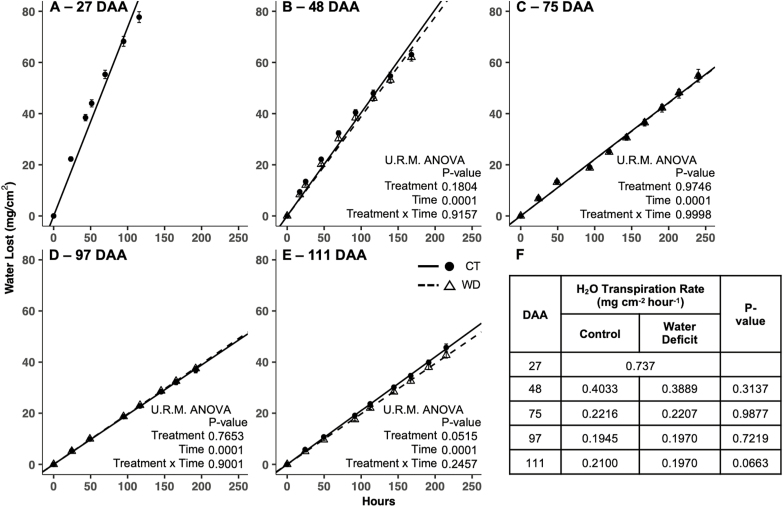
Rates of water transpiration of the whole berry at different stages of development in grapevines (*Vitis vinifera* L.) exposed to two irrigation treatments: well-irrigated (control, CT) and deficit irrigated (water deficit, WD). Stages of development that were measured were 27 (A), 48 (B), 75 (C), 97 (D), and 111 (E) days after anthesis (DAA). Error bars represent ±SE; significant differences in the cumulative amount of water lost for each developmental stage were determined by univariate repeated measures ANOVA (U.R.M. ANOVA). The effects of treatment, time, and their interaction are reported. In the table (F), the average transpiration rate and the significance value of the *t*-test at each developmental stage are reported.

The rate of water transpiration through the cuticle decreased as berries developed, then remained stable from 97 DAA to the end of the experiment (Fig. 7). There was no significant difference in the average rate of water transpiration (mg cm^−2^ h^–1^) through the berry cuticle between WD and CT treatments at any developmental stage tested. Moreover, when the cumulative amount of water lost per skin area (mg cm^−2^) at each measurement time (hours from *T*_0_), within each experiment (developmental stage: 48, 75, 97, and 111 DAA) was considered, no differences between treatments were seen.

## Discussion

### Evolution of the wax profile during berry development

The Merlot grape berry cuticular wax has a large triterpenoid content like other fleshy fruits (Lara *et al.*, 2015), a similar aliphatic wax class composition to other grapevine varieties (Radler, 1965), and a chain length distribution of aliphatic waxes very similar to that of other grapes (Radler, 1965) and plant species (Cameron *et al.*, 2006; Kim *et al.*, 2007; Kosma *et al.*, 2009). The decrease in total wax amount and triterpenoid content observed during berry maturation is typical of grape berries (Commenil *et al.*, 1997; Pensec *et al.*, 2014), and is common, though not universal, among other fruit species. In contrast, tomato cuticles progressively accumulate triterpenoids throughout fruit development (Lara *et al.*, 2015).

The onset of ripening (veraison) is a pivotal phenological stage in the regulation of the cuticular aliphatic wax biosynthetic pathway. The shift in wax composition suggests major changes in the expression of the associated biosynthetic genes occurring at veraison, which was observed. Almost all aliphatic wax and OA biosynthetic genes decreased in expression, while at the same time *WSD1-like* homologs and *VviERF045* increased in expression. These changes in wax metabolism at the onset of ripening are consistent with several major changes of berry metabolism and in gene expression previously reported (Wong *et al.*, 2016).

The amounts of OA and the expression of predicted biosynthetic genes early in development quickly decreased from veraison onwards, which is consistent with previous work reporting that OA accumulation occurs before veraison (Pensec *et al.*, 2014). In addition, Fukushima *et al.* (2011) had functionally characterized cytochrome P450 enzymes that produced OA in grapevine, but the loci IDs that they provided (*GSVIVT01032218001* and *GSVIVT01032223001* for CYP716A17 and CYP716A15, respectively) do not match their characterized proteins. Based on our analyses, we propose that instead, CYP716A17 is *VIT_11s0065g00130* (100% alignment) and that CYP716A15 is probably *VIT_11s0065g00040* since they are a very close match.

*VviERF045* has been proposed to be a key regulator in berry ripening (Palumbo *et al.*, 2014), and has been shown, using transgenic grapevine lines, to regulate expression of several grapevine genes putatively involved in cuticular wax biosynthesis (Leida *et al.*, 2016). Specifically, the *WSD1-like* homolog *VIT_15s0046g00490* was up-regulated when *VviERF045* was overexpressed (Leida *et al.*, 2016). In our experiment, there was a strong correlation (PCC ≥0.5) (Supplementary Fig. S13) between the expression of *VviERF045*, *VIT_15s0046g00490*, and total wax ester and VLCFA content during berry development. This correlation supports the idea that *VviERF045* is a key regulator that controls the shift in the cuticular aliphatic wax pathway towards increased VLCFA and wax ester synthesis from veraison onwards during berry development.

### Changes in the berry cuticular waxes under water deficit

Our *in silico* transcriptomic analysis indicated that cuticular wax-related genes were differentially expressed in grape berries exposed to water stress; particularly in Merlot grapes, which showed general up-regulation of the genes associated with the aliphatic cuticular wax biosynthetic pathway, and down-regulation of genes associated with OA biosynthesis. Not all grapevine cultivars behaved in the same way. For example, Tocai Friulano grape berries exposed to WD lacked expression of cuticular wax-related genes, suggesting differences, possibly related to their native eco-physiological niche, in the cuticle response between varieties analogous to differences observed for other metabolic and physiological responses to WD (Hochberg *et al.*, 2013*a*, *b*; Levin *et al.*, 2019).

Prolonged WD stress resulted in smaller berries with higher total soluble solids, as previously observed (Castellarin *et al.*, 2007*a*). This decrease in size was also associated with an increase in cuticular wax content. It is unlikely that this increase resulted from the decreased surface area of WD berries without changes in wax biosynthesis. If it had, we would expect to see increases in all cuticular waxes, and an unchanged total triterpenoids/total aliphatic waxes ratio between treatments. This was not the case, as the ratio was lower and some cuticular waxes were not affected by WD, such as alkanes (Supplementary Fig. S10), β-amyrin, and erythrodiol (Supplementary Fig. S11).

We observed several instances where wax compositional changes in WD berries did not correspond to the expression of the associated biosynthetic genes. Aliphatic wax content increased, while genes of the FAE complex were down-regulated. Increases in alkane content were observed at veraison, but *CER3-like* or *CER1-like* genes were not up-regulated at that point.

The most striking inconsistency was that primary alcohol content increased in late-ripening WD berries, when *CER4-like* expression was not detected. This indicates that primary alcohol synthesis and accumulation continued while the tested *CER4-like* homologs were absent. More distantly related *CER4* homologs were not expressed according to our *in silico* analysis (data not shown), suggesting that the gene responsible for primary alcohol synthesis was not identified. We searched for any additional homologs by a BLASTp search of the V2 grapevine genome annotation (Vitulo *et al.*, 2014), a tBLASTn search directly in the 12X grapevine genome (Jaillon *et al.*, 2007), and tBLASTn searches of Tannat (Da Silva *et al.*, 2013) and Merlot (Wong *et al.*, 2016) *de novo* assembled transcriptomes. None of these searches found any additional homologs.

Wax esters increased under WD by >2.5 times the CT levels by harvest time. This preference for wax esters contrasts with other species where alkanes are primarily increased under WD stress (Cameron *et al.*, 2006; Kim *et al.*, 2007; Kosma *et al.*, 2009; Xu *et al.*, 2016). The up-regulation of two *WDS1-like* homologs before and after veraison corresponded to the times when wax ester amounts were increased in WD berries. Additionally, the strong correlation (PCC ≥0.5) (Supplementary Fig. S13) seen between wax ester content and *VIT_15s0046g000490* expression make this gene the likeliest *WSD1-like* functional homolog.

Based on the effect of WD on their expression, the DE of *MYB96-like* and *DEWAX-like* homologs indicates that they may modulate the cuticular wax pathway under WD in the grape berry, similar to their counterparts in Arabidopsis (Oshima *et al.*, 2013; Go *et al.*, 2014; Cui *et al.*, 2016; Lee *et al.*, 2016).

### Water transpiration through the berry cuticle

Cuticular aliphatic waxes have previously been demonstrated to impede water transpiration through an artificial membrane and are responsible for forming the water-impermeable barrier on grape berries, whereas OA does not contribute to berry cuticular impermeability (Grncarevic and Radler, 1971; Casado and Heredia, 1999). Thus, one would hypothesize that WD berries should have experienced a lower transpiration rate once they start to accumulate greater amounts of waxes, but instead no decrease in transpiration was observed in WD berries during ripening or at harvest. Experiments with the response to WD stress in Arabidopsis (Kosma *et al.*, 2009; Seo *et al.*, 2011; Patwari *et al.*, 2019), tobacco tree (Cameron *et al.*, 2006), and wheat (Bi *et al.*, 2017) leaves revealed a decrease in the rate of water loss accompanying an increase of cuticular aliphatic wax amount. In contrast, the minor changes in the transpiration rate in this study suggest that the changes in cuticular wax are not associated with decreased berry water loss under water deficit.

The majority of the variables affecting water transpiration through the cuticle were controlled for in these experiments in order to have a high certainty that any water loss was through the cuticle. Water loss through berry stomata should be negligible since they are found at a much lower density than on leaves, and are sealed with cuticular wax shortly after anthesis (Palliotti and Cartechini, 2001). Additionally, the berries were kept in the dark during the transpiration experiments to induce the closure of any potentially functional stomata. Water loss through the cut pedicel was eliminated by sealing the wound.

Water loss in fruits depends on waxes (reviewed in Petit *et al.*, 2017; Lara *et al.*, 2019), but it does not always correlate to the total amount of cuticular waxes (López-Castañeda *et al.*, 2010; Parsons *et al.*, 2013). The amount of specific aliphatic waxes, such as alkanes, and the ratio of alkanes to total non-aliphatic wax compounds can have a strong effect on cuticular water loss, as seen in peppers (Parsons *et al.*, 2013). The importance of wax composition was confirmed using tomato mutants, where a decrease in the proportion of *n*-alkanes of chain lengths longer than C_28_ and a concomitant increase in cyclic triterpenoids increased water loss (Leide *et al.*, 2007). In contrast, in our experiment, alkanes were mostly absent from the cuticle and, based on the changes in transpiration and wax composition during development, there appears to be a strong relationship between berry water loss and VLCFA and wax ester content.

Furthermore, other components of the wax cuticle might also affect cuticular water permeability. In tomato, cutin content does not affect transpiration (Isaacson *et al.*, 2009), but flavonoid accumulation in the cuticle appears to modulate wax deposition during ripening and subsequently cuticular water transpiration, as well as other biomechanical properties (Luque *et al.*, 1995; España *et al.*, 2014*a*). In our study, we focused on cuticular waxes and we did not consider changes in the cutin matrix. However, based to the normally observed overexpression of flavonoid genes (Castellarin *et al.*, 2007*b*; Savoi *et al.*, 2017), we might expect increased flavonoid content in response to WD; yet, the potential presence of flavonoids in the cuticle structure did not result in reduced water transpiration.

Since no significant change in berry transpiration in response to WD was observed, it brings into question the biological role of the increased wax content in WD berry cuticles. One explanation could be that the berry response is part of a systemic response (Kachroo and Robin, 2013) to WD stress, where cuticular wax load is globally increased to reduce transpiration rates, regardless of whether the change in wax load is effective on any particular organ. On a whole-plant level, this perspective is especially relevant where maximum berry transpiration levels have been shown to be ~0.07 mmol H_2_O m^−2^ s^−1^ (corresponding to ~0.45 mg H_2_O cm^−2^ h^−1^) (Zhang and Keller, 2015); such levels are much lower than those of the canopy, where rates are >50 times higher (Rogiers *et al.*, 2009). When considering how much more canopy surface area there is relative to berries, the contribution of berry water loss to the whole plant is negligible.

Another explanation could be that the change in wax load has other important biological roles in berries, possibly protecting the berry cuticle against other unaccounted for environmental stresses.

Higher light conditions can occur on the more exposed WD clusters (Castellarin *et al.*, 2007*b*), potentially increasing berry temperature. A greater wax amount could possibly result in higher light reflectance (Holmes and Keiller, 2002; Heredia-Guerrero *et al.*, 2018) and/or heat capacity of the berry (Heredia-Guerrero *et al.*, 2018), and thus could be reducing the higher light and temperature stress the berries would be experiencing. Alternatively, modulating wax content under WD could also help protect berries from pathogens since OA, which increased in concentration under WD, has antimicrobial properties (Pensec *et al.*, 2014). Consistently, a correlation between resistance to *B. cinerea* infection and wax density/surface area on berries was found (Commenil *et al.*, 1997), and grape response to *B. cinerea* involves the triggering of cutin and cuticular wax biosynthesis and affected the level of several cuticular compounds as well as of other secondary metabolites such as *trans*-resveratrol and gallic acid (Agudelo-Romero *et al.*, 2015).

### Effects of cuticular wax on fruit quality

Besides the role in water loss, the cuticle also affects the fruit susceptibility to pathogen infections, fruit firmness and texture, and fruit appearance (reviewed in Lara *et al.*, 2019). The enrichment of longer chain aliphatic waxes, which we observed under WD stress, can result in larger wax crystals, and increased stiffness of the cuticle (reviewed in Heredia-Guerrero *et al.*, 2018) and, on the contrary, wax removal leads to decreases in the elastic modulus and stiffness of fruits (Khanal *et al.*, 2013; Tsubaki *et al.*, 2013). A stiffer cuticle surface, which can also be due to an increase in the wax load (observed in our experiment), can make the fruit more resistant to fungal infections (reviewed in Domínguez *et al.*, 2017). Additionally, increasing stiffness, and thus resistance to tissue expansion (reviewed in Heredia-Guerrero *et al.*, 2018), could be a contributing factor to the berry size reduction under WD. In tomato, quantitative and qualitative changes in the cuticle, that included an increase in cuticular flavonoids and cutin depolymerization, during ripening were related to increases in stiffness and a decrease in extensibility (España *et al.*, 2014*a*, *b*). Additionally, for tomato varieties that increase cuticle wax load and decrease transpiration rates when under drought stress, a direct relationship to increasing fruit firmness has been observed (Romero and Rose, 2019). Based on the relevance of the above parameters for wine and table grape quality, we call for more studies on the impact of cuticular changes on those parameters in grapevine.

### Conclusion

Through phylogenetic and transcriptomic analyses, we identified putative grapevine homologs involved in the biosynthetic pathways for aliphatic cuticular waxes and OA. We demonstrate that normally developing berries experience a decrease in total cuticular wax (aliphatic waxes and triterpenoids combined) load per unit area with berry growth, corresponding to changes in the expression of related biosynthetic genes. During this development, aliphatic cuticular wax content changes substantially, which correlates with a major shift in gene expression centered around the onset of ripening, or veraison. Finally, we determined that genes tentatively associated with aliphatic cuticular wax biosynthesis are transcriptionally up-regulated in grape berries under WD, resulting in increased cuticular wax load with a particularly large increase in wax esters. Yet, this increase did not result in a decrease of the berry’s transpiration rate as expected, suggesting that the detected changes in cuticular wax may serve a different biological purpose for the berry.

## Supplementary data

Supplementary data are available at *JXB* online.

Fig. S1. Schematic of the aliphatic cuticular wax pathway.

Fig. S2. RPKM/FPKM heatmap of putative genes.

Fig. S3. Relative expression of the tissue atlas of the likeliest candidate genes.

Fig. S4. Differential expression heatmap of putative genes.

Fig. S5. Decision tree for selection of the likeliest candidate genes.

Fig. S6. Physiological parameters measured during the water deficit experiment.

Fig. S7. Cuticular VLCFA content of grape berries.

Fig. S8. Cuticular aldehyde content of grape berries.

Fig. S9. Cuticular primary alcohol content of grape berries.

Fig. S10. Cuticular alkane content of grape berries.

Fig. S11. Cuticular triterpenoid content of grape berries.

Fig. S12. SEM images of cuticular wax crystals during berry development.

Fig. S13. Pearson correlation heatmap of gene expression and wax content.

Table S1. Characterized genes involved in cuticular wax biosynthesis and regulation.

Table S2. RNA-seq data sets used for *in silico* analysis.

Table S3. Grapevine tissues used for cDNA library for *in vivo* validation.

Table S4. Primer sequences used for RT–qPCR.

eraa046_suppl_Supplementary_Figures_S1-S13

eraa046_suppl_Supplementary_Tables_S1-S4

## Abbreviations

ABA: abscisic acid
BAS: β-amyrin synthase
CER10: an ECR
CER2: BAHD acyltransferase
CER2-LIKE1: ECERIFERUM2-LIKE1
CER2-LIKE2: ECERIFERUM2-LIKE2
CER4: fatty acyl-CoA reductase
CER6: a KCS
CT: control
DAA: days after anthesis
DE: differential expression
ECR: enoyl-CoA reductase
ER: endoplasmic reticulum
FAAR: fatty acyl-CoA reductase
FAE: fatty acid elongase complex
FPKM: fragments per kilobase of transcript per million mapped reads
HCD: β-hydroxyacyl-CoA dehydratase
KCR: β-ketoacyl-CoA reductase
KCS: ketoacyl-CoA synthase
OA: oleanolic acid
PAS2: an HCD
RPKM: reads per kilobase of transcript per million mapped reads
TF: transcription factor
VLC: very long chain
VLCFA: very long chain fatty acid
WD: water deficit
WSD1: wax ester synthase/acyl-CoA:diacylglycerol acyltransferase

## References

[CIT0001] Agudelo-Romero P, Erban A, Rego C, Carbonell-Bejerano P, Nascimento T, Sousa L, Martínez-Zapater JM, Kopka J, Fortes AM 2015 Transcriptome and metabolome reprogramming in *Vitis vinifera* cv. Trincadeira berries upon infection with *Botrytis cinerea*. Journal of Experimental Botany 66, 1769–1785. 25675955 10.1093/jxb/eru517 PMC4669548

[CIT0002] Bach L, Michaelson LV, Haslam R, et al 2008 The very-long-chain hydroxy fatty acyl-CoA dehydratase PASTICCINO2 is essential and limiting for plant development. Proceedings of the National Academy of Sciences, USA 105, 14727–14731. 10.1073/pnas.0805089105 PMC2567193 18799749

[CIT0003] Beaudoin F, Wu X, Li F, Haslam RP, Markham JE, Zheng H, Napier JA, Kunst L 2009 Functional characterization of the Arabidopsis beta-ketoacyl-coenzyme A reductase candidates of the fatty acid elongase. Plant Physiology 150, 1174–1191. 19439572 10.1104/pp.109.137497 PMC2705042

[CIT0004] Bernard A, Domergue F, Pascal S, Jetter R, Renne C, Faure JD, Haslam RP, Napier JA, Lessire R, Joubès J 2012 Reconstitution of plant alkane biosynthesis in yeast demonstrates that Arabidopsis ECERIFERUM1 and ECERIFERUM3 are core components of a very-long-chain alkane synthesis complex. The Plant Cell 24, 3106–3118. 22773744 10.1105/tpc.112.099796 PMC3426135

[CIT0005] Bernard A, Joubès J 2013 Arabidopsis cuticular waxes: advances in synthesis, export and regulation. Progress in Lipid Research 52, 110–129. 23103356 10.1016/j.plipres.2012.10.002

[CIT0006] Bi H, Kovalchuk N, Langridge P, Tricker PJ, Lopato S, Borisjuk N 2017 The impact of drought on wheat leaf cuticle properties. BMC Plant Biology 17, 1–13. 28482800 10.1186/s12870-017-1033-3 PMC5422891

[CIT0007] Bolger AM, Lohse M, Usadel B 2014 Trimmomatic: a flexible trimmer for Illumina sequence data. Bioinformatics 30, 2114–2120. 24695404 10.1093/bioinformatics/btu170 PMC4103590

[CIT0008] Bourdenx B, Bernard A, Domergue F, et al 2011 Overexpression of Arabidopsis ECERIFERUM1 promotes wax very-long-chain alkane biosynthesis and influences plant response to biotic and abiotic stresses. Plant Physiology 156, 29–45. 21386033 10.1104/pp.111.172320 PMC3091054

[CIT0009] Camacho C, Coulouris G, Avagyan V, Ma N, Papadopoulos J, Bealer K, Madden TL 2009 BLAST+: architecture and applications. BMC Bioinformatics 10, 421. 20003500 10.1186/1471-2105-10-421 PMC2803857

[CIT0010] Cameron KD, Teece MA, Smart LB 2006 Increased accumulation of cuticular wax and expression of lipid transfer protein in response to periodic drying events in leaves of tree tobacco. Plant Physiology 140, 176–183. 16361524 10.1104/pp.105.069724 PMC1326042

[CIT0011] Campagna D, Albiero A, Bilardi A, Caniato E, Forcato C, Manavski S, Vitulo N, Valle G 2009 PASS: a program to align short sequences. Bioinformatics 25, 967–968. 19218350 10.1093/bioinformatics/btp087

[CIT0012] Casado CG, Heredia A 1999 Structure and dynamics of reconstituted cuticular waxes of grape berry cuticle (*Vitis vinifera* L.). Journal of Experimental Botany 50, 175–182.

[CIT0013] Castellarin SD, Matthews MA, Di Gaspero G, Gambetta GA 2007 *a* Water deficits accelerate ripening and induce changes in gene expression regulating flavonoid biosynthesis in grape berries. Planta 227, 101–112. 17694320 10.1007/s00425-007-0598-8

[CIT0014] Castellarin SD, Pfeiffer A, Sivilotti P, Degan M, Peterlunger E, Di Gaspero G 2007 *b* Transcriptional regulation of anthocyanin biosynthesis in ripening fruits of grapevine under seasonal water deficit. Plant, Cell & Environment 30, 1381–1399. 10.1111/j.1365-3040.2007.01716.x 17897409

[CIT0015] Charrier G, Delzon S, Domec JC, et al 2018 Drought will not leave your glass empty: low risk of hydraulic failure revealed by long-term drought observations in world’s top wine regions. Science Advances 4, eaao6969. 29404405 10.1126/sciadv.aao6969 PMC5796794

[CIT0016] Chevenet F, Brun C, Bañuls AL, Jacq B, Christen R 2006 TreeDyn: towards dynamic graphics and annotations for analyses of trees. BMC Bioinformatics 7, 439. 17032440 10.1186/1471-2105-7-439 PMC1615880

[CIT0017] Commenil P, Brunet L, Audran J 1997 The development of the grape berry cuticle in relation to susceptibility to bunch rot disease. Journal of Experimental Botany 48, 1599–1607.

[CIT0018] Cui F, Brosché M, Lehtonen MT, Amiryousefi A, Xu E, Punkkinen M, Valkonen JP, Fujii H, Overmyer K 2016 Dissecting abscisic acid signaling pathways involved in cuticle formation. Molecular Plant 9, 926–938. 27060495 10.1016/j.molp.2016.04.001

[CIT0019] Da Silva C, Zamperin G, Ferrarini A, et al 2013 The high polyphenol content of grapevine cultivar tannat berries is conferred primarily by genes that are not shared with the reference genome. The Plant Cell 25, 4777–4788. 24319081 10.1105/tpc.113.118810 PMC3903987

[CIT0020] Dereeper A, Guignon V, Blanc G, et al 2008 Phylogeny.fr: robust phylogenetic analysis for the non-specialist. Nucleic Acids Research 36, W465–W469. 18424797 10.1093/nar/gkn180 PMC2447785

[CIT0021] Domínguez E, España L, López-Casado G, Cuartero J, Heredia A 2009 Biomechanics of isolated tomato (*Solanum lycopersicum*) fruit cuticles during ripening: the role of flavonoids. Functional Plant Biology 36, 613–620. 10.1071/FP09039 32688674

[CIT0022] Domínguez E, Heredia-Guerrero JA, Heredia A 2017 The plant cuticle: old challenges, new perspectives. Journal of Experimental Botany 68, 5251–5255. 29136457 10.1093/jxb/erx389 PMC5853762

[CIT0023] España L, Heredia-Guerrero JA, Reina-Pinto JJ, Fernández-Muñoz R, Heredia A, Domínguez E 2014 *a* Transient silencing of CHALCONE SYNTHASE during fruit ripening modifies tomato epidermal cells and cuticle properties. Plant Physiology 166, 1371–1386. 25277718 10.1104/pp.114.246405 PMC4226350

[CIT0024] España L, Heredia-Guerrero JA, Segado P, Benítez JJ, Heredia A, Domínguez E 2014 *b* Biomechanical properties of the tomato (*Solanum lycopersicum*) fruit cuticle during development are modulated by changes in the relative amounts of its components. New Phytologist 202, 790–802. 24571168 10.1111/nph.12727

[CIT0025] Fiebig A, Mayfield JA, Miley NL, Chau S, Fischer RL, Preuss D 2000 Alterations in *CER6*, a gene identical to *CUT1*, differentially affect long-chain lipid content on the surface of pollen and stems. The Plant Cell 12, 2001–2008. 11041893 10.1105/tpc.12.10.2001 PMC149136

[CIT0026] Fukushima EO, Seki H, Ohyama K, Ono E, Umemoto N, Mizutani M, Saito K, Muranaka T 2011 CYP716A subfamily members are multifunctional oxidases in triterpenoid biosynthesis. Plant & Cell Physiology 52, 2050–2061. 22039103 10.1093/pcp/pcr146

[CIT0027] Girard AL, Mounet F, Lemaire-Chamley M, et al 2012 Tomato GDSL1 is required for cutin deposition in the fruit cuticle. The Plant Cell 24, 3119–3134. 22805434 10.1105/tpc.112.101055 PMC3426136

[CIT0028] Go YS, Kim H, Kim HJ, Suh MC 2014 Arabidopsis cuticular wax biosynthesis is negatively regulated by the *DEWAX* gene encoding an AP2/ERF-type transcription factor. The Plant Cell 26, 1666–1680. 24692420 10.1105/tpc.114.123307 PMC4036578

[CIT0029] Grncarevic M, Radler F 1971 A review of the surface lipids of grapes and their importance in the drying process. American Journal of Enology and Viticulture 22, 80–86.

[CIT0030] Guindon S, Dufayard JF, Lefort V, Anisimova M, Hordijk W, Gascuel O 2010 New algorithms and methods to estimate maximum-likelihood phylogenies: assessing the performance of PhyML 3.0. Systematic Biology 59, 307–321. 20525638 10.1093/sysbio/syq010

[CIT0031] Haslam TM, Haslam R, Thoraval D, et al 2015 ECERIFERUM2-LIKE proteins have unique biochemical and physiological functions in very-long-chain fatty acid elongation. Plant Physiology 167, 682–692. 25596184 10.1104/pp.114.253195 PMC4348766

[CIT0032] Haslam TM, Kunst L 2013 *a* Extending the story of very-long-chain fatty acid elongation. Plant Science 210, 93–107. 23849117 10.1016/j.plantsci.2013.05.008

[CIT0033] Haslam TM, Kunst L 2013 *b* Wax analysis of stem and rosette leaves in *Arabidopsis thaliana*. Bioprotocols 3, e782.

[CIT0034] Haslam TM, Mañas-Fernández A, Zhao L, Kunst L 2012 Arabidopsis ECERIFERUM2 is a component of the fatty acid elongation machinery required for fatty acid extension to exceptional lengths. Plant Physiology 160, 1164–1174. 22930748 10.1104/pp.112.201640 PMC3490600

[CIT0035] Heredia-Guerrero JA, Guzman-Puyol S, Benítez JJ, Athanassiou A, Heredia A, Domínguez E 2018 Plant cuticle under global change: biophysical implications. Global Change Biology 24, 2749–2751. 29668107 10.1111/gcb.14276

[CIT0036] Hochberg U, Degu A, Fait A, Rachmilevitch S 2013 *a* Near isohydric grapevine cultivar displays higher photosynthetic efficiency and photorespiration rates under drought stress as compared with near anisohydric grapevine cultivar. Physiologia Plantarum 147, 443–452. 22901023 10.1111/j.1399-3054.2012.01671.x

[CIT0037] Hochberg U, Degu A, Toubiana D, Gendler T, Nikoloski Z, Rachmilevitch S, Fait A 2013 *b* Metabolite profiling and network analysis reveal coordinated changes in grapevine water stress response. BMC Plant Biology 13, 184. 24256338 10.1186/1471-2229-13-184 PMC4225576

[CIT0038] Holmes MG, Keiller DR 2002 Effects of pubescence and waxes on the reflectance of leaves in the ultraviolet and photosynthetic wavebands: a comparison of a range of species. Plant, Cell & Environment 25, 85–93.

[CIT0039] Huang H, Burghardt M, Schuster AC, Leide J, Lara I, Riederer M 2017 Chemical composition and water permeability of fruit and leaf cuticles of *Olea europaea* L. Journal of Agricultural and Food Chemistry 65, 8790–8797. 28880084 10.1021/acs.jafc.7b03049

[CIT0040] Isaacson T, Kosma DK, Matas AJ, et al 2009 Cutin deficiency in the tomato fruit cuticle consistently affects resistance to microbial infection and biomechanical properties, but not transpirational water loss. The Plant Journal 60, 363–377. 19594708 10.1111/j.1365-313X.2009.03969.x

[CIT0041] Jaillon O, Aury JM, Noel B, et al 2007 The grapevine genome sequence suggests ancestral hexaploidization in major angiosperm phyla. Nature 449, 463–467. 17721507 10.1038/nature06148

[CIT0042] Jetter R, Riederer M 2016 Localization of the transpiration barrier in the epi- and intracuticular waxes of eight plant species: water transport resistances are associated with fatty acyl rather than alicyclic components. Plant Physiology 170, 921–934. 26644508 10.1104/pp.15.01699 PMC4734581

[CIT0043] Kachroo A, Robin GP 2013 Systemic signaling during plant defense. Current Opinion in Plant Biology 16, 527–533. 23870750 10.1016/j.pbi.2013.06.019

[CIT0044] Khanal BP, Grimm E, Finger S, Blume A, Knoche M 2013 Intracuticular wax fixes and restricts strain in leaf and fruit cuticles. New Phytologist 200, 134–143. 23750808 10.1111/nph.12355

[CIT0045] Kim D, Langmead B, Salzberg SL 2015 HISAT: a fast spliced aligner with low memory requirements. Nature Methods 12, 357–360. 25751142 10.1038/nmeth.3317 PMC4655817

[CIT0046] Kim KS, Park SH, Jenks MA 2007 Changes in leaf cuticular waxes of sesame (*Sesamum indicum* L.) plants exposed to water deficit. Journal of Plant Physiology 164, 1134–1143. 16904233 10.1016/j.jplph.2006.07.004

[CIT0047] Kosma DK, Bourdenx B, Bernard A, Parsons EP, Lü S, Joubès J, Jenks MA 2009 The impact of water deficiency on leaf cuticle lipids of Arabidopsis. Plant Physiology 151, 1918–1929. 19819982 10.1104/pp.109.141911 PMC2785987

[CIT0048] Lacoste F 1865 Guide pratique du vigneron: culture, vendange et vinification. Paris: Eugene Lacroix.

[CIT0049] Lara I, Belge B, Goulao LF 2015 A focus on the biosynthesis and composition of cuticle in fruits. Journal of Agricultural and Food Chemistry 63, 4005–4019. 25850334 10.1021/acs.jafc.5b00013

[CIT0050] Lara I, Heredia A, Domínguez E 2019 Shelf life potential and the fruit cuticle: the unexpected player. Frontiers in Plant Science 10, 770. 31244879 10.3389/fpls.2019.00770 PMC6581714

[CIT0051] Lee SB, Kim HU, Suh MC 2016 MYB94 and MYB96 additively activate cuticular wax biosynthesis in Arabidopsis. Plant & Cell Physiology 57, 2300–2311. 27577115 10.1093/pcp/pcw147

[CIT0052] Leida C, Dal Rì A, Dalla Costa L, Gómez MD, Pompili V, Sonego P, Engelen K, Masuero D, Ríos G, Moser C 2016 Insights into the role of the berry-specific ethylene responsive factor *VviERF045*. Frontiers in Plant Science 7, 1793. 28018369 10.3389/fpls.2016.01793 PMC5146979

[CIT0053] Leide J, Hildebrandt U, Reussing K, Riederer M, Vogg G 2007 The developmental pattern of tomato fruit wax accumulation and its impact on cuticular transpiration barrier properties: effects of a deficiency in a beta-ketoacyl-coenzyme A synthase (LeCER6). Plant Physiology 144, 1667–1679. 17468214 10.1104/pp.107.099481 PMC1914139

[CIT0054] Levin AD, Williams LE, Matthews MA 2019 A continuum of stomatal responses to water deficits among 17 wine grape cultivars (*Vitis vinifera*). Functional Plant Biology 11–25. 31615618 10.1071/FP19073

[CIT0055] Li F, Wu X, Lam P, Bird D, Zheng H, Samuels L, Jetter R, Kunst L 2008 Identification of the wax ester synthase/acyl-coenzyme A:diacylglycerol acyltransferase WSD1 required for stem wax ester biosynthesis in Arabidopsis. Plant Physiology 148, 97–107. 18621978 10.1104/pp.108.123471 PMC2528131

[CIT0056] Liao Y, Smyth GK, Shi W 2014 featureCounts: an efficient general purpose program for assigning sequence reads to genomic features. Bioinformatics 30, 923–930. 24227677 10.1093/bioinformatics/btt656

[CIT0057] López-Castañeda J, Corrales-García J, Terrazas-Salgado T, Colinas-León T 2010 Effect of vapor heat treatments on weight loss reduction and epicuticular changes in six varieties of cactus pear fruit (*Opuntia* spp.). Journal of the Professional Association for Cactus Development 12, 37–47.

[CIT0058] Lovisolo C, Lavoie-Lamoureux A, Tramontini S, Ferrandino A 2016 Grapevine adaptations to water stress: new perspectives about soil/plant interactions. Theoretical and Experimental Plant Physiology 28, 53–66.

[CIT0059] Lun ATL, Chen Y, Smyth GK 2016 It’s DE-licious: a recipe for differential expression analyses of RNA-seq experiments using quasi-likelihood methods in edgeR. In: Mathé E, Davis S, eds. Statistical genomics: methods and protocols. New York: Springer Science+Business Media, 391–416. 10.1007/978-1-4939-3578-9_19 27008025

[CIT0060] Luque P, Bruque S, Heredia A 1995 Water permeability of isolated cuticular membranes: a structural analysis. Archives of Biochemistry and Biophysics 317, 417–422. 7893158 10.1006/abbi.1995.1183

[CIT0061] Martin LB, Rose JK 2014 There’s more than one way to skin a fruit: formation and functions of fruit cuticles. Journal of Experimental Botany 65, 4639–4651. 25028557 10.1093/jxb/eru301

[CIT0062] Oshima Y, Shikata M, Koyama T, Ohtsubo N, Mitsuda N, Ohme-Takagi M 2013 MIXTA-like transcription factors and WAX INDUCER1/SHINE1 coordinately regulate cuticle development in *Arabidopsis* and *Torenia fournieri*. The Plant Cell 25, 1609–1624. 23709630 10.1105/tpc.113.110783 PMC3694695

[CIT0063] Palliotti A, Cartechini A 2001 Developmental changes in gas exchange activity in flowers, berries, and tendrils of field-grown Cabernet Sauvignon. American Journal of Enology and Viticulture 52, 317–323.

[CIT0064] Palumbo MC, Zenoni S, Fasoli M, Massonnet M, Farina L, Castiglione F, Pezzotti M, Paci P 2014 Integrated network analysis identifies fight-club nodes as a class of hubs encompassing key putative switch genes that induce major transcriptome reprogramming during grapevine development. The Plant Cell 26, 4617–4635. 25490918 10.1105/tpc.114.133710 PMC4311215

[CIT0065] Parsons EP, Popopvsky S, Lohrey GT, Alkalai-Tuvia S, Perzelan Y, Bosland P, Bebeli PJ, Paran I, Fallik E, Jenks MA 2013 Fruit cuticle lipid composition and water loss in a diverse collection of pepper (*Capsicum*). Physiologia Plantarum 149, 160–174. 23496056 10.1111/ppl.12035

[CIT0066] Patwari P, Salewski V, Gutbrod K, Kreszies T, Dresen-Scholz B, Peisker H, Steiner U, Meyer AJ, Schreiber L, Dörmann P 2019 Surface wax esters contribute to drought tolerance in Arabidopsis. The Plant Journal 98, 727–744. 30729606 10.1111/tpj.14269

[CIT0067] Pensec F, Pączkowski C, Grabarczyk M, Woźniak A, Bénard-Gellon M, Bertsch C, Chong J, Szakiel A 2014 Changes in the triterpenoid content of cuticular waxes during fruit ripening of eight grape (*Vitis vinifera*) cultivars grown in the Upper Rhine Valley. Journal of Agricultural and Food Chemistry 62, 7998–8007. 25058466 10.1021/jf502033s

[CIT0068] Petit J, Bres C, Mauxion JP, Bakan B, Rothan C 2017 Breeding for cuticle-associated traits in crop species: traits, targets, and strategies. Journal of Experimental Botany 68, 5369–5387. 29036305 10.1093/jxb/erx341

[CIT0069] R Core Team 2019 R: a language and environment for statistical computing. Vienna, Austria: R Foundation for Statistical Computing.

[CIT0070] Radler F 1965 The main constituents of the surface waxes of varieties and species of the genus *Vitis*. American Journal of Enology and Viticulture 16, 159–167.

[CIT0071] Rogiers SY, Greer DH, Hutton RJ, Landsberg JJ 2009 Does night-time transpiration contribute to anisohydric behaviour in a *Vitis vinifera* cultivar? Journal of Experimental Botany 60, 3751–3763. 19584116 10.1093/jxb/erp217 PMC2736890

[CIT0072] Rogiers SY, Hatfield JM, Gunta Jaudzems V, White RG, Keller M 2004 Grape berry cv. Shiraz epicuticular wax and transpiration during ripening and preharvest weight loss. American Journal of Enology and Viticulture 55, 121–127.

[CIT0073] Romero P, Rose JKC 2019 A relationship between tomato fruit softening, cuticle properties and water availability. Food Chemistry 295, 300–310. 31174762 10.1016/j.foodchem.2019.05.118

[CIT0074] Rowland O, Zheng H, Hepworth SR, Lam P, Jetter R, Kunst L 2006 *CER4* encodes an alcohol-forming fatty acyl-coenzyme A reductase involved in cuticular wax production in Arabidopsis. Plant Physiology 142, 866–877. 16980563 10.1104/pp.106.086785 PMC1630741

[CIT0075] Savoi S, Wong DCJ, Degu A, Herrera JC, Bucchetti B, Peterlunger E, Fait A, Mattivi F, Castellarin SD 2017 Multi-omics and integrated network analyses reveal new insights into the systems relationships between metabolites, structural genes, and transcriptional regulators in developing grape berries (*Vitis vinifera* L.) exposed to water deficit. Frontiers in Plant Science 8, 1124. 28740499 10.3389/fpls.2017.01124 PMC5502274

[CIT0076] Scharwies JD, Tyerman SD 2017 Comparison of isohydric and anisohydric *Vitis vinifera* L. cultivars reveals a fine balance between hydraulic resistances, driving forces and transpiration in ripening berries. Functional Plant Biology 44, 324–338. 10.1071/FP16010 32480567

[CIT0077] Seo PJ, Lee SB, Suh MC, Park MJ, Go YS, Park CM 2011 The MYB96 transcription factor regulates cuticular wax biosynthesis under drought conditions in *Arabidopsis*. The Plant Cell 23, 1138–1152. 21398568 10.1105/tpc.111.083485 PMC3082259

[CIT0078] Talavera G, Castresana J 2007 Improvement of phylogenies after removing divergent and ambiguously aligned blocks from protein sequence alignments. Systematic Biology 56, 564–577. 17654362 10.1080/10635150701472164

[CIT0079] Tsubaki S, Sugimura K, Teramoto Y, Yonemori K, Azuma J 2013 Cuticular membrane of *Fuyu* persimmon fruit is strengthened by triterpenoid nano-fillers. PLoS One 8, e75275. 24086493 10.1371/journal.pone.0075275 PMC3782500

[CIT0080] Vitulo N, Forcato C, Carpinelli EC, et al 2014 A deep survey of alternative splicing in grape reveals changes in the splicing machinery related to tissue, stress condition and genotype. BMC Plant Biology 14, 99. 24739459 10.1186/1471-2229-14-99 PMC4108029

[CIT0081] Vogg G, Fischer S, Leide J, Emmanuel E, Jetter R, Levy AA, Riederer M 2004 Tomato fruit cuticular waxes and their effects on transpiration barrier properties: functional characterization of a mutant deficient in a very-long-chain fatty acid beta-ketoacyl-CoA synthase. Journal of Experimental Botany 55, 1401–1410. 15133057 10.1093/jxb/erh149

[CIT0082] Wong DC, Lopez Gutierrez R, Dimopoulos N, Gambetta GA, Castellarin SD 2016 Combined physiological, transcriptome, and cis-regulatory element analyses indicate that key aspects of ripening, metabolism, and transcriptional program in grapes (*Vitis vinifera* L.) are differentially modulated accordingly to fruit size. BMC Genomics 17, 416. 27245662 10.1186/s12864-016-2660-z PMC4886440

[CIT0083] Xu X, Xiao L, Feng J, Chen N, Chen Y, Song B, Xue K, Shi S, Zhou Y, Jenks MA 2016 Cuticle lipids on heteromorphic leaves of *Populus euphratica* Oliv. growing in riparian habitats differing in available soil moisture. Physiologia Plantarum 158, 318–330. 27184005 10.1111/ppl.12471

[CIT0084] Yeats TH, Rose JK 2013 The formation and function of plant cuticles. Plant Physiology 163, 5–20. 23893170 10.1104/pp.113.222737 PMC3762664

[CIT0085] Zhang Y, Keller M 2015 Grape berry transpiration is determined by vapor pressure deficit, cuticular conductance, and berry size. American Journal of Enology and Viticulture 66, 454–462.

[CIT0086] Zheng H, Rowland O, Kunst L 2005 Disruptions of the Arabidopsis enoyl-CoA reductase gene reveal an essential role for very-long-chain fatty acid synthesis in cell expansion during plant morphogenesis. The Plant Cell 17, 1467–1481. 15829606 10.1105/tpc.104.030155 PMC1091768

