# Supplementary material for: Drought stress modulates cuticular wax composition of the grape berry
Source: J Exp Bot. 2020 Jan 27;71(10):3126–41. doi: 10.1093/jxb/eraa046 (PMC7260727; doi:10.1093/jxb/eraa046)
Supplement: eraa046_suppl_Supplementary_Figures_S1-S13 [file eraa046_suppl_supplementary_figures_s1-s13.pdf]

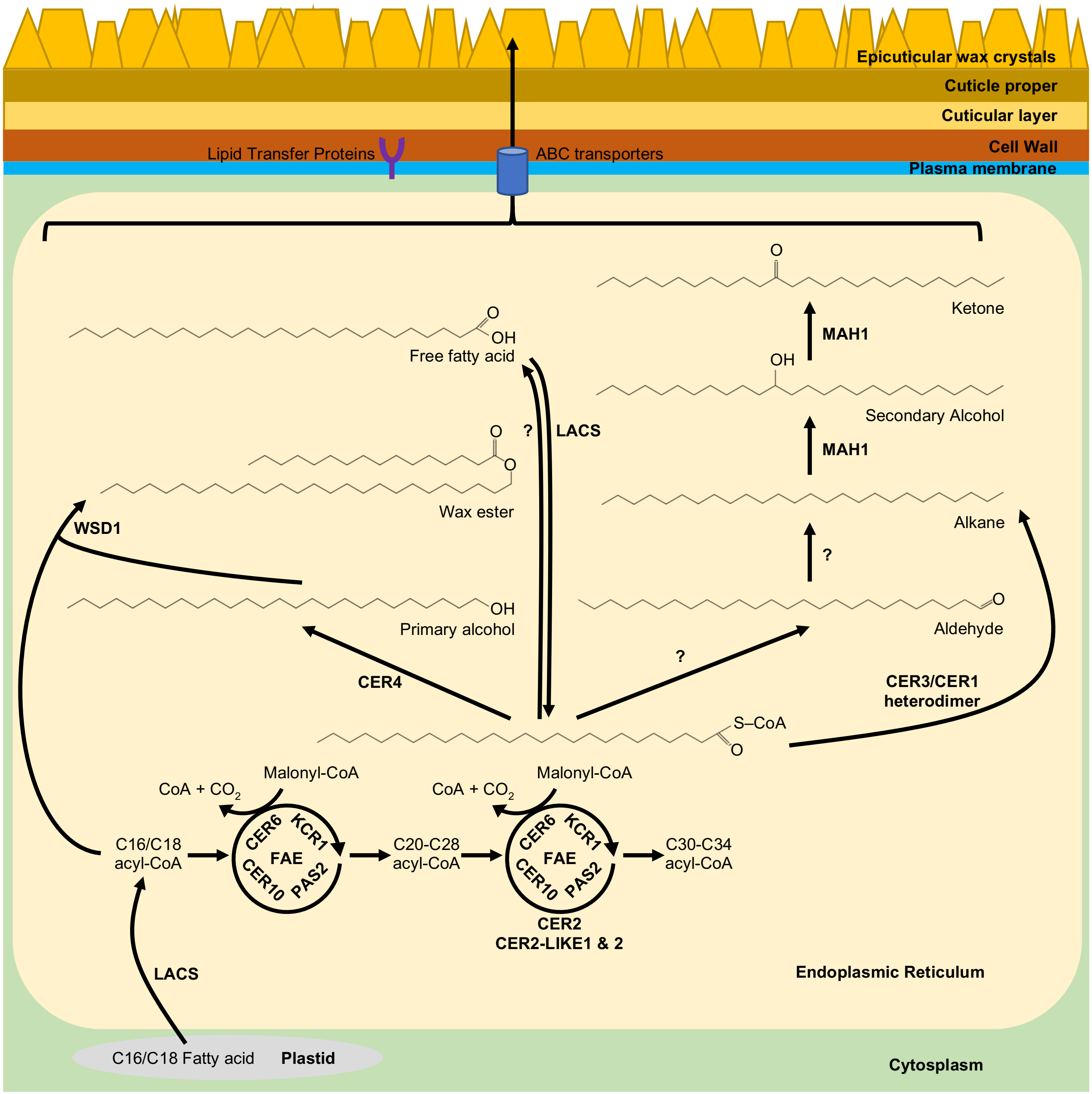

Figure S1. The cuticular aliphatic wax biosynthetic pathway. The fatty acid elongase (FAE) complex is responsible for synthesis of very long chain (VLC)-fatty acids; proteins involved are CER6, KCR1, PAS2, CER10, CER2, CER2-LIKE1, CER2-LIKE2. The alcohol forming branch includes CER4 and WSD1 proteins that produce VLC-primary alcohols and wax esters. The alkane forming branch includes CER1, CER3, and MAH1 proteins that produce VLC-alkanes, VLC-aldehydes, VLC-secondary alcohols, and VLC-ketones. Alipatic waxes are exported to the cuticle by way of ABC transporters and lipid transfer proteins.

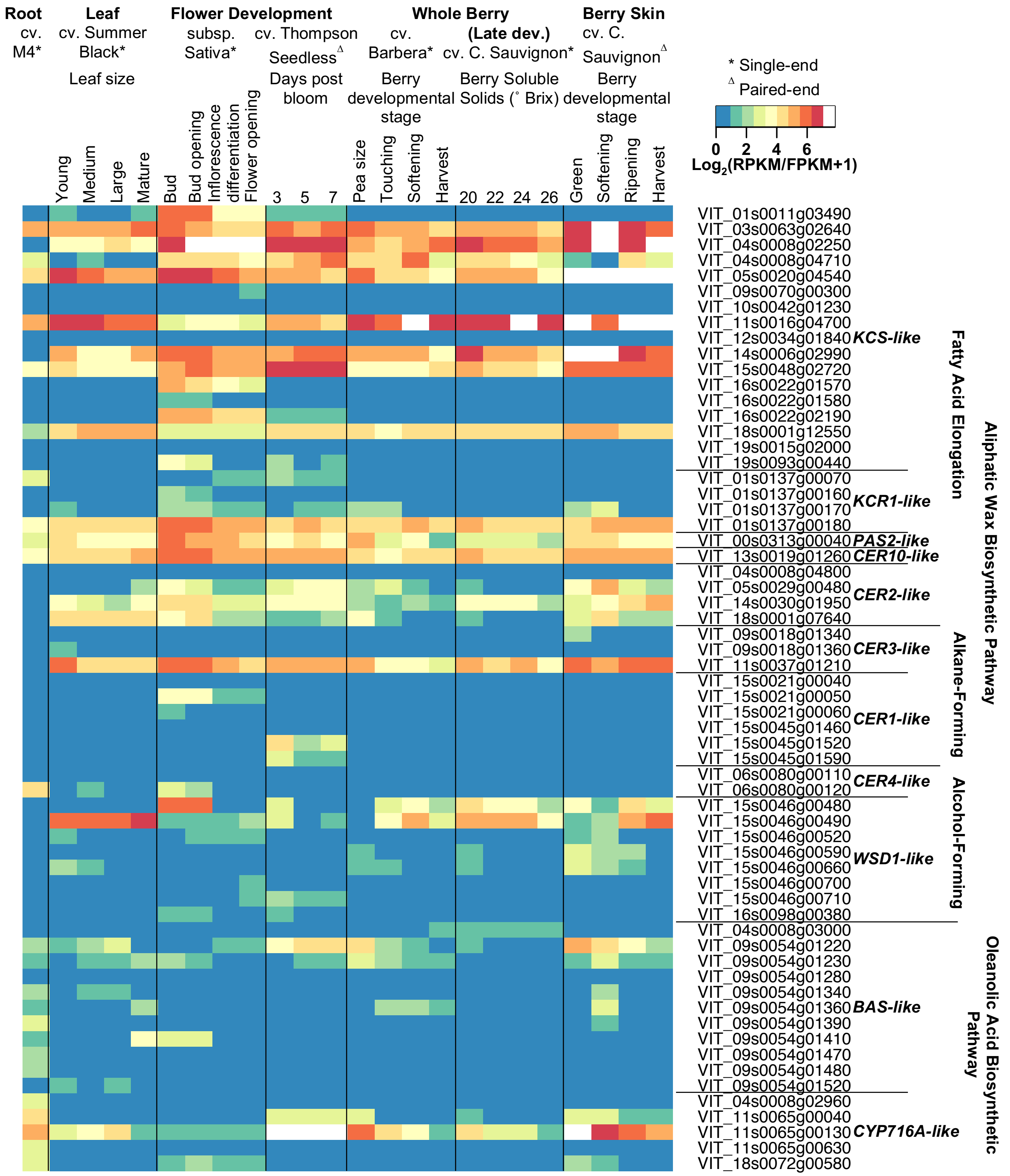

Figure S2. Heatmap of the relative expression in terms of $\log_2$(rpkm+1) or $\log_2$(fpkm+1) of grapevine (*Vitis vinifera* L.) putative homologs involved in cuticular aliphatic wax and oleanolic acid biosynthesis. RNA-seq datasets were retrieved from the DNA data bank of Japan and reprocessed. The datasets cover development in root, leaf, flower, inflorescence, whole berry, and berry skin tissues.

Figure S3. Expression of wax related genes in various grapevine (*Vitis vinifera* L) var. Gewürztraminer, tissues. Plants were grown under optimal, well watered greenhouse conditions. Error bars represent ± S.E . Genes involved in fatty acid elongation **(A-I)**, alkane-forming branch **(J-L)**, alcohol-forming branch **(M-P)**, oleanolic acid synthesis **(Q-R)**, and cuticle synthesis regulation **(S-X)** are shown.

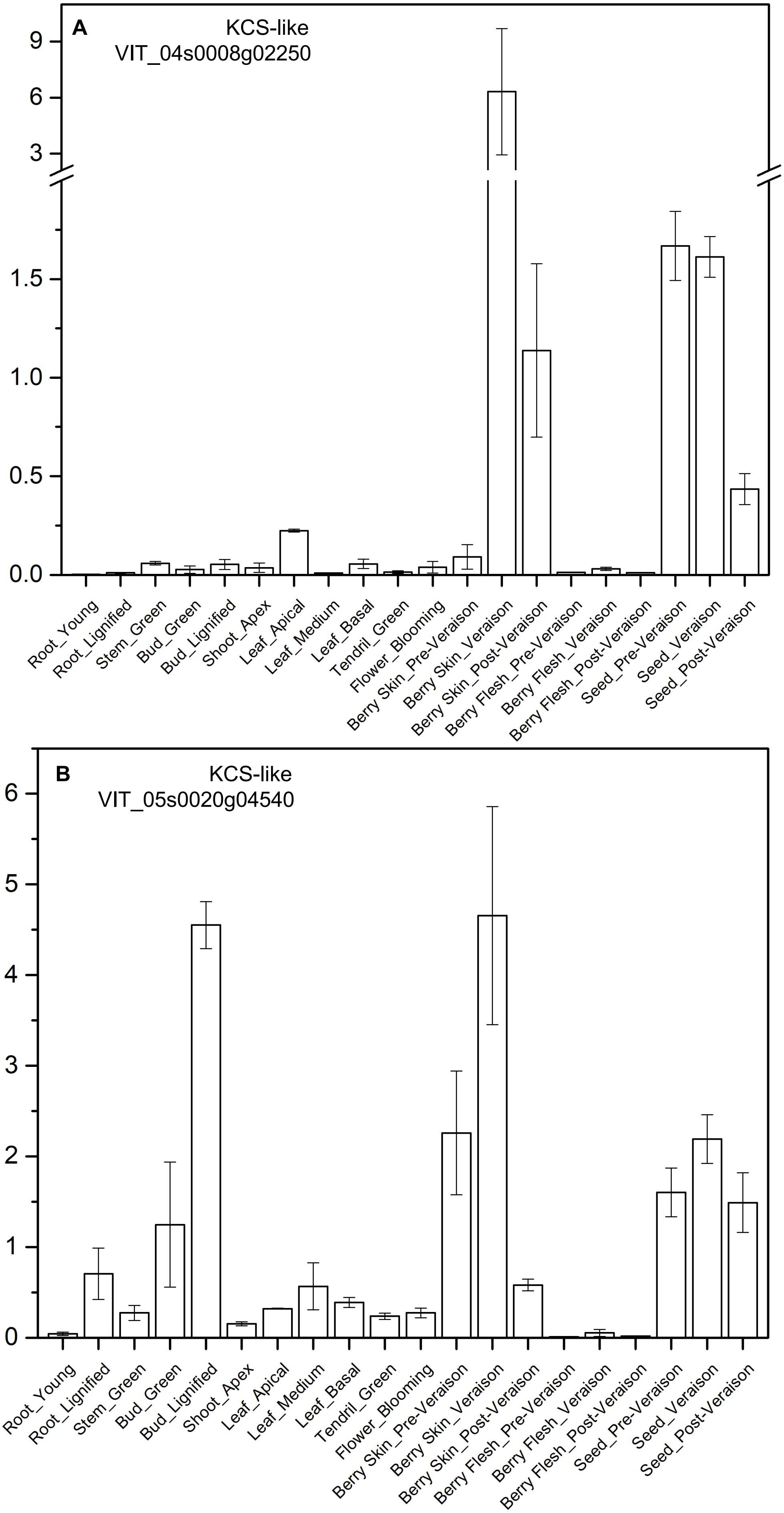

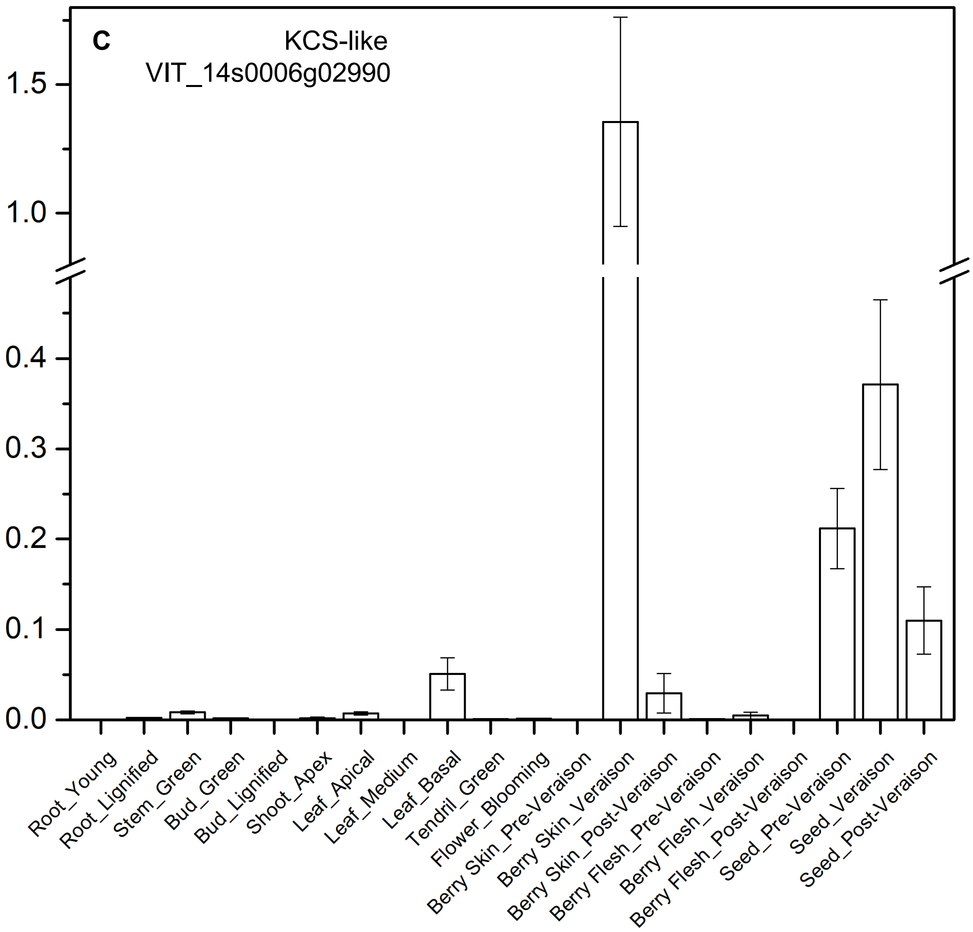
C
KCS-like
VIT_14s0006g02990
1.5
1.0
0.4
0.3
0.2
0.1
0.0
Root_Young
Root_Lignified
Stem_Green
Bud_Green
Bud_Lignified
Shoot_Apex
Leaf_Apical
Leaf_Medium
Leaf_Basal
Tendril_Green
Flower_Blooming
Berry Skin_Pre-Veraison
Berry Skin_Veraison
Berry Skin_Post-Veraison
Berry Flesh_Pre-Veraison
Berry Flesh_Veraison
Berry Flesh_Post-Veraison
Seed_Pre-Veraison
Seed_Veraison
Seed_Post-Veraison

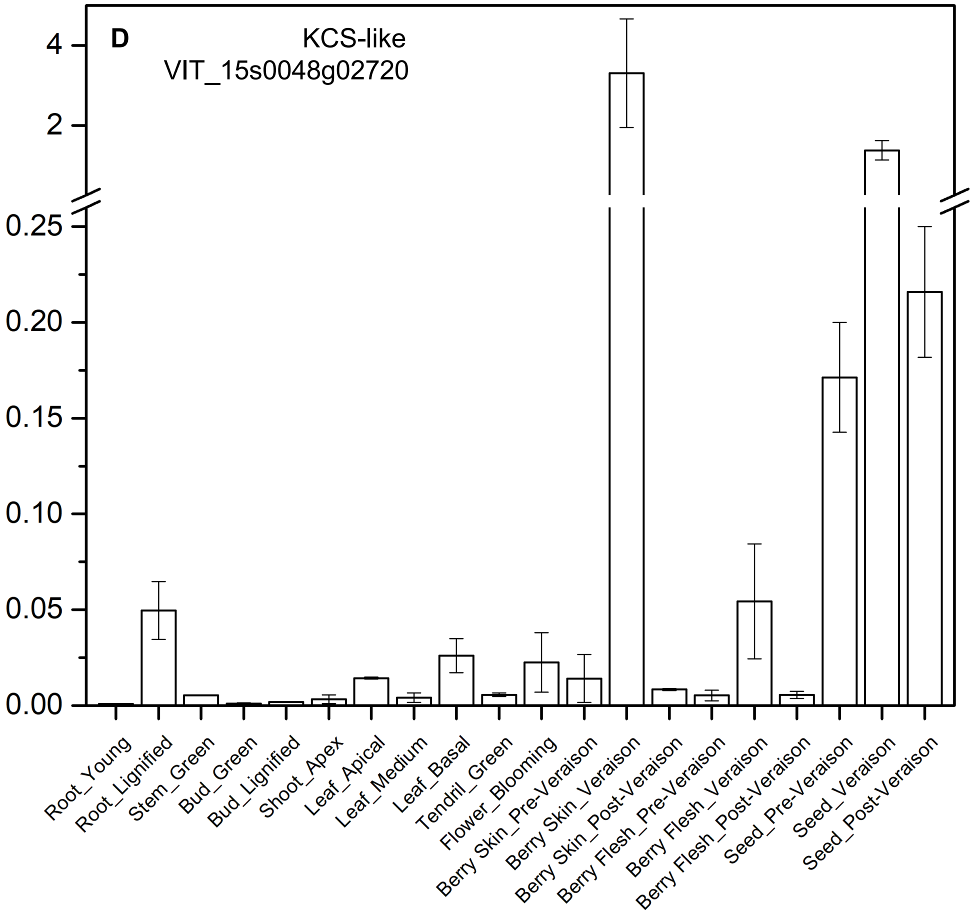
D
KCS-like
VIT_15s0048g02720
4
2
0.25
0.20
0.15
0.10
0.05
0.00
Root_Young
Root_Lignified
Stem_Green
Bud_Green
Bud_Lignified
Shoot_Apex
Leaf_Apical
Leaf_Medium
Leaf_Basal
Tendril_Green
Flower_Blooming
Berry Skin_Pre-Veraison
Berry Skin_Veraison
Berry Skin_Post-Veraison
Berry Flesh_Pre-Veraison
Berry Flesh_Veraison
Berry Flesh_Post-Veraison
Seed_Pre-Veraison
Seed_Veraison
Seed_Post-Veraison

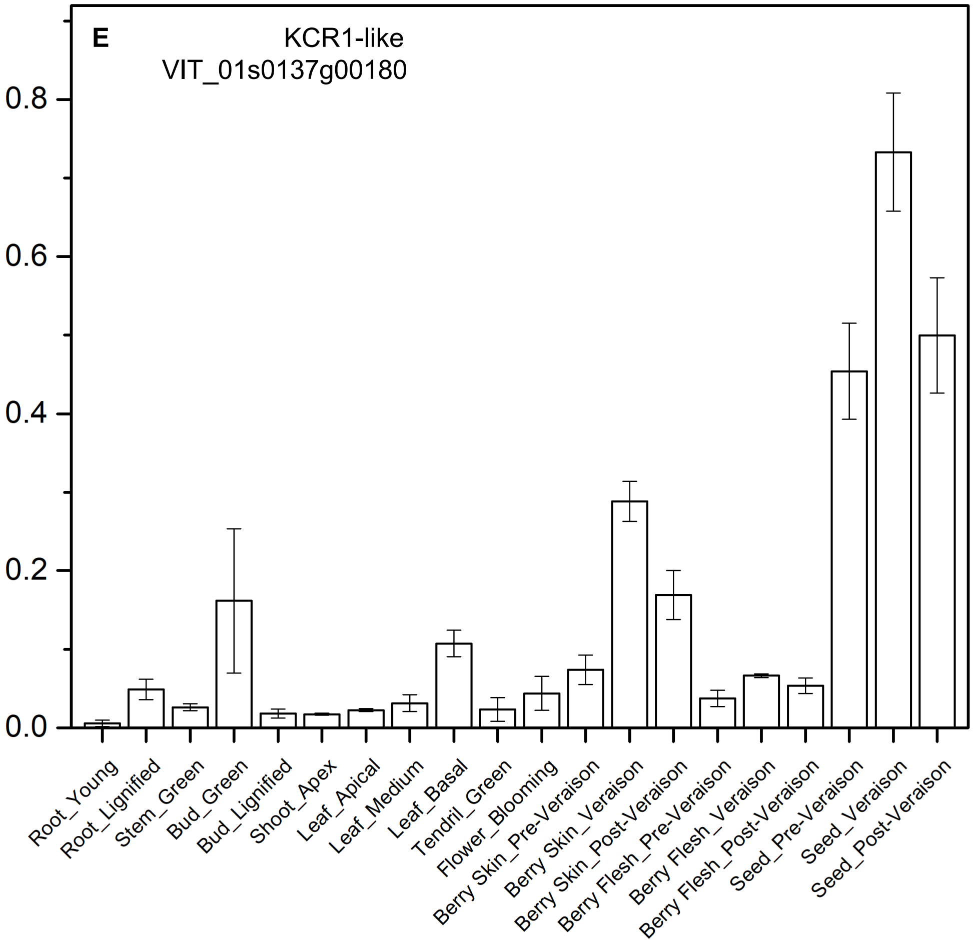
E
KCR1-like
VIT_01s0137g00180
0.0
0.2
0.4
0.6
0.8
Root_Young
Root_Lignified
Stem_Green
Bud_Green
Bud_Lignified
Shoot_Apex
Leaf_Apical
Leaf_Medium
Leaf_Basal
Tendril_Green
Flower_Blooming
Berry Skin_Pre-Veraison
Berry Skin_Veraison
Berry Skin_Post-Veraison
Berry Flesh_Pre-Veraison
Berry Flesh_Veraison
Berry Flesh_Post-Veraison
Seed_Pre-Veraison
Seed_Veraison
Seed_Post-Veraison

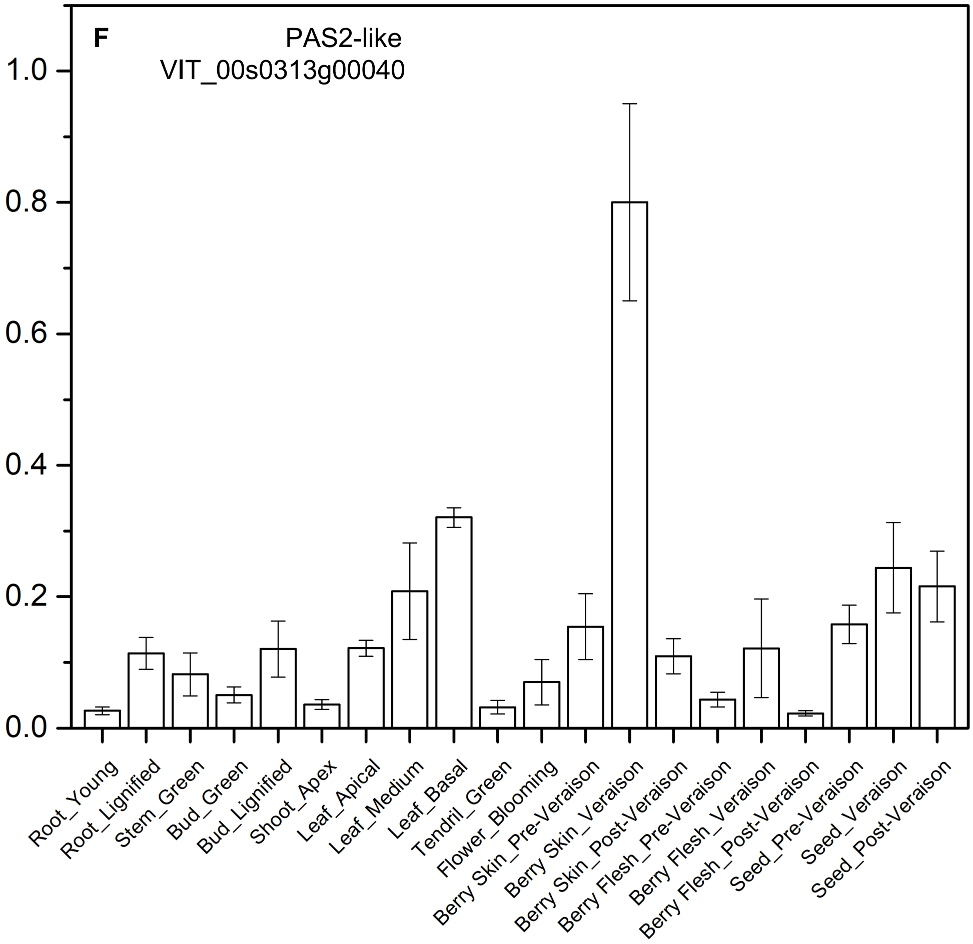
F
PAS2-like
VIT_00s0313g00040
0.0
0.2
0.4
0.6
0.8
1.0
Root_Young
Root_Lignified
Stem_Green
Bud_Green
Bud_Lignified
Shoot_Apex
Leaf_Apical
Leaf_Medium
Leaf_Basal
Tendril_Green
Flower_Blooming
Berry Skin_Pre-Veraison
Berry Skin_Veraison
Berry Skin_Post-Veraison
Berry Flesh_Pre-Veraison
Berry Flesh_Veraison
Berry Flesh_Post-Veraison
Seed_Pre-Veraison
Seed_Veraison
Seed_Post-Veraison

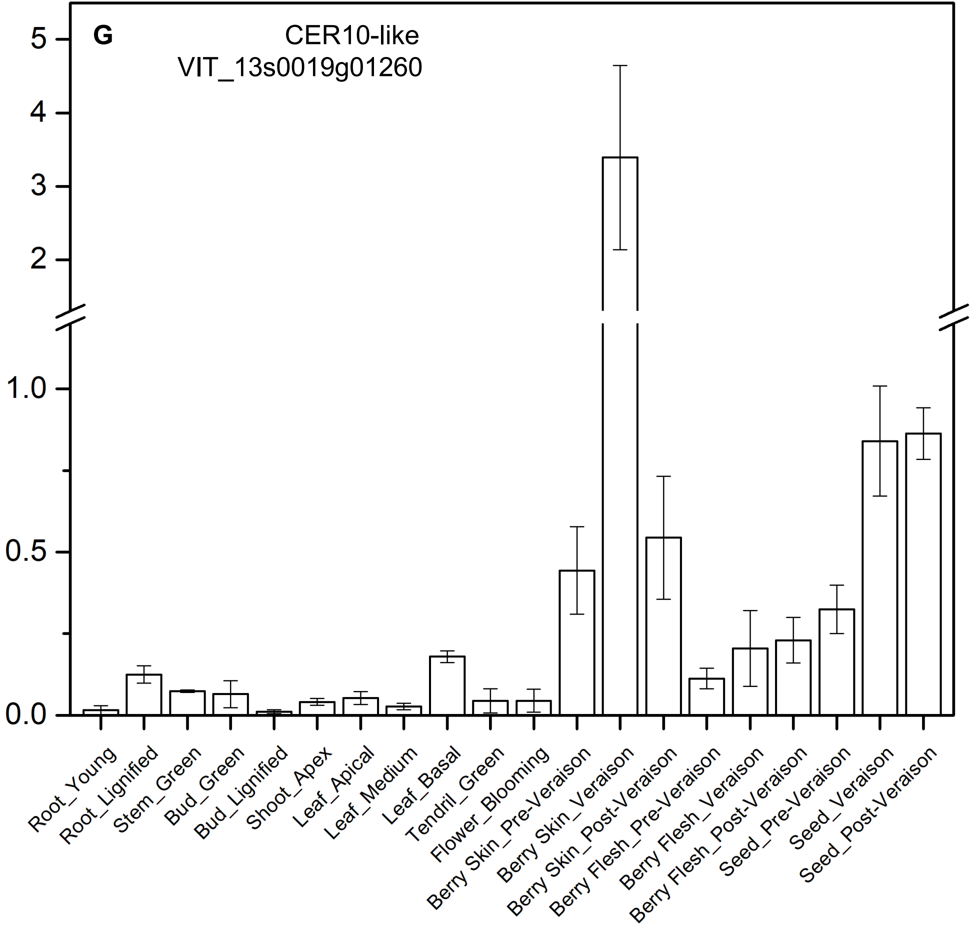
G
CER10-like
VIT_13s0019g01260
5
4
3
2
1.0
0.5
0.0
Root_Young
Root_Lignified
Stem_Green
Bud_Green
Bud_Lignified
Shoot_Apex
Leaf_Apical
Leaf_Medium
Leaf_Basal
Tendril_Green
Flower_Blooming
Berry Skin_Pre-Veraison
Berry Skin_Veraison
Berry Skin_Post-Veraison
Berry Flesh_Pre-Veraison
Berry Flesh_Veraison
Berry Flesh_Post-Veraison
Seed_Pre-Veraison
Seed_Veraison
Seed_Post-Veraison

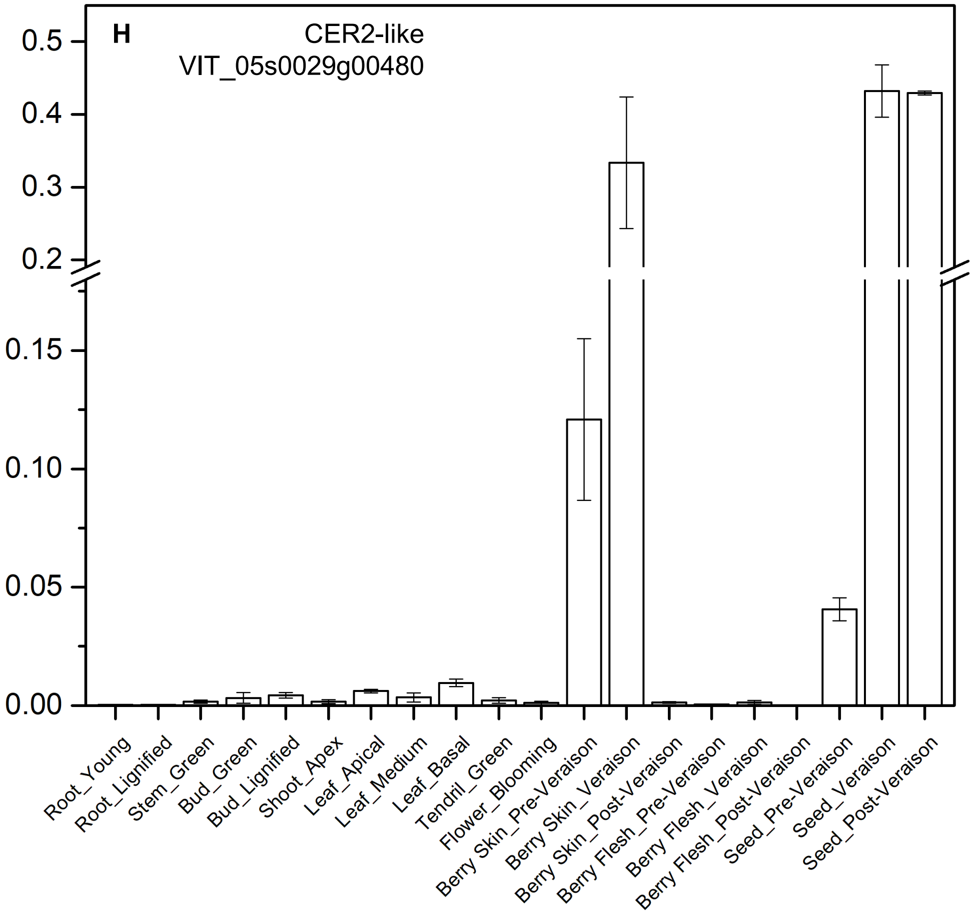
H
CER2-like
VIT_05s0029g00480
0.5
0.4
0.3
0.2
0.15
0.10
0.05
0.00
Root_Young
Root_Lignified
Stem_Green
Bud_Green
Bud_Lignified
Shoot_Apex
Leaf_Apical
Leaf_Medium
Leaf_Basal
Tendril_Green
Flower_Blooming
Berry Skin_Pre-Veraison
Berry Skin_Veraison
Berry Skin_Post-Veraison
Berry Flesh_Pre-Veraison
Berry Flesh_Veraison
Berry Flesh_Post-Veraison
Seed_Pre-Veraison
Seed_Veraison
Seed_Post-Veraison

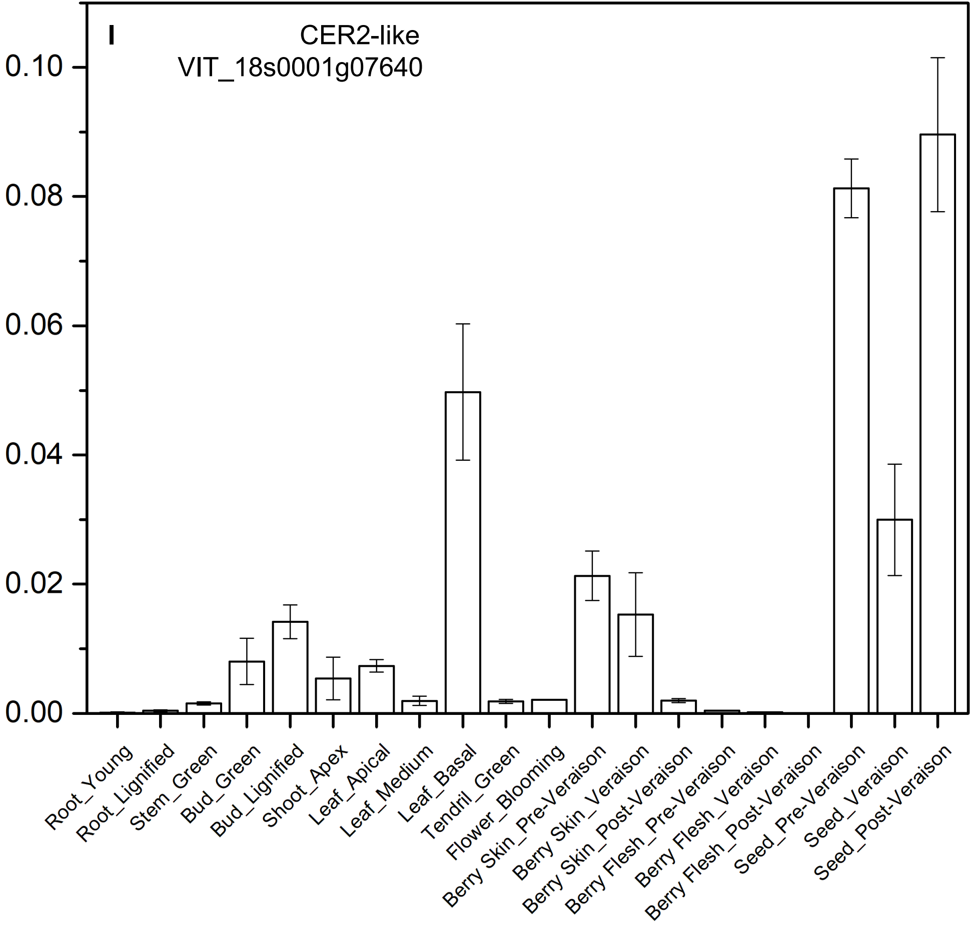
I
CER2-like
VIT_18s0001g07640
0.10
0.08
0.06
0.04
0.02
0.00
Root_Young
Root_Lignified
Stem_Green
Bud_Green
Bud_Lignified
Shoot_Apex
Leaf_Apical
Leaf_Medium
Leaf_Basal
Tendril_Green
Flower_Blooming
Berry Skin_Pre-Veraison
Berry Skin_Veraison
Berry Skin_Post-Veraison
Berry Flesh_Pre-Veraison
Berry Flesh_Veraison
Berry Flesh_Post-Veraison
Seed_Pre-Veraison
Seed_Veraison
Seed_Post-Veraison

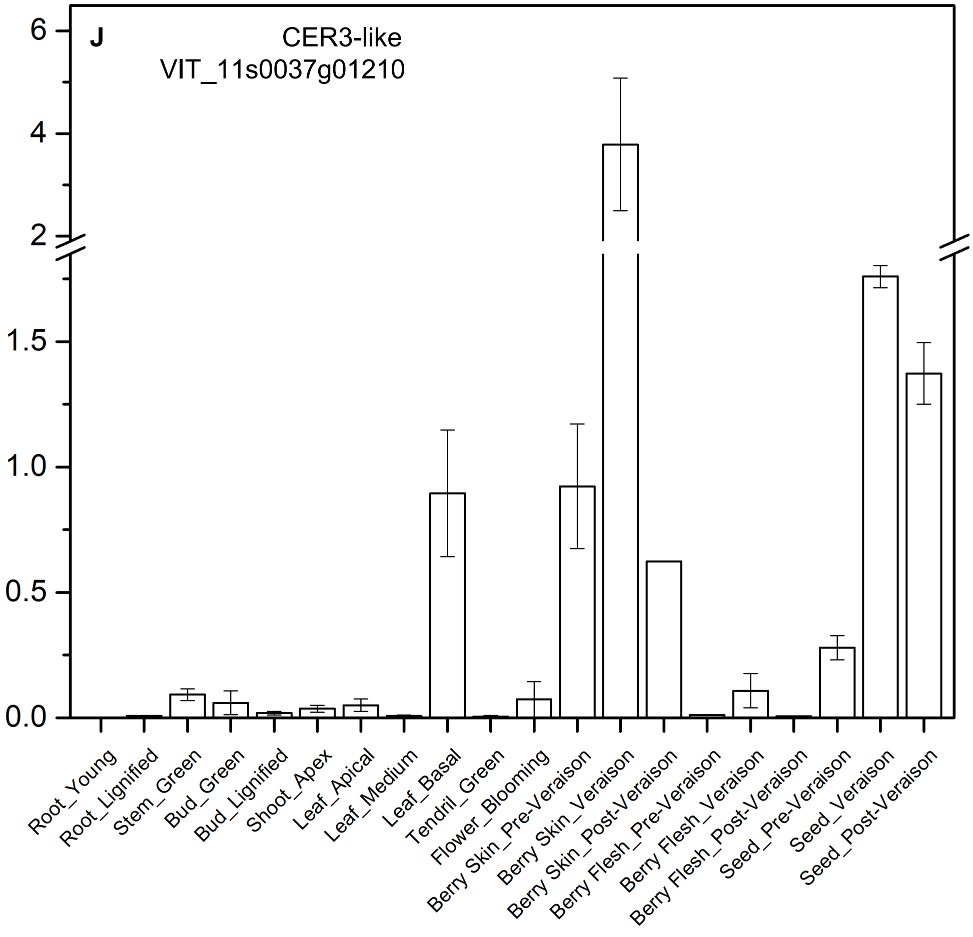
J
CER3-like
VIT_11s0037g01210
6
4
2
1.5
1.0
0.5
0.0
Root_Young
Root_Lignified
Stem_Green
Bud_Green
Bud_Lignified
Shoot_Apex
Leaf_Apical
Leaf_Medium
Leaf_Basal
Tendril_Green
Flower_Blooming
Berry Skin_Pre-Veraison
Berry Skin_Veraison
Berry Skin_Post-Veraison
Berry Flesh_Pre-Veraison
Berry Flesh_Veraison
Berry Flesh_Post-Veraison
Seed_Pre-Veraison
Seed_Veraison
Seed_Post-Veraison

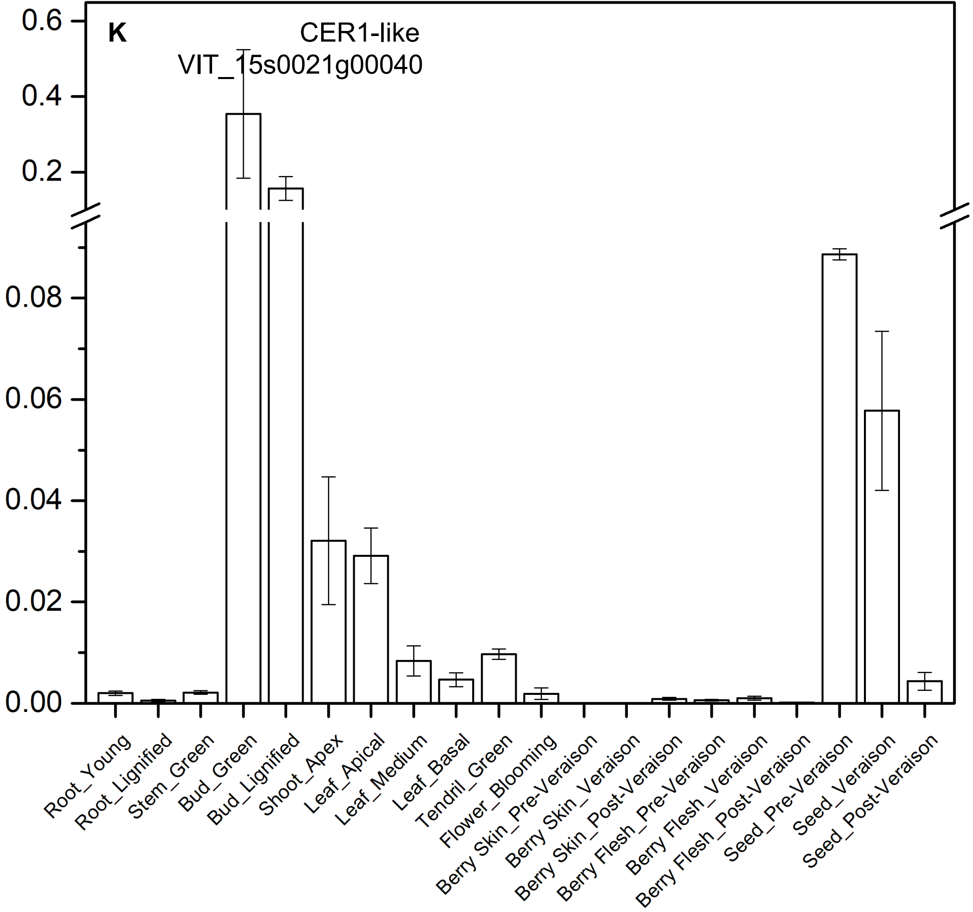
K
CER1-like
VIT_15s0021g00040
0.6
0.4
0.2
0.08
0.06
0.04
0.02
0.00
Root_Young
Root_Lignified
Stem_Green
Bud_Green
Bud_Lignified
Shoot_Apex
Leaf_Apical
Leaf_Medium
Leaf_Basal
Tendril_Green
Flower_Blooming
Berry Skin_Pre-Veraison
Berry Skin_Veraison
Berry Skin_Post-Veraison
Berry Flesh_Pre-Veraison
Berry Flesh_Veraison
Berry Flesh_Post-Veraison
Seed_Pre-Veraison
Seed_Veraison
Seed_Post-Veraison

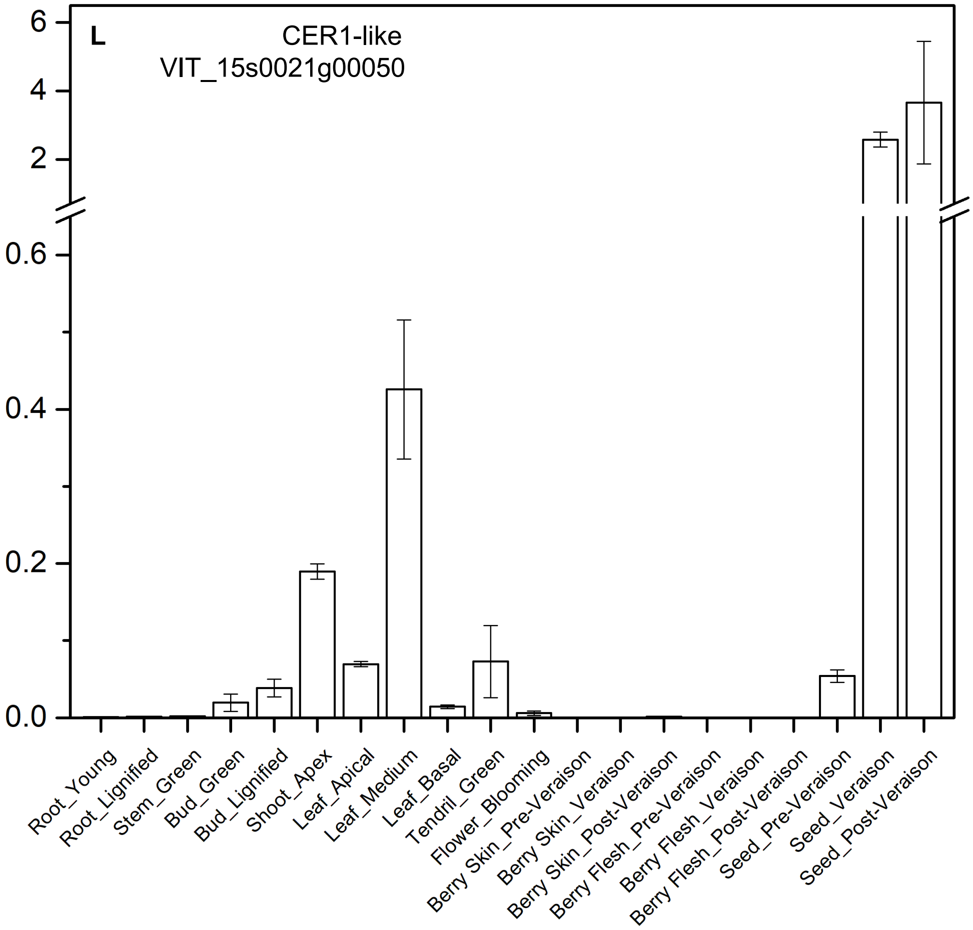
L
CER1-like
VIT_15s0021g00050
6
4
2
0.6
0.4
0.2
0.0
Root_Young
Root_Lignified
Stem_Green
Bud_Green
Bud_Lignified
Shoot_Apex
Leaf_Apical
Leaf_Medium
Leaf_Basal
Tendril_Green
Flower_Blooming
Berry Skin_Pre-Veraison
Berry Skin_Veraison
Berry Skin_Post-Veraison
Berry Flesh_Pre-Veraison
Berry Flesh_Veraison
Berry Flesh_Post-Veraison
Seed_Pre-Veraison
Seed_Veraison
Seed_Post-Veraison

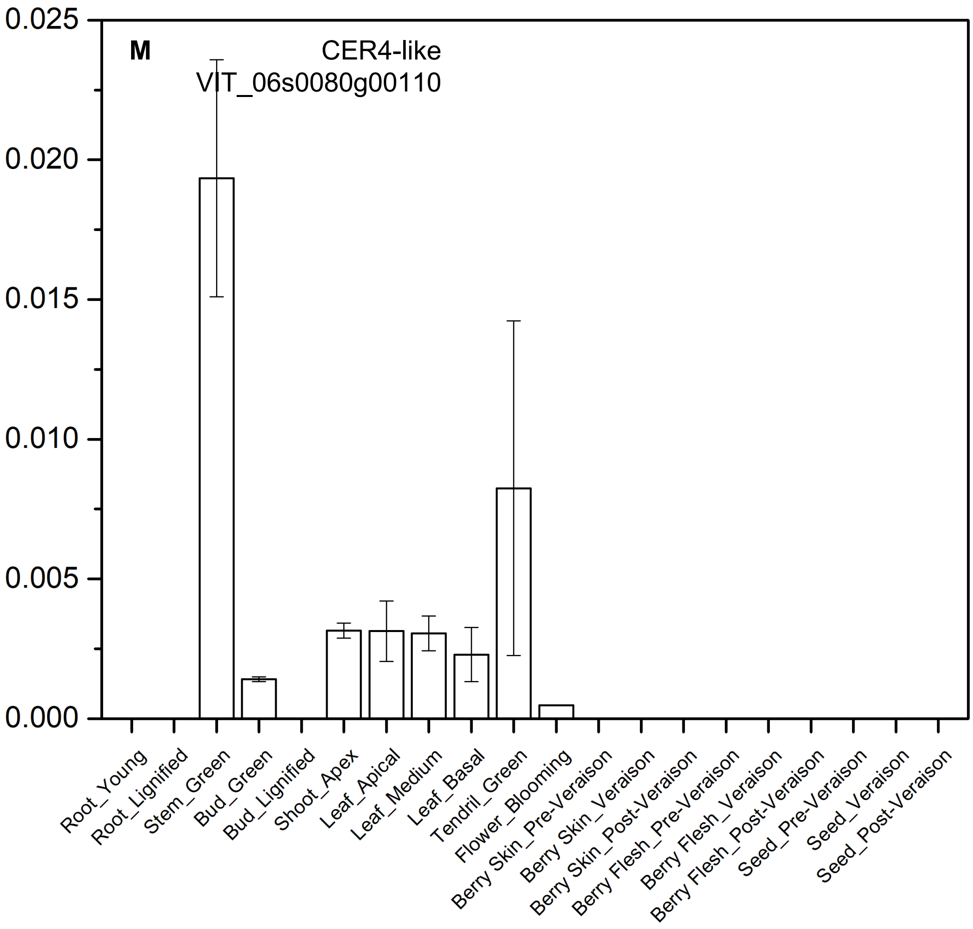

M
CER4-like
VIT_06s0080g00110
0.025
0.020
0.015
0.010
0.005
0.000
Root_Young
Root_Lignified
Stem_Green
Bud_Green
Bud_Lignified
Shoot_Apex
Leaf_Apical
Leaf_Medium
Leaf_Basal
Tendril_Green
Flower_Blooming
Berry Skin_Pre-Veraison
Berry Skin_Veraison
Berry Skin_Post-Veraison
Berry Flesh_Pre-Veraison
Berry Flesh_Veraison
Berry Flesh_Post-Veraison
Seed_Pre-Veraison
Seed_Veraison
Seed_Post-Veraison

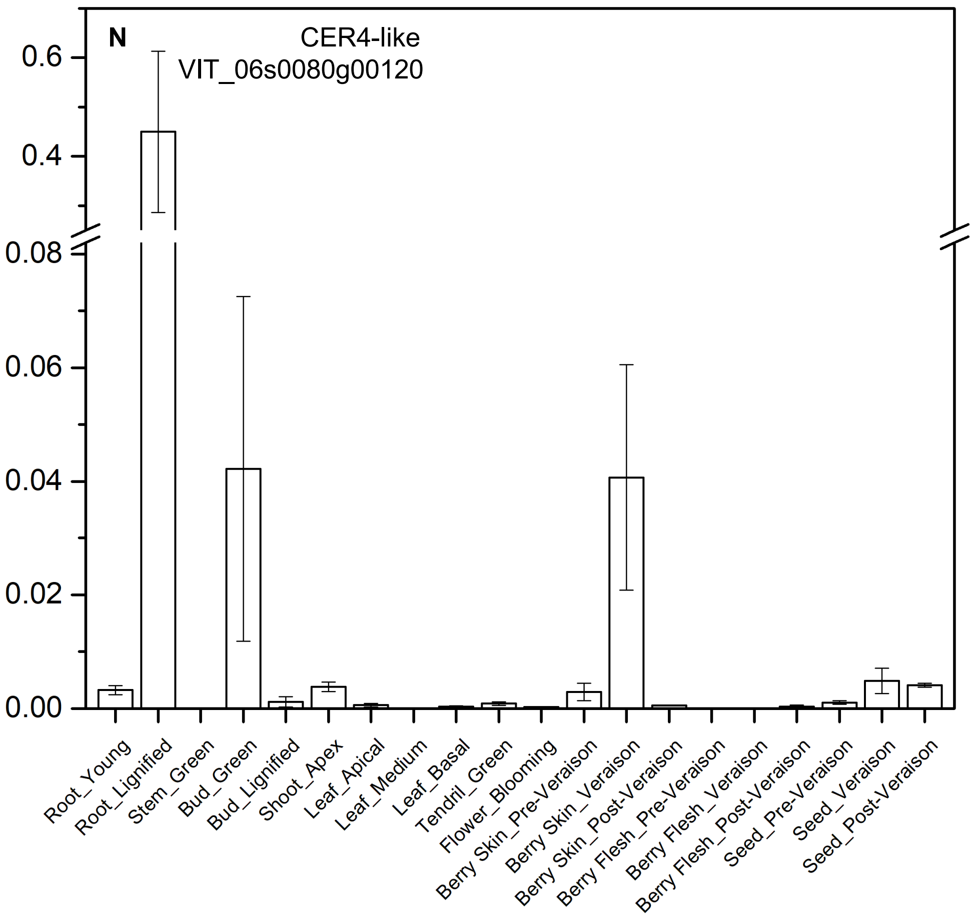

N
CER4-like
VIT_06s0080g00120
0.6
0.4
0.08
0.06
0.04
0.02
0.00
Root_Young
Root_Lignified
Stem_Green
Bud_Green
Bud_Lignified
Shoot_Apex
Leaf_Apical
Leaf_Medium
Leaf_Basal
Tendril_Green
Flower_Blooming
Berry Skin_Pre-Veraison
Berry Skin_Veraison
Berry Skin_Post-Veraison
Berry Flesh_Pre-Veraison
Berry Flesh_Veraison
Berry Flesh_Post-Veraison
Seed_Pre-Veraison
Seed_Veraison
Seed_Post-Veraison

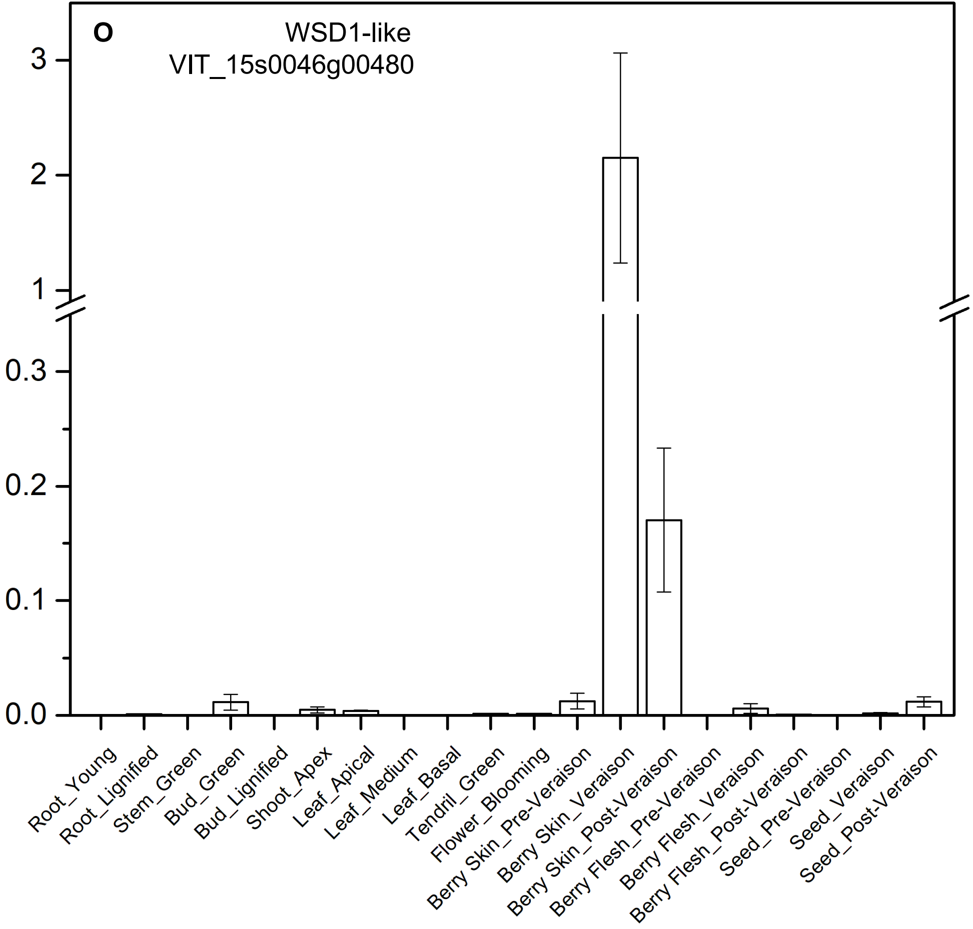
O
WSD1-like
VIT_15s0046g00480
3
2
1
0.3
0.2
0.1
0.0
Root_Young
Root_Lignified
Stem_Green
Bud_Green
Bud_Lignified
Shoot_Apex
Leaf_Apical
Leaf_Medium
Leaf_Basal
Tendril_Green
Flower_Blooming
Berry Skin_Pre-Veraison
Berry Skin_Veraison
Berry Skin_Post-Veraison
Berry Flesh_Pre-Veraison
Berry Flesh_Veraison
Berry Flesh_Post-Veraison
Seed_Pre-Veraison
Seed_Veraison
Seed_Post-Veraison

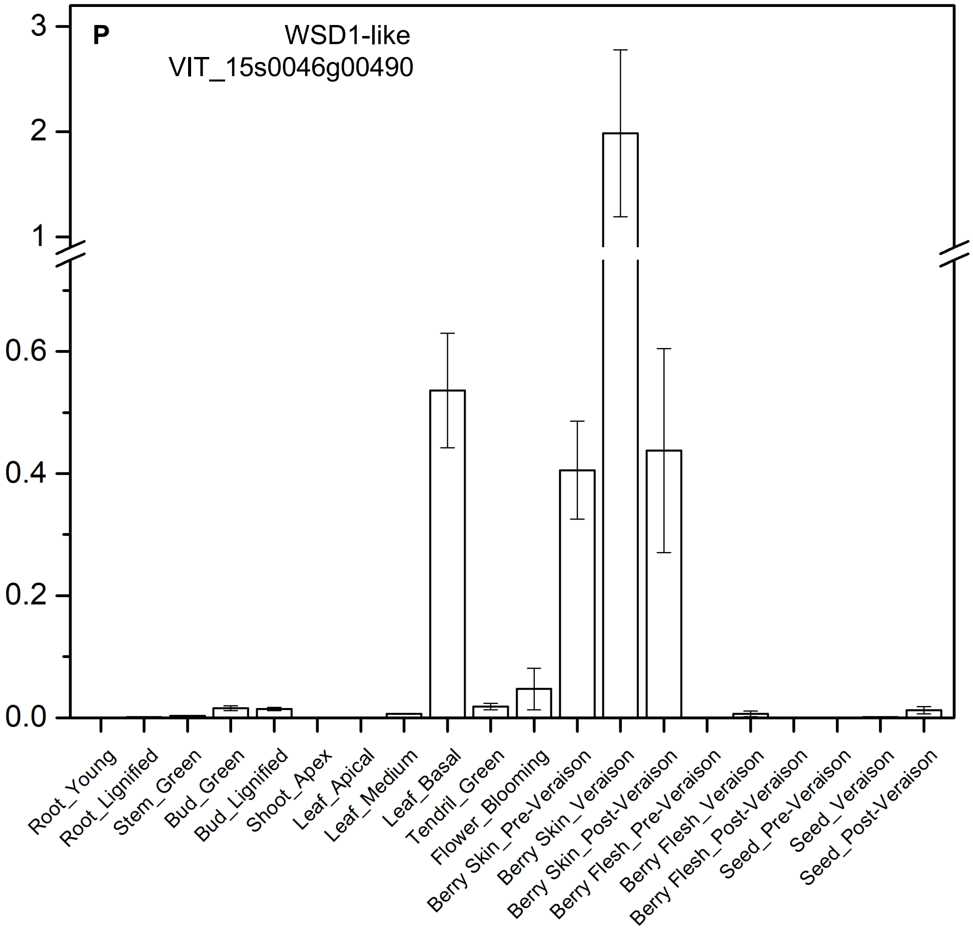
P
WSD1-like
VIT_15s0046g00490
3
2
1
0.6
0.4
0.2
0.0
Root_Young
Root_Lignified
Stem_Green
Bud_Green
Bud_Lignified
Shoot_Apex
Leaf_Apical
Leaf_Medium
Leaf_Basal
Tendril_Green
Flower_Blooming
Berry Skin_Pre-Veraison
Berry Skin_Veraison
Berry Skin_Post-Veraison
Berry Flesh_Pre-Veraison
Berry Flesh_Veraison
Berry Flesh_Post-Veraison
Seed_Pre-Veraison
Seed_Veraison
Seed_Post-Veraison

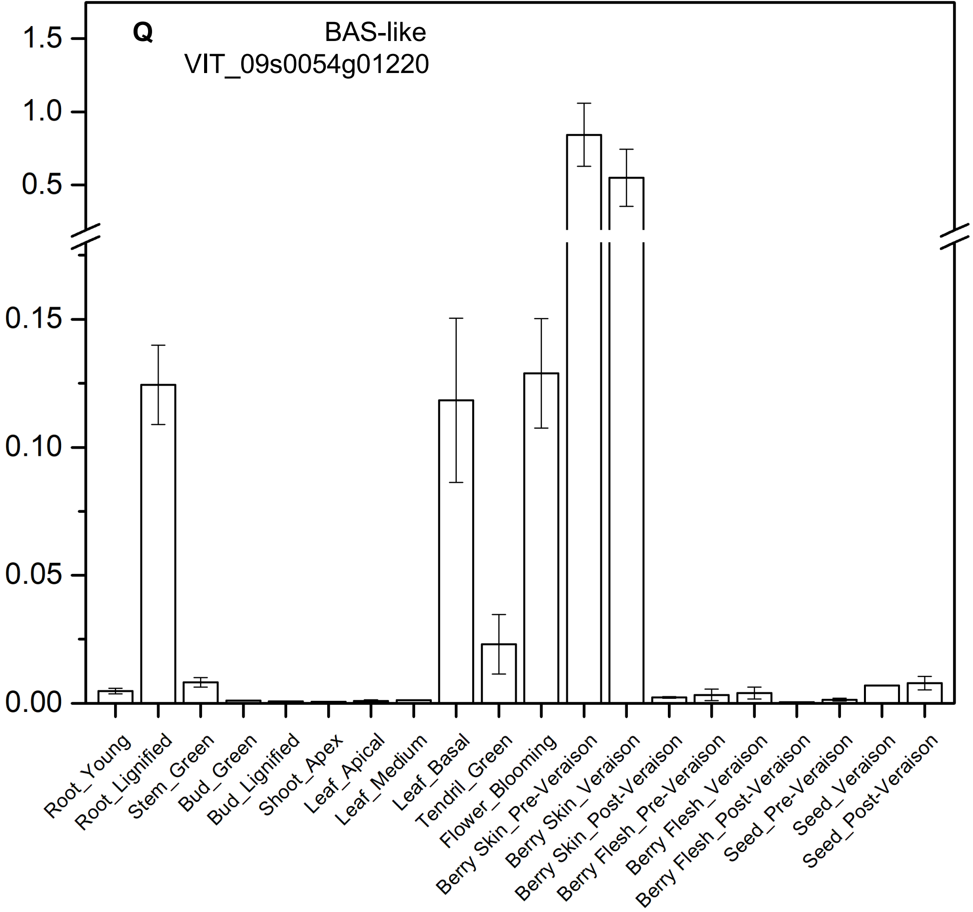
Q
BAS-like
VIT_09s0054g01220
1.5
1.0
0.5
0.15
0.10
0.05
0.00
Root_Young
Root_Lignified
Stem_Green
Bud_Green
Bud_Lignified
Shoot_Apex
Leaf_Apical
Leaf_Medium
Leaf_Basal
Tendril_Green
Flower_Blooming
Berry Skin_Pre-Veraison
Berry Skin_Veraison
Berry Skin_Post-Veraison
Berry Flesh_Pre-Veraison
Berry Flesh_Veraison
Berry Flesh_Post-Veraison
Seed_Pre-Veraison
Seed_Veraison
Seed_Post-Veraison

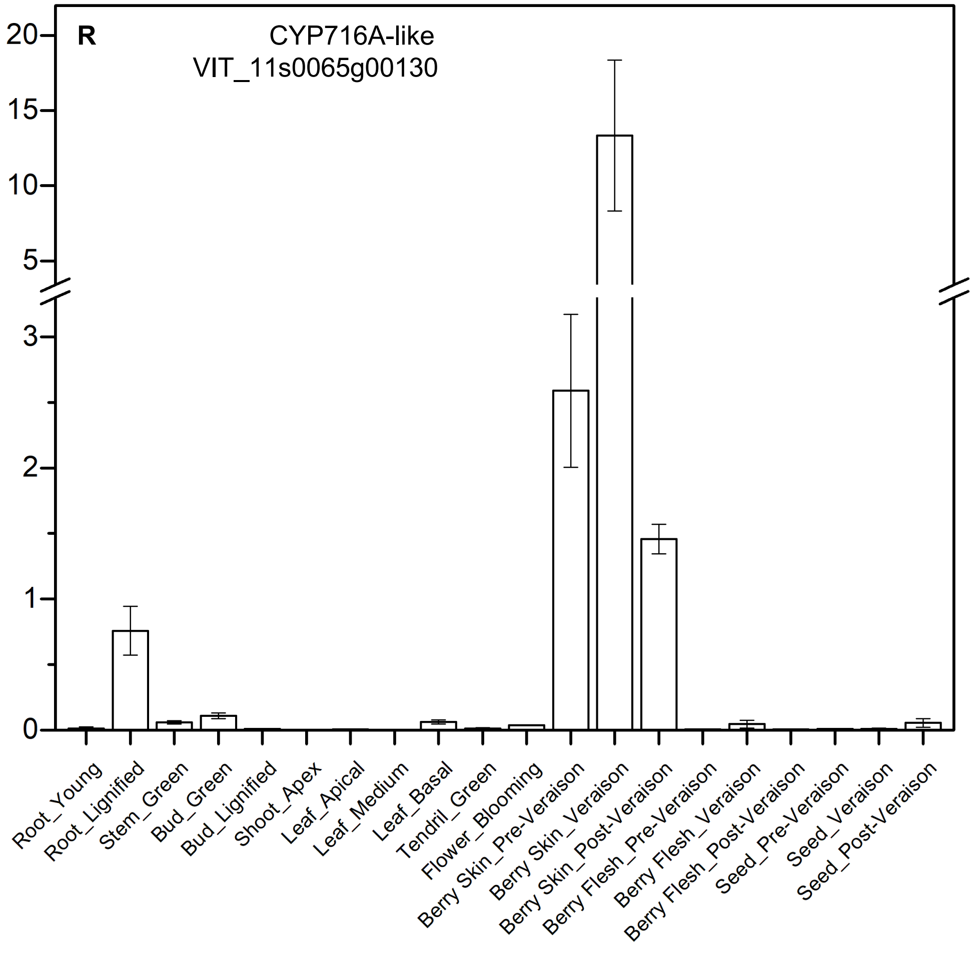
R
CYP716A-like
VIT_11s0065g00130
20
15
10
5
3
2
1
0
Root_Young
Root_Lignified
Stem_Green
Bud_Green
Bud_Lignified
Shoot_Apex
Leaf_Apical
Leaf_Medium
Leaf_Basal
Tendril_Green
Flower_Blooming
Berry Skin_Pre-Veraison
Berry Skin_Veraison
Berry Skin_Post-Veraison
Berry Flesh_Pre-Veraison
Berry Flesh_Veraison
Berry Flesh_Post-Veraison
Seed_Pre-Veraison
Seed_Veraison
Seed_Post-Veraison

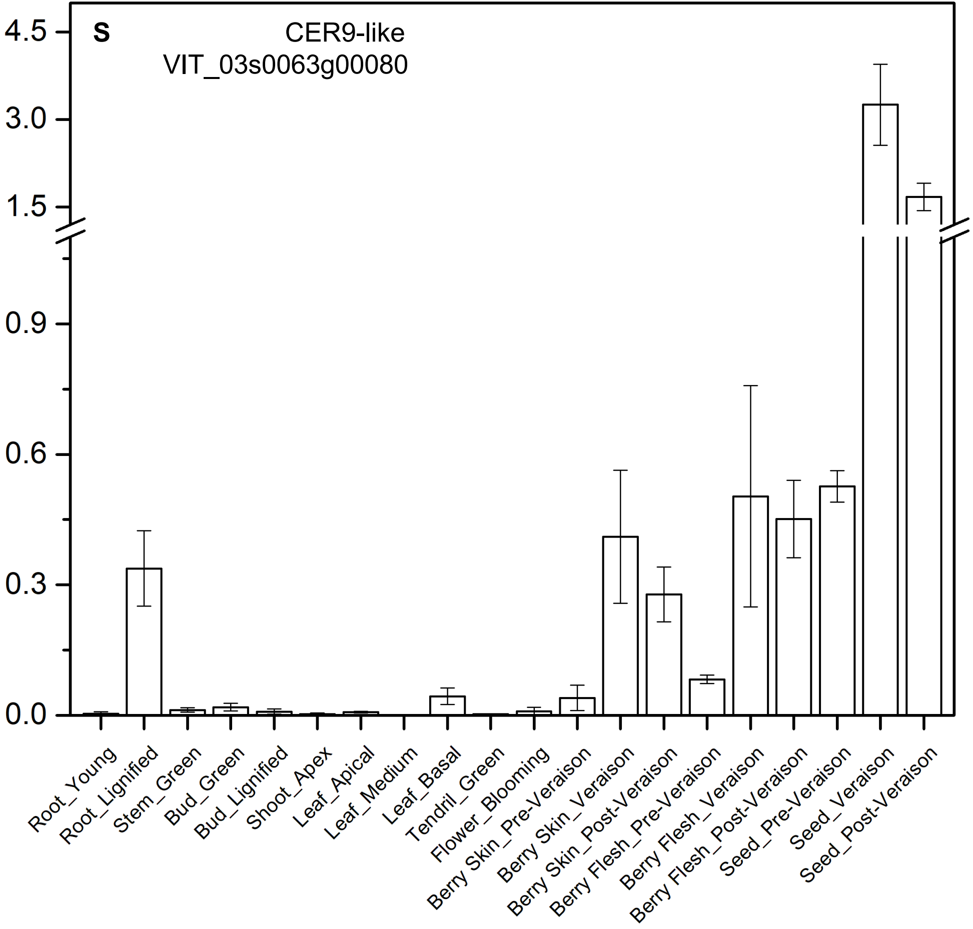
S
CER9-like
VIT_03s0063g00080
4.5
3.0
1.5
0.9
0.6
0.3
0.0
Root_Young
Root_Lignified
Stem_Green
Bud_Green
Bud_Lignified
Shoot_Apex
Leaf_Apical
Leaf_Medium
Leaf_Basal
Tendril_Green
Flower_Blooming
Berry Skin_Pre-Veraison
Berry Skin_Veraison
Berry Skin_Post-Veraison
Berry Flesh_Pre-Veraison
Berry Flesh_Veraison
Berry Flesh_Post-Veraison
Seed_Pre-Veraison
Seed_Veraison
Seed_Post-Veraison

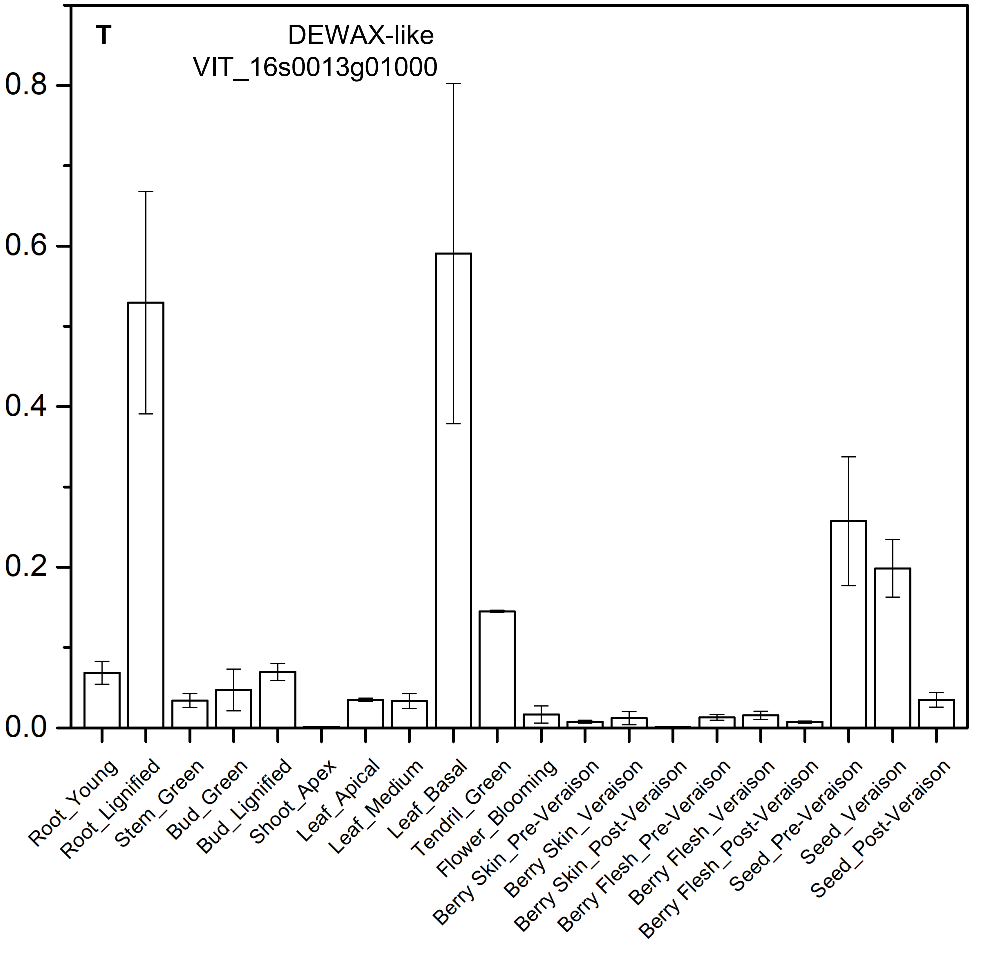
T
DEWAX-like
VIT_16s0013g01000
0.8
0.6
0.4
0.2
0.0
Root_Young
Root_Lignified
Stem_Green
Bud_Green
Bud_Lignified
Shoot_Apex
Leaf_Apical
Leaf_Medium
Leaf_Basal
Tendril_Green
Flower_Blooming
Berry Skin_Pre-Veraison
Berry Skin_Veraison
Berry Skin_Post-Veraison
Berry Flesh_Pre-Veraison
Berry Flesh_Veraison
Berry Flesh_Post-Veraison
Seed_Pre-Veraison
Seed_Veraison
Seed_Post-Veraison

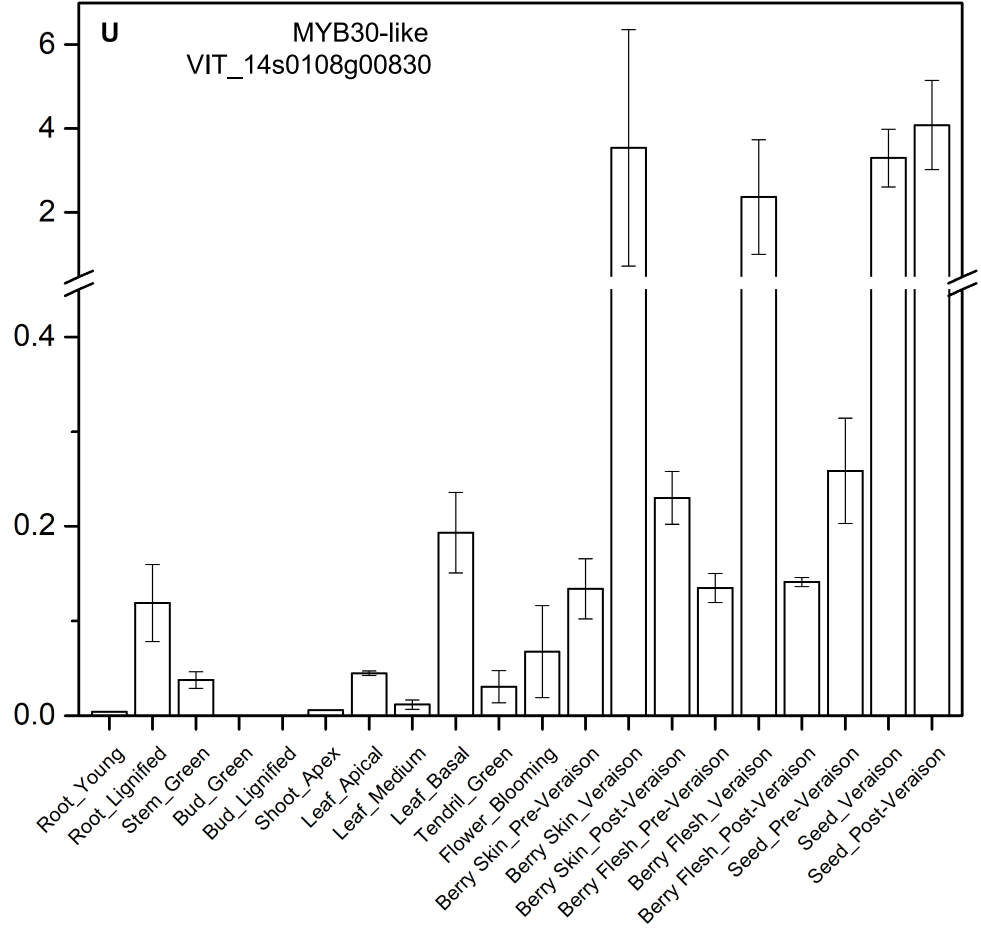
U
MYB30-like
VIT_14s0108g00830
6
4
2
0.4
0.2
0.0
Root_Young
Root_Lignified
Stem_Green
Bud_Green
Bud_Lignified
Shoot_Apex
Leaf_Apical
Leaf_Medium
Leaf_Basal
Tendril_Green
Flower_Blooming
Berry Skin_Pre-Veraison
Berry Skin_Veraison
Berry Skin_Post-Veraison
Berry Flesh_Pre-Veraison
Berry Flesh_Veraison
Berry Flesh_Post-Veraison
Seed_Pre-Veraison
Seed_Veraison
Seed_Post-Veraison

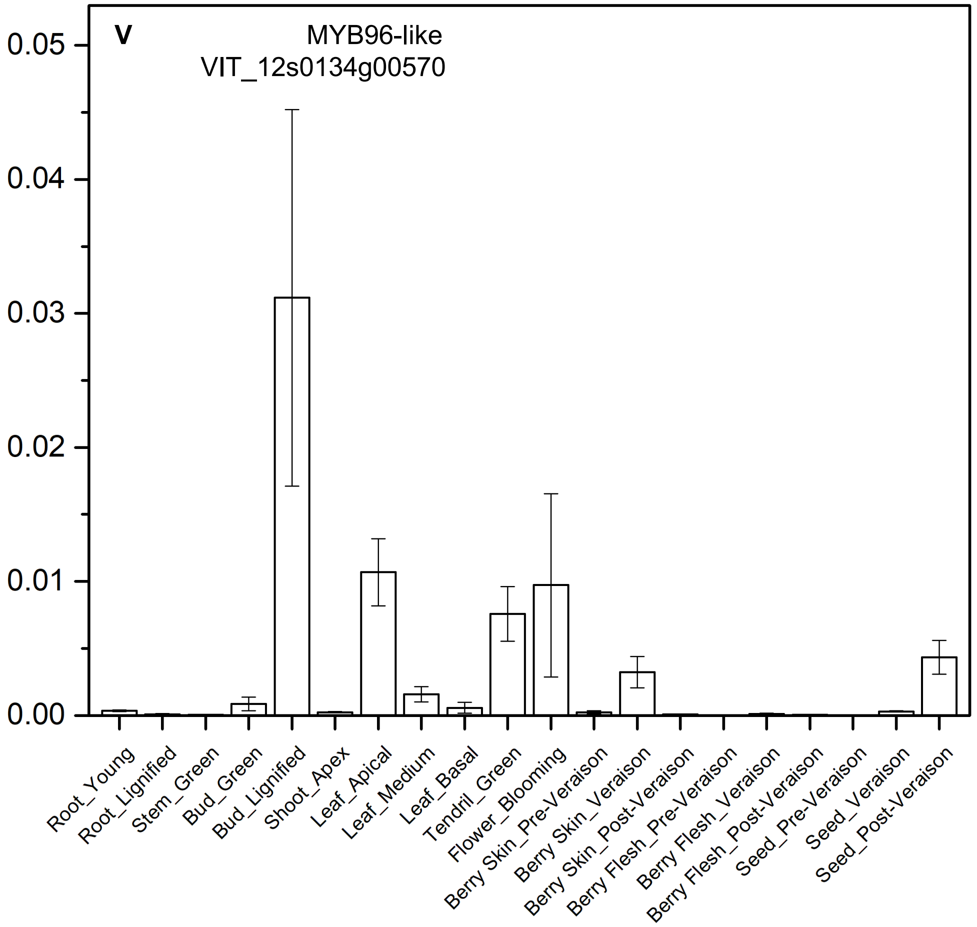
V
MYB96-like
VIT_12s0134g00570
0.05
0.04
0.03
0.02
0.01
0.00
Root_Young
Root_Lignified
Stem_Green
Bud_Green
Bud_Lignified
Shoot_Apex
Leaf_Apical
Leaf_Medium
Leaf_Basal
Tendril_Green
Flower_Blooming
Berry Skin_Pre-Veraison
Berry Skin_Veraison
Berry Skin_Post-Veraison
Berry Flesh_Pre-Veraison
Berry Flesh_Veraison
Berry Flesh_Post-Veraison
Seed_Pre-Veraison
Seed_Veraison
Seed_Post-Veraison

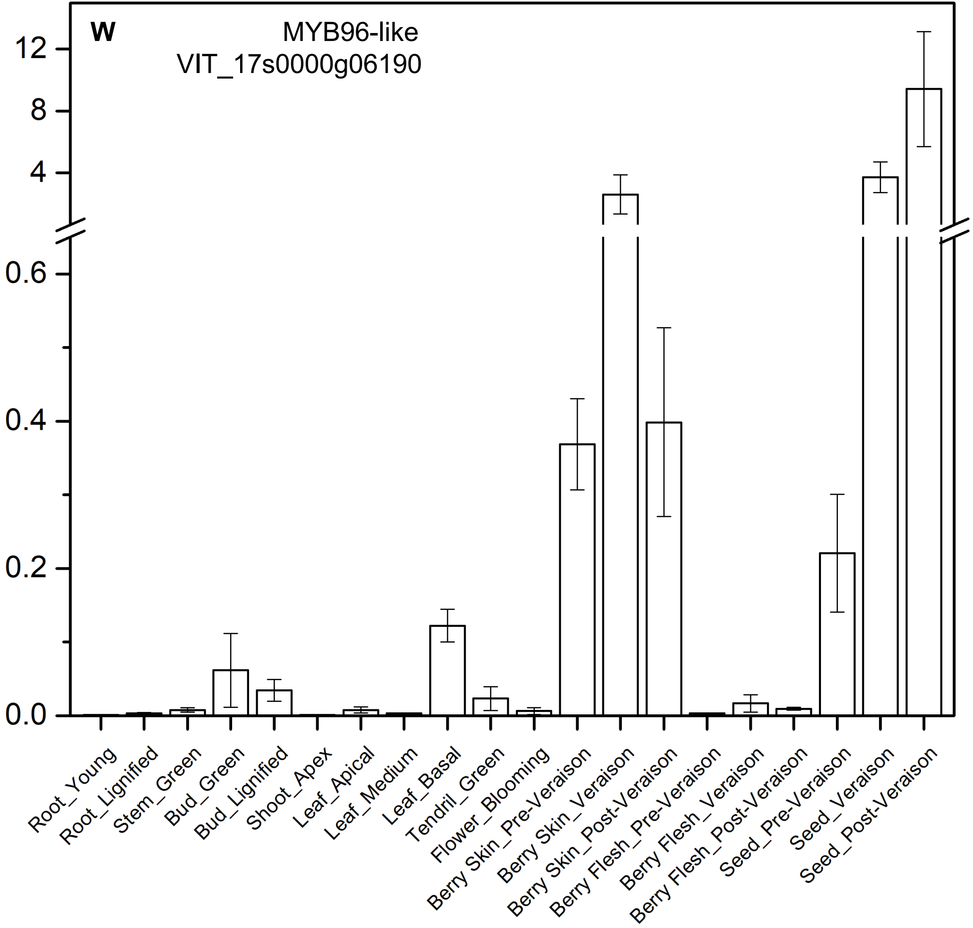

W
MYB96-like
VIT_17s0000g06190
12
8
4
0.6
0.4
0.2
0.0
Root_Young
Root_Lignified
Stem_Green
Bud_Green
Bud_Lignified
Shoot_Apex
Leaf_Apical
Leaf_Medium
Leaf_Basal
Tendril_Green
Flower_Blooming
Berry Skin_Pre-Veraison
Berry Skin_Veraison
Berry Skin_Post-Veraison
Berry Flesh_Pre-Veraison
Berry Flesh_Veraison
Berry Flesh_Post-Veraison
Seed_Pre-Veraison
Seed_Veraison
Seed_Post-Veraison

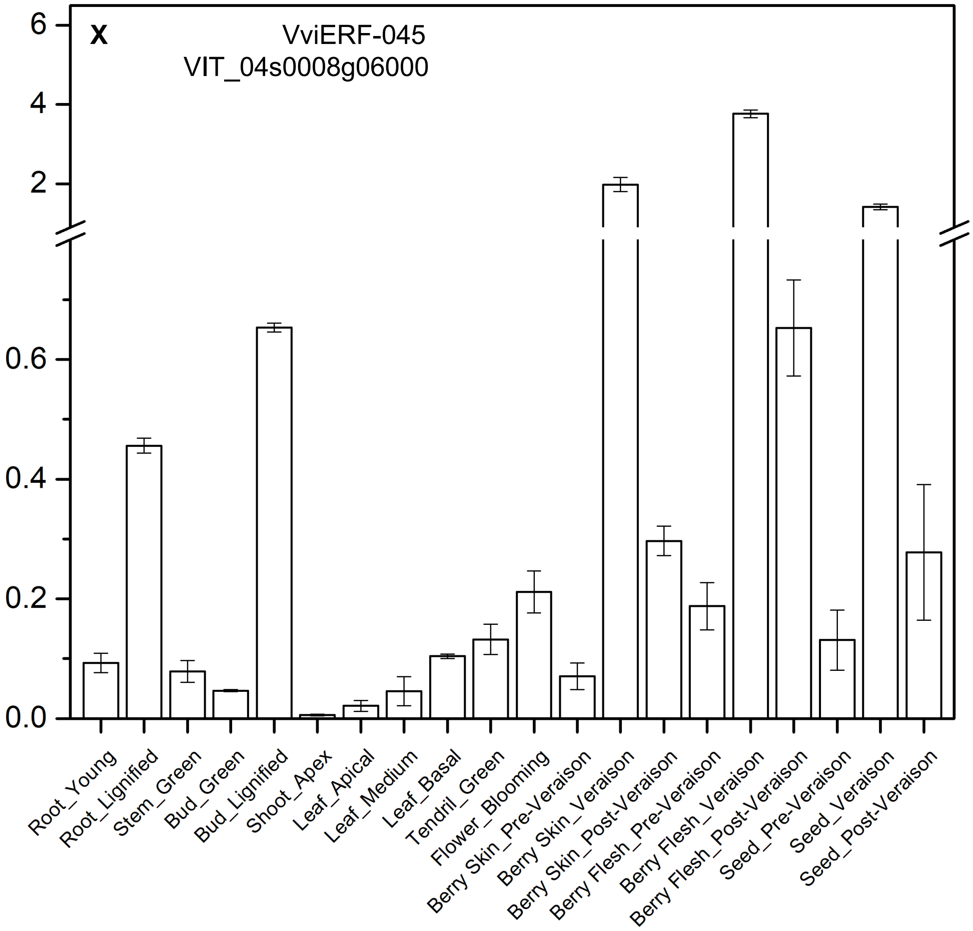

X
VviERF-045
VIT_04s0008g06000
6
4
2
0.6
0.4
0.2
0.0
Root_Young
Root_Lignified
Stem_Green
Bud_Green
Bud_Lignified
Shoot_Apex
Leaf_Apical
Leaf_Medium
Leaf_Basal
Tendril_Green
Flower_Blooming
Berry Skin_Pre-Veraison
Berry Skin_Veraison
Berry Skin_Post-Veraison
Berry Flesh_Pre-Veraison
Berry Flesh_Veraison
Berry Flesh_Post-Veraison
Seed_Pre-Veraison
Seed_Veraison
Seed_Post-Veraison

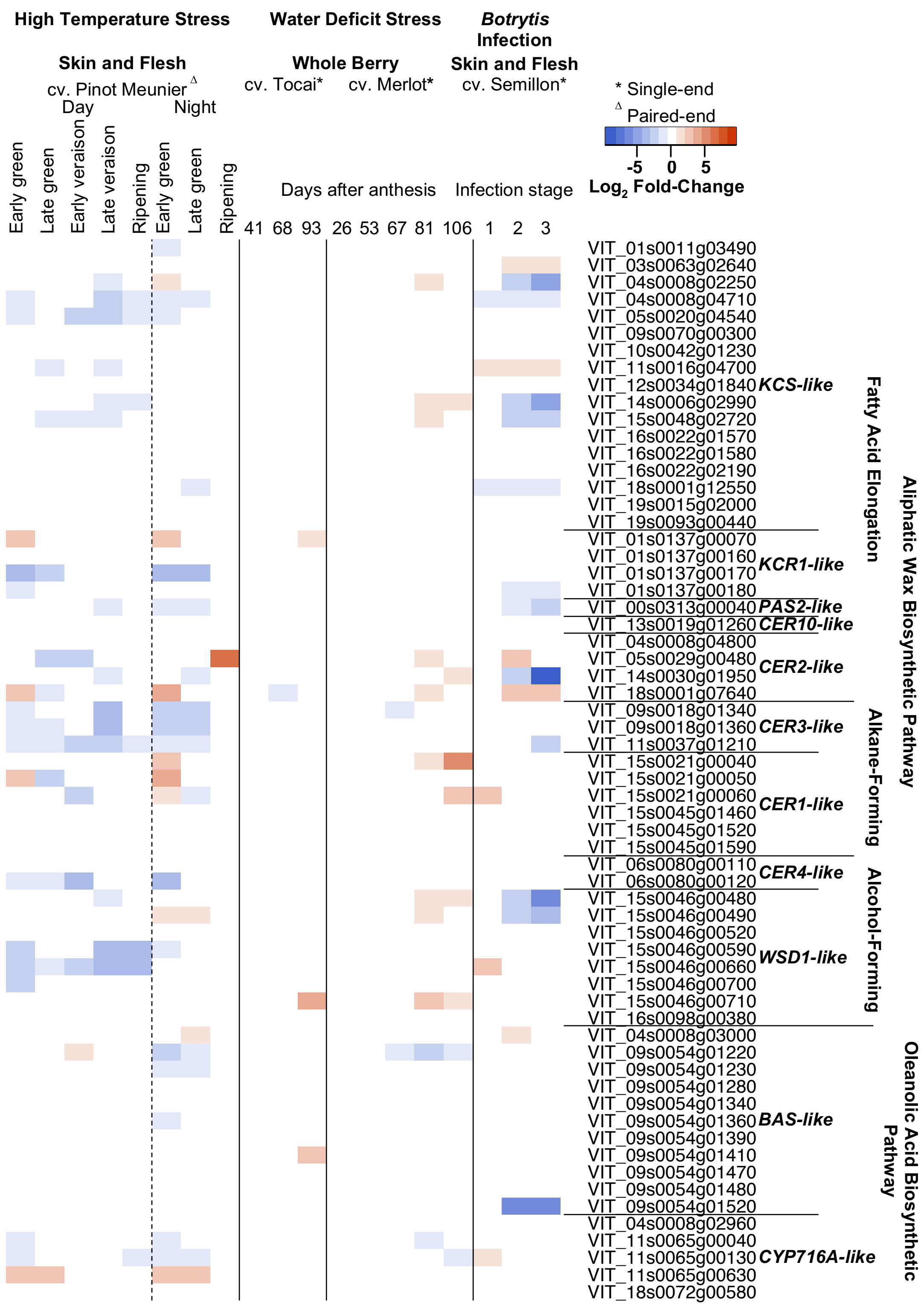

Figure S4. Heatmap of differential expression in terms of log2fold-change (FDR<0.05) of grapevine (*Vitis vinifera* L) putative homologs involved in cuticular aliphatic wax and oleanolic acid biosynthesis in grape berry tissues during development and under abiotic and biotic stresses. RNA-seq datasets were retrieved from the DNA data bank of Japan and reprocessed.

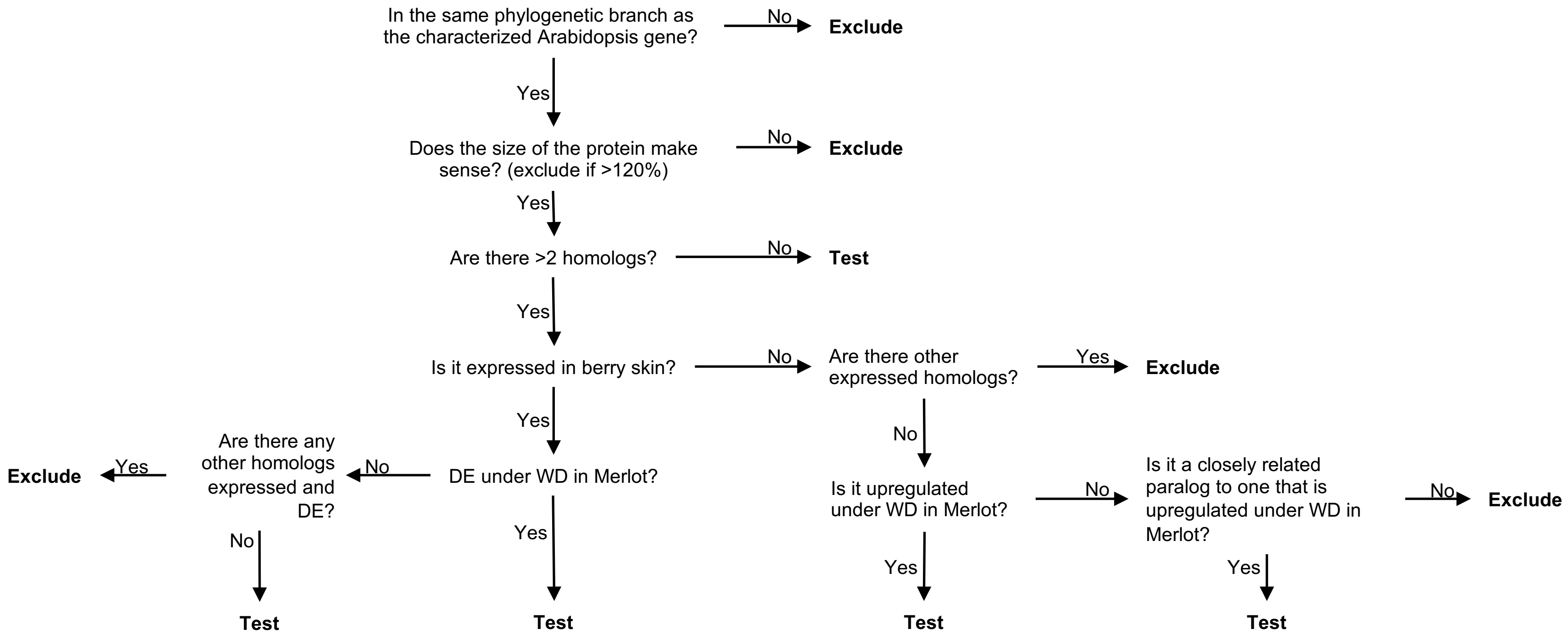

Figure S5. Decision tree used to determine if grapevine (*Vitis vinifera* L.) candidate biosynthetic gene involved in cuticular aliphatic wax or oleanolic acid biosynthesis is to be tested for expression in berry skin during a long term water deficit stress biological experiment on Merlot grape development.

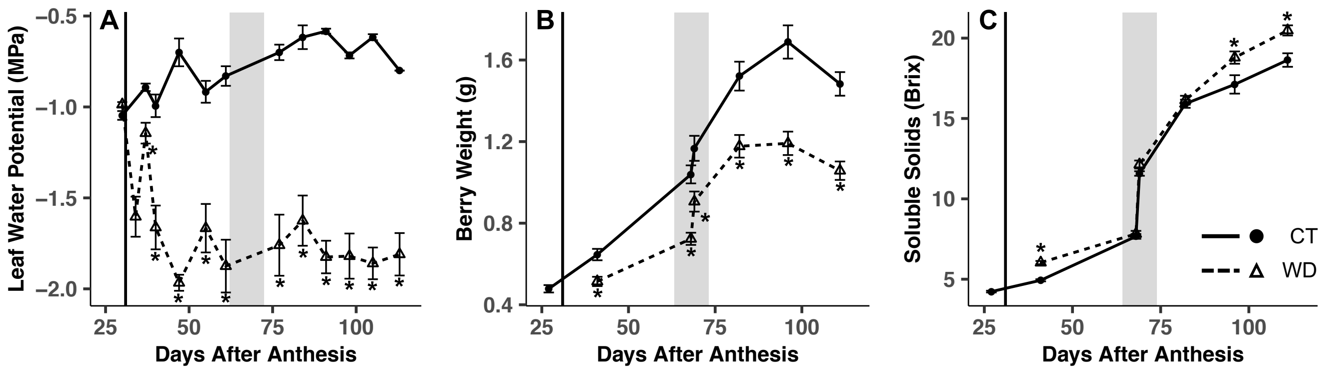

Figure S6. Leaf water potential **(A)**, berry growth **(B)**, and berry soluble solids **(C)** in grapevines (*Vitis vinifera* L) exposed to two irrigation treatments: well-irrigated (Control = CT) and deficit irrigated (Water Deficit = WD). Error bars represent ± S.E. and significant differences between treatments were determined by Two-Sample t-Test (*,P<0.05). The black vertical line represents the start of the deficit irrigation treatment, shaded background indicate when veraison occurred. The points at 68 and 69 days after anthesis (DAA) in **B** and **C** represent green berries at 68 DAA and red berries at 68 DAA, respectively.

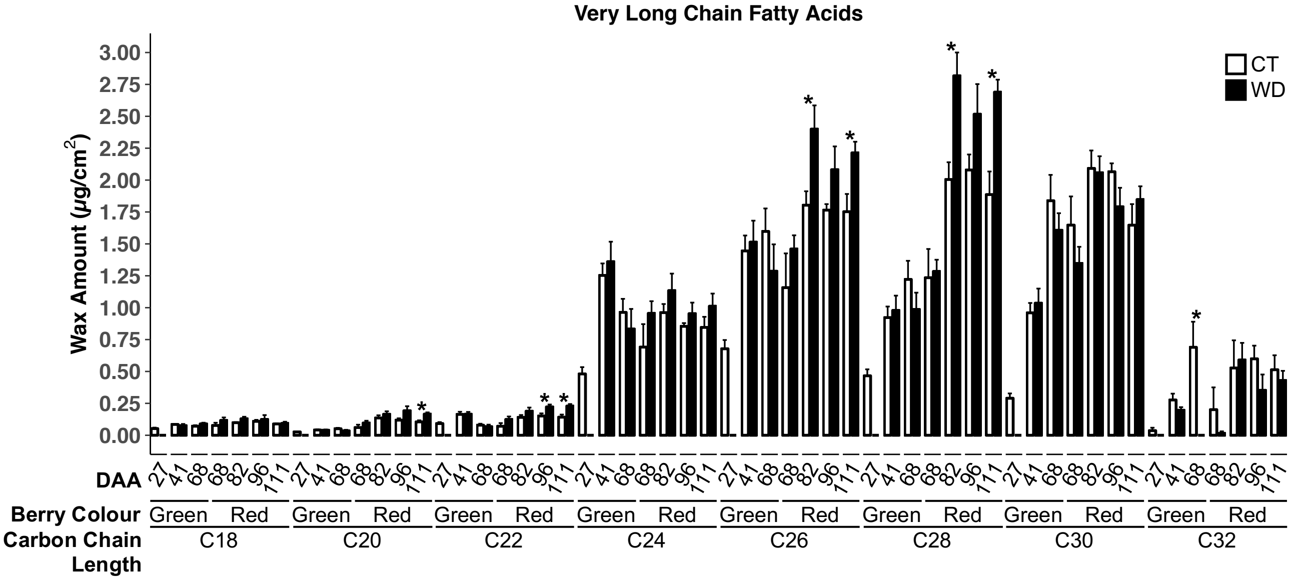

Figure S7. Cuticular wax composition in berries at 27, 41, 68, 82, 96, and 111 days after anthesis (DAA) of grapevines (*Vitis vinifera* L) exposed to two irrigation treatments: well-irrigated (Control = CT) and deficit irrigated (Water Deficit = WD). At 68 DAA, green berries were separated from red berries. Error bars represent ± S.E. and significant differences between treatments were determined by Two-Sample t-Test (*,P<0.05). Compound classes are produced by different parts of the cuticular wax biosynthetic pathway, with very long chain fatty acids made by the fatty acid elongase complex.

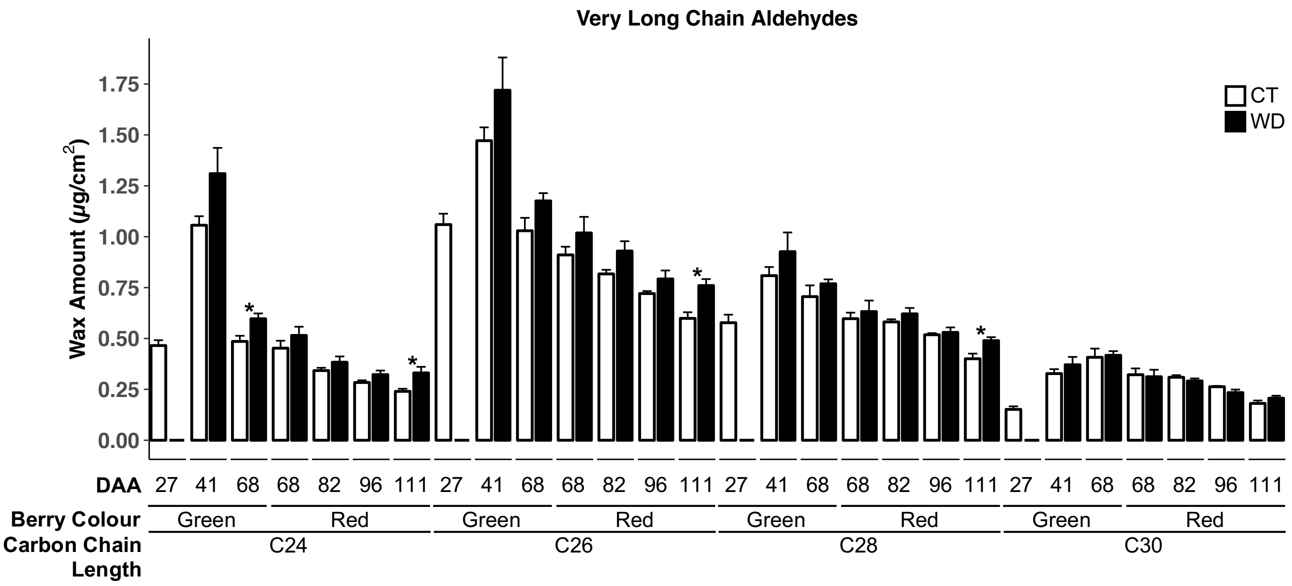

Figure S8. Cuticular wax composition in berries at 27, 41, 68, 82, 96, and 111 days after anthesis (DAA) of grapevines (*Vitis vinifera* L) exposed to two irrigation treatments: well-irrigated (Control = CT) and deficit irrigated (Water Deficit = WD). At 68 DAA, green berries were separated from red berries. Error bars represent ± S.E. and significant differences between treatments were determined by Two-Sample t-Test (*,P<0.05). Compound classes are produced by different parts of the cuticular wax biosynthetic pathway, with aldehydes made by the alkane forming branch.

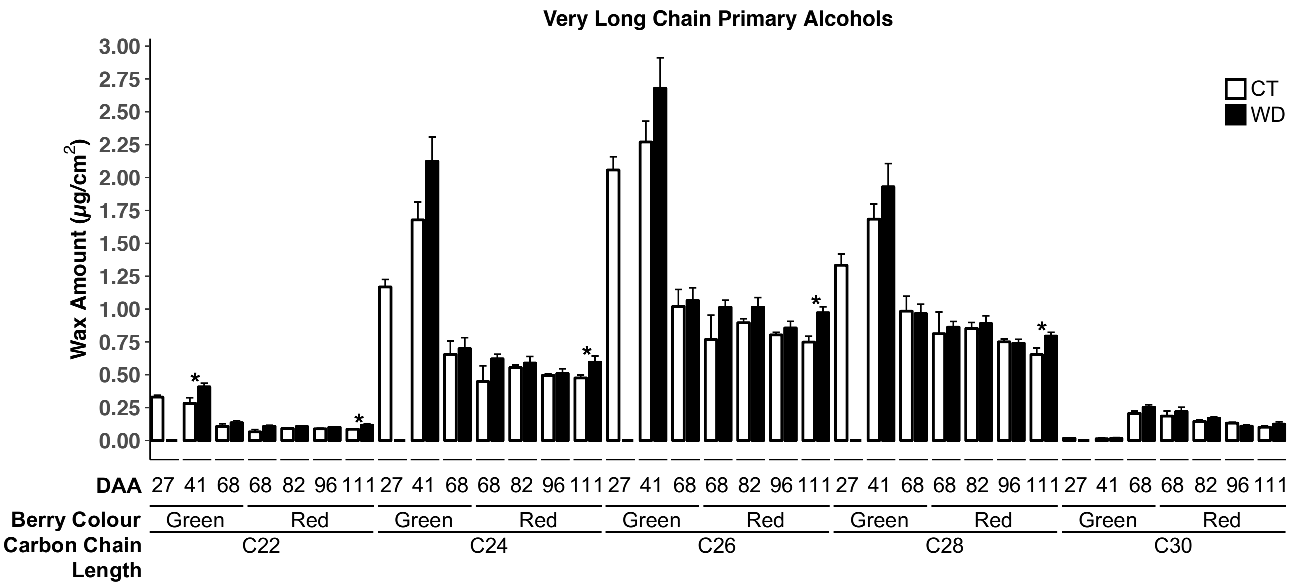

Figure S9. Cuticular wax composition in berries at 27, 41, 68, 82, 96, and 111 days after anthesis (DAA) of grapevines (*Vitis vinifera* L) exposed to two irrigation treatments: well-irrigated (Control = CT) and deficit irrigated (Water Deficit = WD). At 68 DAA, green berries were separated from red berries. Error bars represent ± S.E. and significant differences between treatments were determined by Two-Sample t-Test (*,P<0.05). Compound classes are produced by different parts of the cuticular wax biosynthetic pathway, with primary alcohols made by the alcohol forming branch.

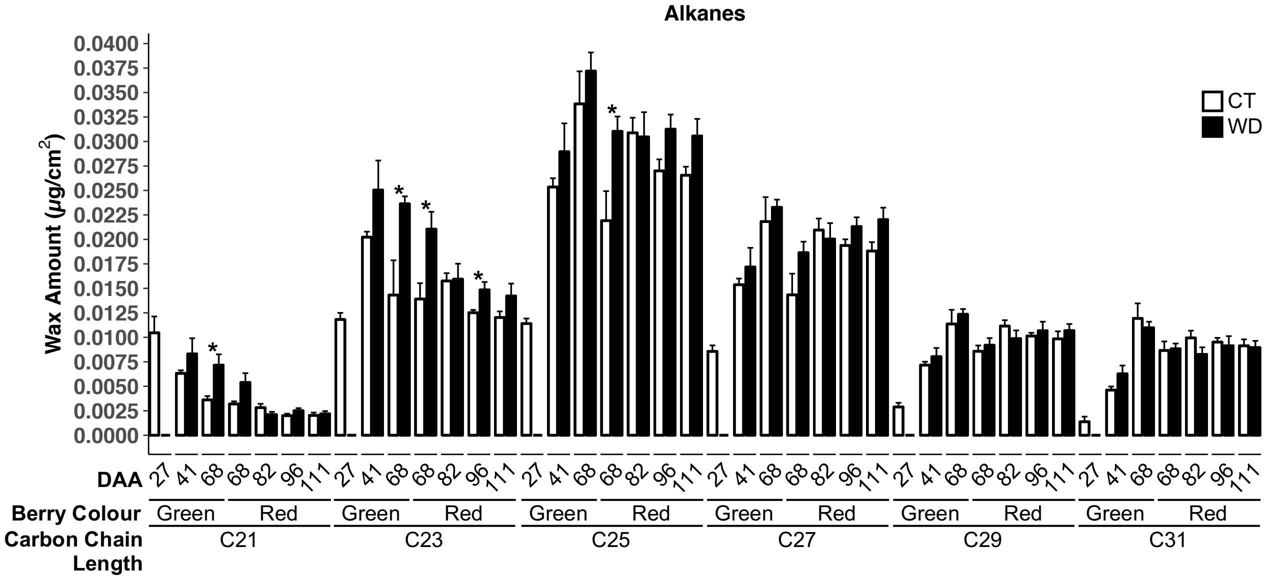

Figure S10. Cuticular wax composition in berries at 27, 41, 68, 82, 96, and 111 days after anthesis (DAA) of grapevines (*Vitis vinifera* L) exposed to two irrigation treatments: well-irrigated (Control = CT) and deficit irrigated (Water Deficit = WD). At 68 DAA, green berries were separated from red berries. Error bars represent ± S.E. and significant differences between treatments were determined by Two-Sample t-Test (*,$P<0.05$). Compound classes are produced by different parts of the cuticular wax biosynthetic pathway, with alkanes made by the alkane forming branch.

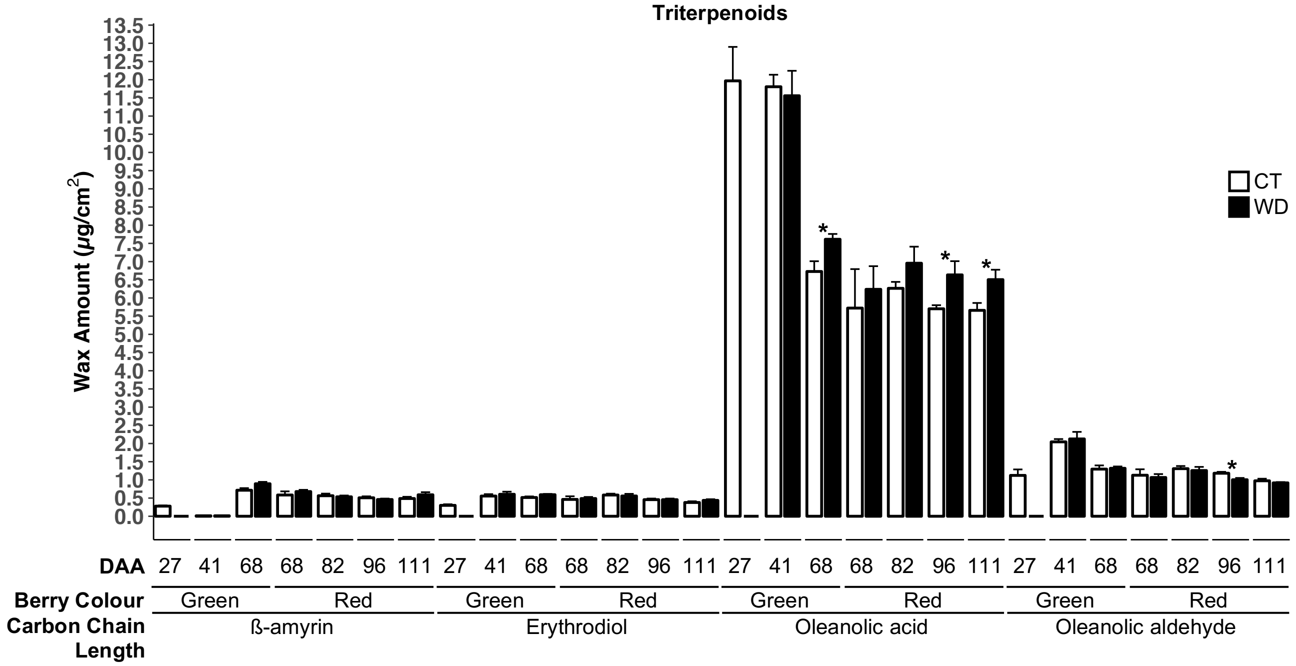

Figure S11. Cuticular wax composition in berries at 27, 41, 68, 82, 96, and 111 days after anthesis (DAA) of grapevines (*Vitis vinifera* L) exposed to two irrigation treatments: well-irrigated (Control = CT) and deficit irrigated (Water Deficit = WD). At 68 DAA, green berries were separated from red berries. Error bars represent ± S.E. and significant differences between treatments were determined by Two-Sample t-Test (*,$P<0.05$). Triterpenoids are made by the oleanolic acid pathway.

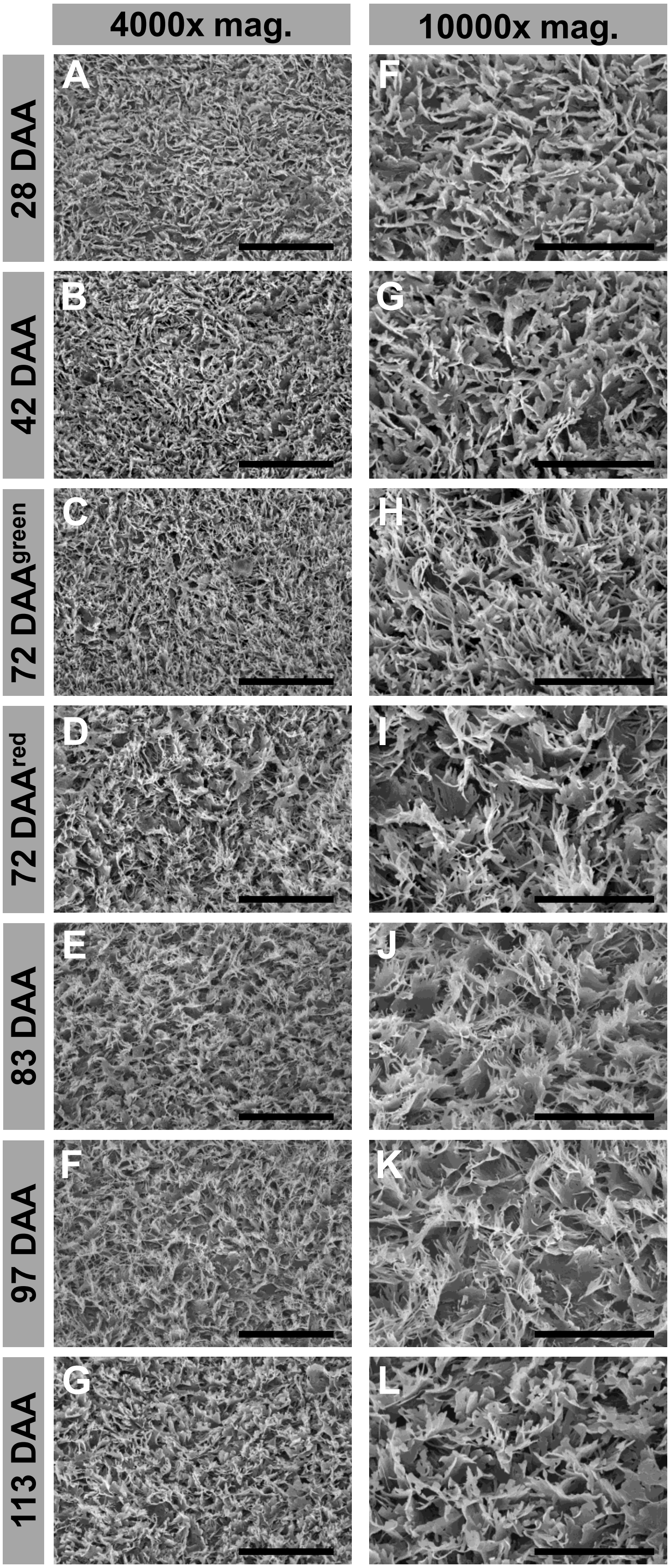

Figure S12: Ultrastructural morphology of cuticular waxes on grape berries during development. Scanning electron microscopy (SEM) images at 4000x (A-G) and 10000x (F-L) magnification of cuticular waxes on berries from 28 to 113 days after anthesis (DAA). At 72 DAA green and red berries were collected from the same clusters and analysed indepedently. Scale bar represents 10µm in A-G and 5µm in F-L.

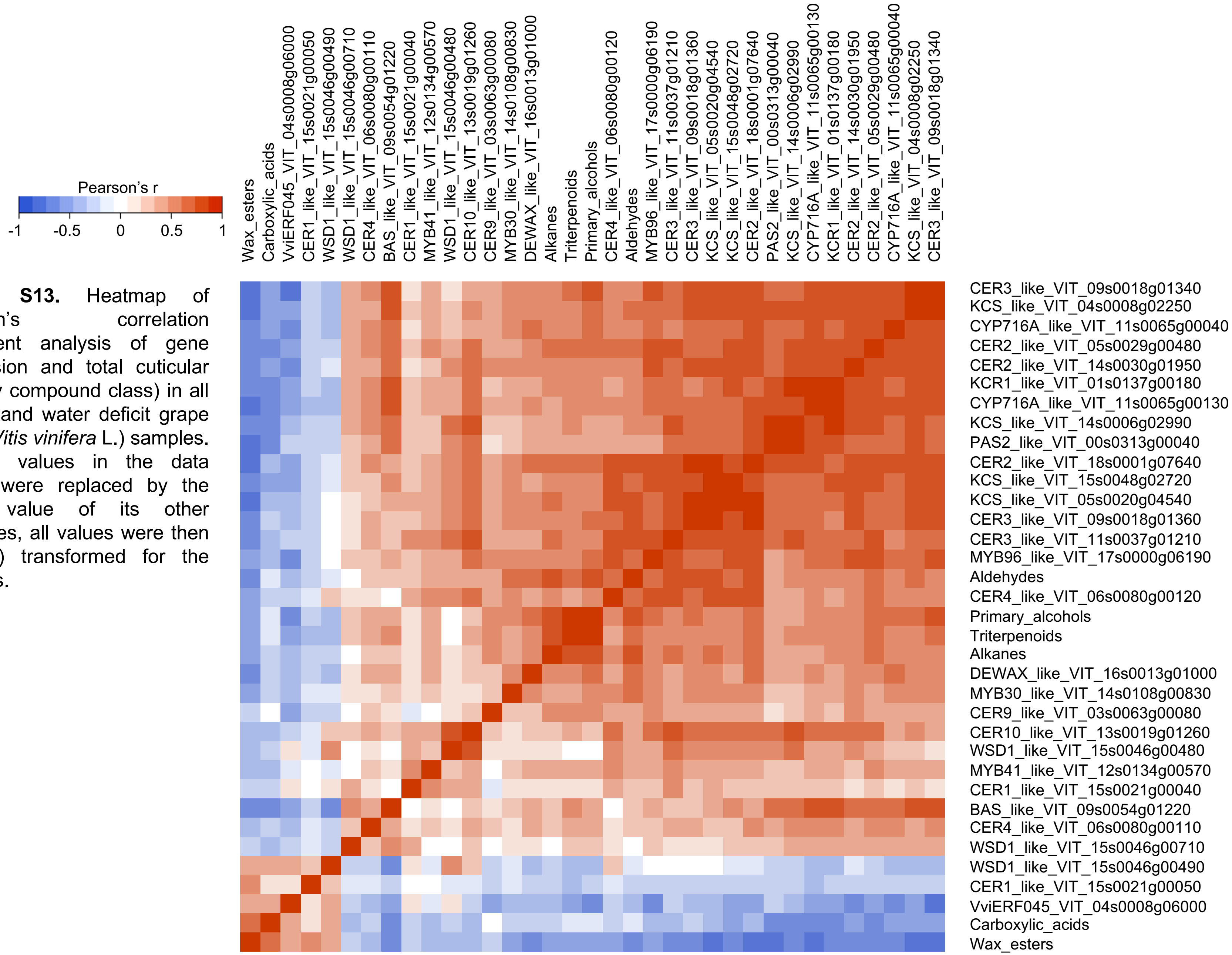

Figure S13. Heatmap of Pearson's correlation coefficient analysis of gene expression and total cuticular wax (by compound class) in all control and water deficit grape berry (*Vitis vinifera* L.) samples. Missing values in the data matrix were replaced by the mean value of its other replicates, all values were then log(x+1) transformed for the analysis.
