# Supplementary material for: Drought stress modulates cuticular wax composition of the grape berry
Source: J Exp Bot. 2020 Jan 27;71(10):3126–41. doi: 10.1093/jxb/eraa046 (PMC7260727; doi:10.1093/jxb/eraa046)
Supplement: eraa046_suppl_Supplementary_Tables_S1-S4 [file eraa046_suppl_supplementary_tables_s1-s4.pdf]

Table S1. List of genes that have been demonstrated to be involved in cuticular wax biosynthesis, oleanolic acid biosynthesis, and transcription factors involved in the regulation cuticular wax. The protein sequences of these genes were used as query sequences for BLASTp searches in grapevine and Arabidopsis genomes.

Gene Name	Pathway	Locus ID	Species	Reference
CER6	Aliphatic wax	At1g68530	*A. thaliana*	(Fiebig *et al.*, 2000)
KCR1	Aliphatic wax	At1g67730	*A. thaliana*	(Beaudoin *et al.*, 2009)
PAS2	Aliphatic wax	At5g10480	*A. thaliana*	(Bach *et al.*, 2008)
CER10	Aliphatic wax	At3g55360	*A. thaliana*	(Zheng *et al.*, 2005)
CER2	Aliphatic wax	At4g24510	*A. thaliana*	(Haslam et al. 2012)
CER2-LIKE1	Aliphatic wax	At4g13840	*A. thaliana*	(Haslam et al. 2015)
CER2-LIKE2	Aliphatic wax	At3g23840	*A. thaliana*	(Haslam et al. 2015)
CER1	Aliphatic wax	At1g02205	*A. thaliana*	(Bernard *et al.*, 2012)
CER3	Aliphatic wax	At5g57800	*A. thaliana*	(Bernard *et al.*, 2012)
CER4	Aliphatic wax	At4g33790	*A. thaliana*	(Rowland *et al.*, 2006)
WSD1	Aliphatic wax	At5g37300	*A. thaliana*	(Li *et al.*, 2008)
MAH1	Aliphatic wax	At1g57750	*A. thaliana*	(Greer *et al.*, 2007)
BAS	Oleanolic Acid	At1g78950	*A. thaliana*	(Shibuya *et al.*, 2009)
CYP716A12	Oleanolic Acid	MTR_8g100135	*M. truncatula*	(Fukushima *et al.*, 2011)
CYP716A15	Oleanolic Acid	-	*V. vinifera*	(Fukushima *et al.*, 2011)
CYP716A17	Oleanolic Acid	-	*V. vinifera*	(Fukushima *et al.*, 2011)
MYB96	Transcription Factor	At5g62470	*A. thaliana*	(Seo *et al.*, 2011)
MYB94	Transcription Factor	At3g47600	*A. thaliana*	(Lee *et al.*, 2016)
MYB30	Transcription Factor	At3g28910	*A. thaliana*	(Raffaele *et al.*, 2008)
MYB41	Transcription Factor	At4g28110	*A. thaliana*	(Cominelli *et al.*, 2008)
CER7	Transcription Factor	At3g60500	*A. thaliana*	(Lam *et al.*, 2015)
SHN1/WIN1	Transcription Factor	At1g15360	*A. thaliana*	(Kannangara *et al.*, 2007)
SHN2	Transcription Factor	At5g11190	*A. thaliana*	(Shi *et al.*, 2011)
SHN3	Transcription Factor	At5g25390	*A. thaliana*	(Shi *et al.*, 2011)
CER9	E3 Ubiquitin Ligase	At4g34100	*A. thaliana*	(Lu *et al.*, 2012)
MYB106	Transcription Factor	At3g01140	*A. thaliana*	(Oshima *et al.*, 2013)
MYB16	Transcription Factor	At5g15310	*A. thaliana*	(Oshima *et al.*, 2013)
CFL1	Transcription Factor	Os02g31140	*O. sativa*	(Wu *et al.*, 2011)
HDG1	Transcription Factor	At3g61150	*A. thaliana*	(Wu *et al.*, 2011)
WPX1	Transcription Factor	AEX93412	*M. truncatula*	(Zhang *et al.*, 2005)
WPX2	Transcription Factor	AEX93413	*M. truncatula*	(Zhang *et al.*, 2007)
WRI1	Transcription Factor	At3g54320	*A. thaliana*	(To *et al.*, 2012)
WRI3	Transcription Factor	At1g16060	*A. thaliana*	(To *et al.*, 2012)
WRI4	Transcription Factor	At1g79700	*A. thaliana*	(Park *et al.*, 2016)
ZmOCL1	Transcription Factor	GRMZM2G026643	*Z. mays*	(Javelle *et al.*, 2010)
VviERF045	Transcription Factor	VIT_04s0008g06000	*V. vinifera*	(Leida *et al.*, 2016)
BES1	Transcription Factor	At1g19350	*A. thaliana*	(Li *et al.*, 2009)
DEWAX	Transcription Factor	At5g61590	*A. thaliana*	(Go *et al.*, 2014)

Table S2. List of RNA-seq studies downloaded from the DNA data bank of Japan that were reprocessed to calculate transcript abundance and differential expression.

Accession	Description	Study Type	Tissue/organ type	Single or paired -end	Technology	Reference
SRP070855	Tocai friulano, water deficit	Abiotic Stress	Whole berry	Single-end	Illumina	(Savoi *et al.*, 2016)
SRP059734	Effect of Night/Day cycle, temperature, stages of development	Abiotic Stress	Berry skin and flesh	Paired-end	Illumina	(Rienth *et al.*, 2016)
SRP055458	Thompson seedless, gibberellic acid and shade treatments	Abiotic Stress	Inflorescence	Paired-end	Illumina	N/A
SRP032792	Rootstock M4 under water deficit, or salt stress	Abiotic Stress	Root, Leaf	Paired-end	ABI_SOLID	N/A
SRP057200	Semillon berries under *Botrytis* infection	Biotic Stress	Berry skin and flesh	Single-end	Illumina	(Blanco-Ulate *et al.*, 2015)
SRP049306	5 Italian cultivars, development	Development	Whole berry	Single-end	Illumina	(Palumbo *et al.*, 2014)
SRP046456	7 cultivars, late development	Development	Whole berry	Single-end	Illumina	N/A
SRP067690	Cabernet Sauvignon, development	Development	Berry skin	Paired-end	Illumina	N/A
SRP041212	*Vitis vinifera* and *Vitis sylvestris*, flower development	Development	Flower	Single-end	Illumina	(Ramos *et al.*, 2014)
SRP065417	Summer Black, leaf development	Development	Leaf	Single-end	Illumina	(Pervaiz *et al.*, 2016)

Table S3. Samples collected for generating cDNA libraries from roots, stems, buds, apexes, leaves, tendrils, flowers, berry skins, berry fleshes, and berry seeds at various developmental stages. Samples were collected from 20 two-year old, own-rooted Gewürztraminer grapevines grown in the UBC-Vancouver Horticulture Greenhouse.

Sample*	Description	Phenological stage at sampling
Root_Young	White, non lignified	E-L 33
Root_Lignified	Brown	E-L 33
Stem_Green	Non lignified	E-L 33
Bud_Green	Apical buds, non lignified	E-L 33
Bud_Lignified	Basal buds, lignified	E-L 33
Shoot_Apex	No unfolded leaves included	E-L 33
Leaf_Apical	Unfolded leaf 2-4 node from the apex	E-L 33
Leaf_Medium	Unfolded leaf 5-7 node from the apex	E-L 33
Leaf_Basal	Unfolded leaf 7-10 leaf from the apex	E-L 33
Tendril_Green	Non lignified	E-L 33
Flower_Blooming	Mix of open and closed flowers collected at blooming (50% open flowers)	E-L 23
Berry Skin_Pre-Veraison	Skins were separated from flesh and seeds of 15 berries	E-L 33
Berry Skin_Veraison	Skins were separated from flesh and seeds of 15 berries	E-L 35
Berry Skin_Post-Veraison	Skins were separated from flesh and seeds of 15 berries	E-L 38
Berry Flesh_Pre-Veraison	Flesh was separated from skin and seeds of 15 berries	E-L 33
Berry Flesh_Veraison	Flesh was separated from skin and seeds of 15 berries	E-L 35
Berry Flesh_Post-Veraison	Flesh was separated from skin and seeds of 15 berries	E-L 38
Berry Seed_Pre-Veraison	Seeds were separated from skins and flesh of 15 berries	E-L 33
Berry Seed_Veraison	Seeds were separated from skins and flesh of 15 berries	E-L 35
Berry Seed_Post-Veraison	Seeds were separated from skins and flesh of 15 berries	E-L 38

* Three biological replicates were collected for each sample type.

Table S4. Primer sequences of *V. vinifera* L. of likeliest candidate genes involved in the cuticular wax biosynthetic pathway, oleanolic acid biosynthetic pathway, E3 ubiquitin ligase and transcription factors. Selected genes were used for RT-qPCR analysis in tissue expression atlas and water deficit experiement.

Gene Family	Pathway	Grapevine_ID	Forward Primer 5'->3' sequence	Reverse Primer 5'->3' sequence	Product Size
CER1-like	Aliphatic wax	***VIT_15s0021g00040***	GCAAAATGAGCCCATCAATA	TGCCTAAACTCAACACTTTAACA	83
		VIT_15s0021g00050	CGTCCTTTAATGATTCCCTCTT	CATTCCAGATACTAGCACCAAA	79
CER10-like	Aliphatic wax	***VIT_13s0019g01260***	CAACTTCACCGCTCTCTAATG	ACCAACCCAAACCCAAAC	142
CER2-like	Aliphatic wax	***VIT_18s0001g07640***	GAAGACGGTGTGTGAGAATG	CCCAAACTGGCTTATGTCC	155
		VIT_05s0029g00480	CGAAGCCAAGTGCGATAA	CAACTCAGGACCGACGAT	100
		VIT_14s0030g01950	TGGTGAACTGTAATGATGCC	TCCTTCAAATTCGTTTATCTGG	179
CER3-like	Aliphatic wax	***VIT_09s0018g01340***	GGAATTATATGTTGTATGGTGCTC	CCACTGTAAAAGGTTGTGTCAC	145
		VIT_09s0018g01360	AGAAACTCTATCAAGGCAAACC	ACATAGGAGGAGAATATGTAGGC	175
		VIT_11s0037g01210	GAGGGCATCAACAATCAAATAG	GGTGCTTGTCAACAAAGAGG	133
CER4-like	Aliphatic wax	***VIT_06s0080g00110***	GGGAAGAGATTTGGAGAGAAG	CTGAGACATAAGCAGTGGATAC	180
		VIT_06s0080g00120	CTTGCTATTCTTCGCCCTAC	GATTCCTTCAACCCAACCT	72
CER6-like	Aliphatic wax	***VIT_04s0008g02250***	TCATACCCAACTGTTTCTTCC	CTTCTGTCATCAGCACCTTTA	130
		VIT_05s0020g04540	GCTAAAGGGAGGATCAGAAAG	GACTGGGAACTCATCAATCTC	153
		VIT_14s0006g02990	ATGGTAGGTGTTGTGATTGAG	CGAGGCTTGGACATAAAGTAG	149
		VIT_15s0048g02720	GAGAGAGAGAGATGGGTAGTATAG	CGGATGACGAGTCCTTAAAC	78
KCR1-like	Aliphatic wax	***VIT_01s0137g00180***	GGTTCAGGTGCTGCTATT	CGATATACGCTTTTGTGGC	73
PAS2-like	Aliphatic wax	***VIT_00s0313g00040***	CACTTCTAAGGCGTCTGTATC	CCCGATTCTTTCAGAGTTGA	100
WSD1-like	Aliphatic wax	***VIT_15s0046g00480***	CAGGTTAAGGACGATGACAAA	GAGGTCGGAGATGTAGTCTT	141
		VIT_15s0046g00490	GAAGTTGGGTTTTCTGGTCA	GGTGAGGATCGGGAACAAT	148
		VIT_15s0046g00710	GCTGAAGGCAAGAAGAATAAAG	GGATAAAGGGAGAAGGACATAG	189
BAS-like	Oleanolic acid	***VIT_09s0054g01220***	TCATCACGTATGCTGCTAAGTA	CACCATCATCCCTCTGTGAA	198
CYP716A-like	Oleanolic acid	***VIT_11s0065g00040***	CATCAGACTTTACTTGCTCTTATAC	AATTCAAGGCTTTCACCAACC	105
		VIT_11s0065g00130	GATGCCACATGAATGAAATGGA	ATTTGCTCCTCGTAGACTT	148
CER9-like	E3 Ubiquitin Ligase	***VIT_03s0063g00080***	TTGCGGACAACGATCAAA	CAGGTCTAGGCAGTAAGAAATC	91
DEWAX-like	Transcription Factor	***VIT_16s0013g01000***	AGAGAGCAAGCAGAGACTAA	CATTAACGGACTGATCCCAAA	102
MYB30-like	Transcription Factor	***VIT_14s0108g00830***	GACCACCTTGCTGTGATAAA	GGACCATGTTCCTGGATATAAG	94
MYB41-like	Transcription Factor	***VIT_12s0134g00570***	ATTCATCGTCCACCTTCATC	AACTCTCGGGAATCTCAAAC	90
MYB96-like; MYB94-like	Transcription Factor	***VIT_17s0000g06190***	ATCAGGAGGAGAAGACGATAA	TCCGTTCTTTGAGGAAGATAAG	88
VviERF045	Transcription Factor	***VIT_04s0008g06000***	TTGAGGAGTTGCTTGACTATG	GGAATACAGAGAGAGAAGAGGA	110

References for supplementary tables

Bach L, Michaelson L V, Haslam R, *et al.* 2008. The very-long-chain hydroxy fatty acyl-CoA dehydratase PASTICCINO2 is essential and limiting for plant development. Proceedings of the National Academy of Sciences of the United States of America **105**, 14727–31.

Beaudoin F, Wu X, Li F, Haslam RP, Markham JE, Zheng H, Napier JA, Kunst L. 2009. Functional characterization of the Arabidopsis b-ketoacyl-coenzyme A reductase candidates of the fatty acid elongase. Plant Physiology **150**, 1174–1191.

Bernard A, Domergue F, Pascal S, Jetter R, Renne C, Faure J-D, Haslam RP, Napier JA, Lessire R, Joubes J. 2012. Reconstitution of plant alkane biosynthesis in yeast demonstrates that *Arabidopsis* ECERIFERUM1 and ECERIFERUM3 are core components of a very-long-chain alkane synthesis complex. The Plant Cell **24**, 3106–3118.

Blanco-Ulate B, Amrine KC, Collins TS, *et al.* 2015. Developmental and metabolic plasticity of white-skinned grape berries in response to Botrytis cinerea during noble rot. Plant Physiology **169**, 2422–2443.

Cominelli E, Sala T, Calvi D, Gusmaroli G, Tonelli C. 2008. Over-expression of the Arabidopsis AtMYB41 gene alters cell expansion and leaf surface permeability. Plant Journal **53**, 53–64.

Fiebig A, Mayfield JA, Miley NL, Chau S, Fischer RL, Preuss D. 2000. Alterations in *CER6*, a gene identical to *CUT1*, differentially affect long-chain lipid content on the surface of pollen and stems. The Plant Cell **12**, 2001–2008.

Fukushima EO, Seki H, Ohyama K, Ono E, Umemoto N, Mizutani M, Saito K, Muranaka T. 2011. CYP716A subfamily members are multifunctional oxidases in triterpenoid biosynthesis. Plant and Cell Physiology **52**, 2050–2061.

Go YS, Kim H, Kim HJ, Suh MC. 2014. *Arabidopsis* Cuticular Wax Biosynthesis Is Negatively Regulated by the *DEWAX* Gene Encoding an AP2/ERF-Type Transcription Factor. The Plant Cell **26**, 1666–1680.

Greer S, Wen M, Bird D, Wu X, Samuels L, Kunst L, Jetter R. 2007. The cytochrome P450 enzyme CYP96A15 is the midchain alkane hydroxylase responsible for formation of secondary alcohols and ketones in stem cuticular wax of Arabidopsis. Plant Physiology **145**, 653–667.

Haslam TM, Haslam R, Thoraval D, *et al.* 2015. ECERIFERUM2-LIKE proteins have unique biochemical and physiological functions in very-long-chain fatty acid elongation. Plant Physiology **167**, 682–692.

Haslam TM, Mañas-Fernández A, Zhao L, Kunst L. 2012. Arabidopsis ECERIFERUM2 is a component of the fatty acid elongation machinery required for fatty acid extension to exceptional lengths. Plant Physiology **160**, 1164–74.

Javelle M, Vernoud V, Depège-Fargeix N, Arnould C, Oursel D, Domergue F, Sarda X, Rogowsky PM. 2010. Overexpression of the epidermis-specific homeodomain-leucine zipper IV transcription factor OUTER CELL LAYER1 in maize identifies target genes involved in lipid metabolism and cuticle biosynthesis. Plant Physiology **154**, 273–286.

Kannangara R, Branigan C, Liu Y, Penfield T, Rao V, Mouille G, Hofte H, Pauly M, Riechmann JL, Broun P. 2007. The transcription factor WIN1/SHN1 regulates cutin biosynthesis in *Arabidopsis thaliana*. the Plant Cell Online **19**, 1278–1294.

Lam P, Zhao L, Eveleigh N, Yu Y, Chen X, Kunst L. 2015. The exosome and trans-acting small interfering RNAs regulate cuticular wax biosynthesis during Arabidopsis inflorescence stem development. Plant Physiology **167**, 323–336.

Lee SB, Kim HU, Suh MC. 2016. MYB94 and MYB96 additively activate cuticular wax biosynthesis in Arabidopsis. Plant and Cell Physiology **57**, 2300–2311.
Leida C, Dal Rì A, Dalla Costa L, Gómez MD, Pompili V, Sonego P, Engelen K, Masuero D, Ríos G, Moser C. 2016. Insights into the role of the berry-specific ethylene responsive factor *VviERF045*. Frontiers in Plant Science **7**.
Li F, Wu X, Lam P, Bird D, Zheng H, Samuels L, Jetter R, Kunst L. 2008. Identification of the wax ester synthase/acyl-coenzyme A: diacylglycerol acyltransferase WSD1 required for stem wax ester biosynthesis in Arabidopsis. Plant Physiology **148**, 97–107.
Li L, Yu X, Thompson A, Guo M, Yoshida S, Asami T, Chory J, Yin Y. 2009. Arabidopsis MYB30 is a direct target of BES1 and cooperates with BES1 to regulate brassinosteroid-induced gene expression. Plant Journal **58**, 275–286.
Lu S, Zhao H, Des Marais DL, *et al.* 2012. Arabidopsis ECERIFERUM9 involvement in cuticle formation and maintenance of plant water status. Plant Physiology **159**, 930–944.
Oshima Y, Shikata M, Koyama T, Ohtsubo N, Mitsuda N, Ohme-Takagi M. 2013. MIXTA-like transcription factors and WAX INDUCER1/SHINE1 coordinately regulate cuticle development in *Arabidopsis* and *Torenia fournieri*. The Plant Cell **25**, 1609–1624.
Palumbo MC, Zenoni S, Fasoli M, Massonnet M, Farina L, Castiglione F, Pezzotti M, Paci P. 2014. Integrated network analysis identifies fight-club nodes as a class of hubs encompassing key putative switch genes that induce major transcriptome reprogramming during grapevine development. The Plant Cell **26**, 4617–4635.
Park CS, Go YS, Suh MC. 2016. Cuticular wax biosynthesis is positively regulated by WRINKLED4, an AP2/ERF-type transcription factor, in Arabidopsis stems. Plant Journal **88**, 257–270.
Pervaiz T, Haifeng J, Haider MS, Cheng Z, Cui M, Wang M, Cui L, Wang X, Fang J. 2016. Transcriptomic analysis of grapevine (cv. Summer Black) leaf, using the illumina platform. PLoS ONE **11**, 1–20.
Raffaele S, Vailleau F, Leger A, Joubes J, Miersch O, Huard C, Blee E, Mongrand S, Domergue F, Roby D. 2008. A MYB transcription factor regulates very-long-chain fatty acid biosynthesis for activation of the hypersensitive cell death response in *Arabidopsis*. The Plant Cell **20**, 752–767.
Ramos MJ, Coito J, Silva H, Cunha J, Costa MM, Rocheta M. 2014. Flower development and sex specification in wild grapevine. BMC Genomics **15**, 1095.
Rienth M, Torregrosa L, Sarah G, Ardisson M, Brillouet J-M, Romieu C. 2016. Temperature desynchronizes sugar and organic acid metabolism in ripening grapevine fruits and remodels their transcriptome. BMC Plant Biology **16**, 164.
Rowland O, Zheng H, Hepworth SR, Lam P, Jetter R, Kunst L. 2006. *CER4* encodes an alcohol-forming fatty acyl-coenzyme A reductase involved in cuticular wax production in Arabidopsis. Plant Physiology **142**, 866–877.
Savoi S, Wong DCJ, Arapitsas P, Miculan M, Bucchetti B, Peterlunger E, Fait A, Mattivi F, Castellarin SD. 2016. Transcriptome and metabolite profiling reveals that prolonged drought modulates the phenylpropanoid and terpenoid pathway in white grapes (*Vitis vinifera* L.). BMC Plant Biology **16**, 67.
Seo PJ, Lee SB, Suh MC, Park M-J, Go YS, Park C-M. 2011. The MYB96 transcription factor regulates cuticular wax biosynthesis under drought conditions in *Arabidopsis*. The Plant Cell **23**, 1138–1152.
Shi JX, Malitsky S, de Oliveira S, Branigan C, Franke RB, Schreiber L, Aharoni A. 2011.

SHINE transcription factors act redundantly to pattern the archetypal surface of arabidopsis flower organs. PLoS Genetics **7**.
Shibuya M, Katsube Y, Otsuka M, Zhang H, Tansakul P, Xiang T, Ebizuka Y. 2009. Identification of a product specific ß-amyrin synthase from *Arabidopsis thaliana*. Plant Physiology and Biochemistry **47**, 26–30.
To A, Joubes J, Barthole G, Lecureuil A, Scagnelli A, Jasinski S, Lepiniec L, Baud S. 2012. WRINKLED transcription factors orchestrate tissue-specific regulation of fatty acid biosynthesis in Arabidopsis. The Plant Cell **24**, 5007–5023.
Wu R, Li S, He S, Wassmann F, Yu C, Qin G, Schreiber L, Qu L-J, Gu H. 2011. CFL1, a WW domain protein, regulates cuticle development by modulating the function of HDG1, a class IV homeodomain transcription factor, in rice and *Arabidopsis*. The Plant Cell\ **23**, 3392–3411.
Zhang JY, Broeckling CD, Blancaflor EB, Sledge MK, Sumner LW, Wang ZY. 2005. Overexpression of WXP1, a putative *Medicago truncatula* AP2 domain-containing transcription factor gene, increases cuticular wax accumulation and enhances drought tolerance in transgenic alfalfa (*Medicago sativa*). Plant Journal **42**, 689–707.
Zhang JY, Broeckling CD, Sumner LW, Wang ZY. 2007. Heterologous expression of two *Medicago truncatula* putative ERF transcription factor genes, WXP1 and WXP2, in *Arabidopsis* led to increased leaf wax accumulation and improved drought tolerance, but differential response in freezing tolerance. Plant Molecular Biology **64**, 265–278.
Zheng H, Rowland O, Kunst L. 2005. Disruptions of the Arabidopsis Enoyl-CoA reductase gene reveal an essential role for very-long-chain fatty acid synthesis in cell expansion during plant morphogenesis. The Plant Cell **17**, 1467–81.
